# Longitudinal Observation by Optical Coherence Tomography in Patients Treated with Ethambutol: A Systematic Review and Meta-Analysis

**DOI:** 10.3390/jcm15031230

**Published:** 2026-02-04

**Authors:** Rui Luo, Jin Ma, Yong Zhong

**Affiliations:** 1 Department of Ophthalmology, Peking Union Medical College Hospital, Chinese Academy of Medical Sciences and Peking Union Medical College, Beijing 100730, China; 2 Beijing Key Laboratory of Fundus Diseases Intelligent Diagnosis & Drug/Device Development and Translation, Beijing 100730, China

**Keywords:** ethambutol, tuberculosis, optic coherence tomography, retinal nerve fiber layer, ganglion cell layer and inner plexiform layer

## Abstract

**Background**: The retinal changes caused by ethambutol are not clear in patients with the administration of ethambutol and without ethambutol-induced optic neuropathy (EON). The aim of this systematic review is to estimate the changes in retinal nerve fiber layer (RNFL) and ganglion cell layer and inner plexiform layer (GCIPL) thicknesses measured by optical coherence tomography (OCT) in patients with mycobacterial infection treated with ethambutol and not suffering from EON. **Methods**: A systematic review of articles was conducted by searching PubMed, Embase, and Web of Science until November 2025. Additional studies were identified by the review of references. Search terms included OCT and ethambutol. Longitudinal observational studies using an OCT device to measure RNFL and GCIPL thicknesses before and after the administration of ethambutol in patients with mycobacterial infection without ocular diseases were included. The extraction of data in studies was performed by two researchers using data extraction sheets. The meta-analysis was conducted using the random-effect model. **Results**: In total, 14 studies (*n* = 1138) were eligible for the systematic review. Meta-analysis combining RNFL measured after the longest duration of ethambutol administration showed no significant decrease compared to RNFL before treatment. However, there were significant decreases in RNFL thickness in male-dominant studies, studies conducted in Turkey and India, and studies conducted by the Cirrus OCT device. In addition, the decreases in RNFL thickness were correlated with the duration of ethambutol administration in male-dominant studies. Only two studies reported the thickness changes in GCIPL, and the study with a higher male proportion showed significant decreases in GCIPL thickness. **Conclusions**: Ethambutol does not cause a significant RNFL decrease generally in mycobacterial infection patients; however, it may lead to decreased RNFL thickness in male patients and patients in some regions, even though they do not suffer from EON.

## 1. Introduction

Ethambutol is an effective antibiotic broadly used in the treatment of mycobacterial infection, especially tuberculosis. However, toxic optic neuropathy is one of the most common severe adverse effects caused by this drug. The reported incidence of ethambutol-induced optic neuropathy (EON) varies from 0.25% to 6.5% in different retrospective observational studies [1,2,3,4,5,6]. Patients with EON present with decreased visual acuity, abnormal color vision, optic disc pallor, and visual field defects [7], which usually show bilateral involvement and are dose- and duration-dependent [4,6].

Since there is no effective treatment for EON [8], the prognosis of EON is poor. A retrospective study conducted by Valenchia and colleagues [9] showed that visual improvement was observed in only 56.9% of patients with EON despite cessation of ethambutol, and the proportion was lower in old patients and patients with hypertension or kidney diseases. The visual outcome is worse in patients with a delay of more than a month from symptom onset and the drug cessation [10]. On the contrary, subclinical ocular lesions caused by ethambutol are reversible after the discontinuation of the drug [11]. Therefore, a thorough understanding of subclinical changes related to ethambutol may help improve the screening strategy and reduce the burden of EON.

The changes in several functional indicators in patients treated with ethambutol have been studied in previous research. Changes in visual evoked potentials and multifocal electroretinography were observed in patients with the administration of ethambutol and without EON; however, changes in visual acuity, color vision, contrast sensitivity, and visual field were not significant [8,11,12]. Although electrophysiological indicators may be able to detect EON early, these examinations are time-consuming and not appropriate for screening subclinical EON.

Optical coherence tomography (OCT) is a noninvasive and noncontactable device and can detect retinal lesions quantitatively; thus, it is broadly used in the evaluation of many ocular diseases, such as glaucoma [13], uveitis [14], retinopathy [15], and optic neuritis [16]. Ocular lesions related to ethambutol were also detected by OCT in previous studies, which reported decreased thicknesses in both retinal nerve fiber layer (RNFL) and ganglion cell layer and inner plexiform layer (GCIPL) in patients with EON [17,18,19]. In addition, Lee and colleagues [20] found a good diagnostic performance of GCIPL thicknesses in the diagnosis of EON, and Kang and colleagues [21] reported the prognostic value of RNFL and GCIPL in the visual function of EON patients. However, the results of the OCT changes are controversial in patients treated with ethambutol and not suffering from EON, leading to difficulties in the evaluation of subclinical toxicity related to ethambutol.

Therefore, in the present meta-analysis, we aimed to compare the changes in RNFL and GCIPL thicknesses detected by OCT before and after the administration of ethambutol in patients with mycobacterial infection.

## 2. Materials and Methods

This systematic review and meta-analysis were conducted in accordance with Preferred Reporting Items for Systematic Reviews and Meta-Analyses COnsensus-based Standards for the selection of health Measurement INstruments (PRISMA-COSMIN) for outcome measurement instruments (OMIs) 2024 (checklist shown in Table S1) [22] and registered at PROSPERO with the number CRD420251234154.

### 2.1. Selection Criteria

Studies eligible for this meta-analysis are required to meet the following criteria: (1) participants included in the study must be patients with mycobacterial infection treated with ethambutol; and (2) the study must be a longitudinal observational study in which the thicknesses of RNFL or GCIPL were measured by OCT before both the administration and the cessation of ethambutol. Articles were excluded if they were reviews, case reports, or case series with fewer than 10 participants, or if they observed patients with EON or other ocular diseases before the administration of ethambutol.

### 2.2. Search Strategy and Study Selection

We used the following terms to search the electronic databases of PubMed, Embase, and Web of Science (up to November 2025): “OCT”, “optical coherence tomography”, “RNFL”, “GCIPL”, “retinal nerve fiber”, “ganglion cell”, and “ethambutol”. The Medical Subject Headings (MeSH) and other related keywords were used in the search. Additional studies were identified by the review of references. No limits were applied for languages, and papers written in languages except English were translated. There were also no publication status or follow-up period restrictions.

After the deletion of duplicates, the titles and abstracts of all records obtained by the searches were screened to omit reviews, case reports, and studies not related to OCT. The full text of the remaining records was then viewed to select studies eligible for the meta-analysis. All the evaluation of eligibility was performed in an unblinded manner by 2 reviewers, and disagreement was solved by consulting a senior researcher.

### 2.3. Data Collection and Risk of Bias Assessment

We developed a data extraction sheet to collect data, which was revised after the test on 3 studies selected randomly. We extracted the following information: the first author; the publication year; country; sample size; the proportion of males; the mean age; the dose of ethambutol; the type of OCT devices; the duration of follow-up; and the mean and standard deviation (SD) of the thickness of RNFL, and GCIPL on average and in different subsectors. The data collection was performed by 2 researchers, and disagreement was solved through discussion between the 2 researchers.

The risk of bias of studies included in this meta-analysis was evaluated by using the criteria recommended by the risk of bias in non-randomized studies of interventions (ROBINS-I) [23].

### 2.4. Data Analysis

The primary outcome measure of this study was the thickness of retinal layers before and after the administration of ethambutol. The meta-analysis was performed if there were 3 or more studies measuring the thicknesses. We conducted the meta-analysis by calculating the standardized mean differences (SMDs) and 95% confidence intervals (CIs) between the retinal layer thicknesses before and after the ethambutol administration using the random-effect model. If the retinal layer thicknesses were measured several times after the ethambutol administration, data obtained after the longest administration period were included in the primary analysis. For studies reporting data for the left and right eyes separately, the data were synthesized by meta-analysis using the random-effect model, and the results were used in the primary analysis. The inconsistency of included studies was evaluated by the I^2^. The risk of bias across studies was assessed by evaluating the funnel plots, and Egger’s test was performed to reduce the subjectivity of the visual evaluation.

We performed a meta-regression to explore the association between the thicknesses of retinal layers and continuous study characteristics, such as the male proportions, the mean ages, and the periods of the ethambutol administration. In addition, subgroup analysis was conducted to evaluate the association between the thicknesses and categorial study characteristics, including countries. Continuous characteristics found to be significantly associated with the thicknesses were split to categorial characteristics, which were included in the subgroup analysis subsequently.

To explore the association between thickness changes in retinal layers and the duration of the ethambutol administration, we performed meta-analyses focusing on retinal layer thicknesses measured after the same period of the ethambutol administration if the thicknesses were measured by more than 3 studies after that period. Further evaluation of retinal layer thickness changes and ethambutol administration periods was conducted by linear regression, analyzing the correlation between the meta-analysis results and the time periods.

All statistical analyses were performed using R software (version 4.5.2) and the package named meta (version 8.2-1) [24]. Continuous variants distributed normally were shown as mean ± standard deviation. Two-sided *p*-value of less than 0.05 was considered to indicate statistical significance.

## 3. Results

### 3.1. Study Selection

**Figure 1:**
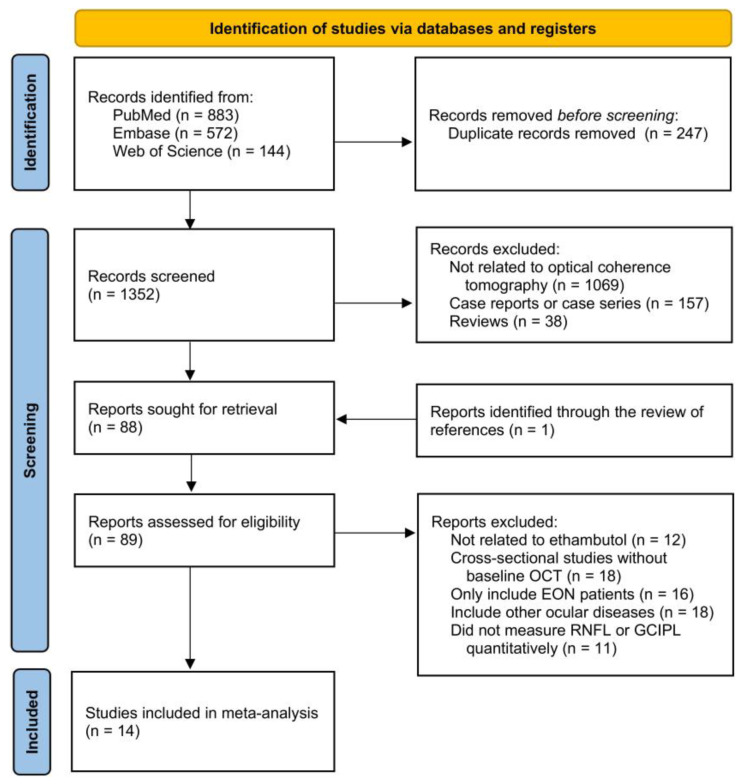
Preferred Reporting Items for Systematic Reviews and Meta-Analyses (PRISMA) flowchart. EON, ethambutol optic neuropathy; OCT, optical coherence tomography; RNFL, retinal nerve fiber layer; GCIPL, ganglion cell layer and inner plexiform layer.

Figure 1 summarizes the selection process of studies included in this meta-analysis. The search of PubMed, Embase, and Web of Science electronic databases provided 1599 citations in total, 1352 of which remained after the deletion of duplicates. In total, 1264 records were omitted after the screening of abstracts since these studies were reviews or case reports, or not related to OCT. The full text of the remaining 88 studies, as well as another study identified through the review of references, was reviewed in detail. Seventy-five of them did not meet the selection criteria and thus were removed. The remaining 14 studies were eligible for this systematic review and meta-analysis [11,25,26,27,28,29,30,31,32,33,34,35,36,37]. No unpublished relevant studies were obtained.

### 3.2. Study Characteristics and Risk of Bias

Table 1 summarizes the characteristics of studies included in this meta-analysis. The included studies involved 1138 eyes of tuberculosis patients treated with ethambutol. Five and four studies were conducted in India and Korea, respectively, and the other studies were conducted in Turkey, Indonesia, Brazil, and Malaysia. The male proportion and mean age were 0.4 to 0.771 and 6.5 to 46.4 years, respectively. The daily doses of ethambutol ranged from 14.72 to 20 mg/kg, and the periods of follow-up ranged from 2 to 8 months. All 12 studies measured RNFL thicknesses, and GCIPL thicknesses were measured in 2 studies conducted by Han, Mandal, and their colleagues [29,33].

The risk of bias was evaluated using the criteria recommended by ROBINS-I. Most of the studies included in the systematic review were at low risk of bias (Table S2).

### 3.3. RNFL Thickness Changes After the Longest Periods of Ethambutol Administration

**Figure 2:**
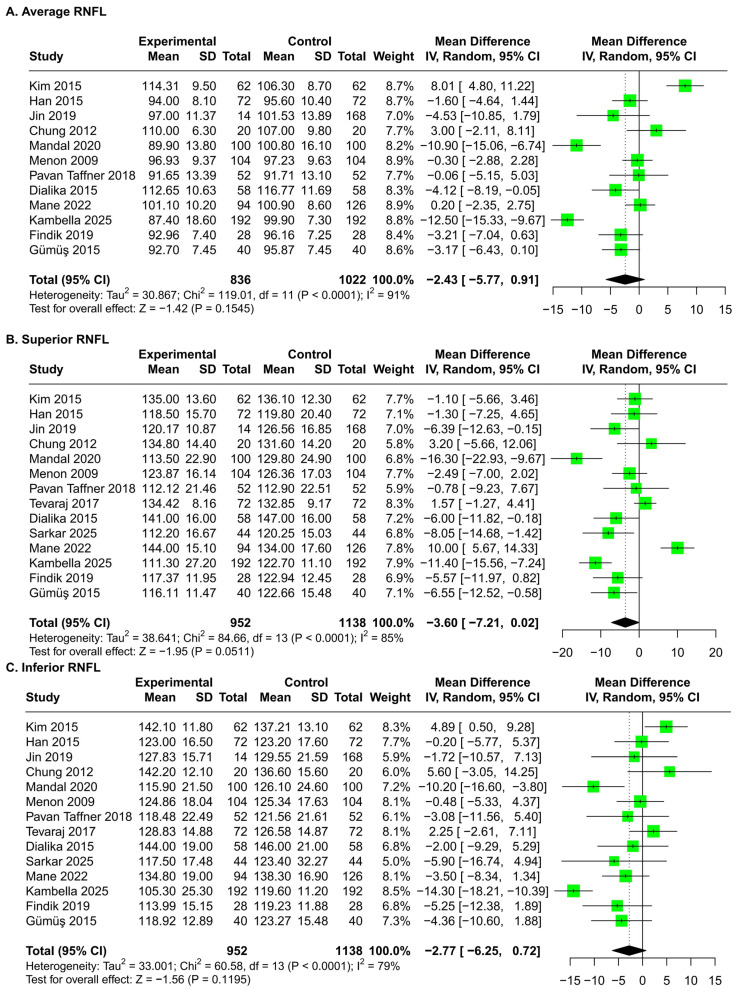

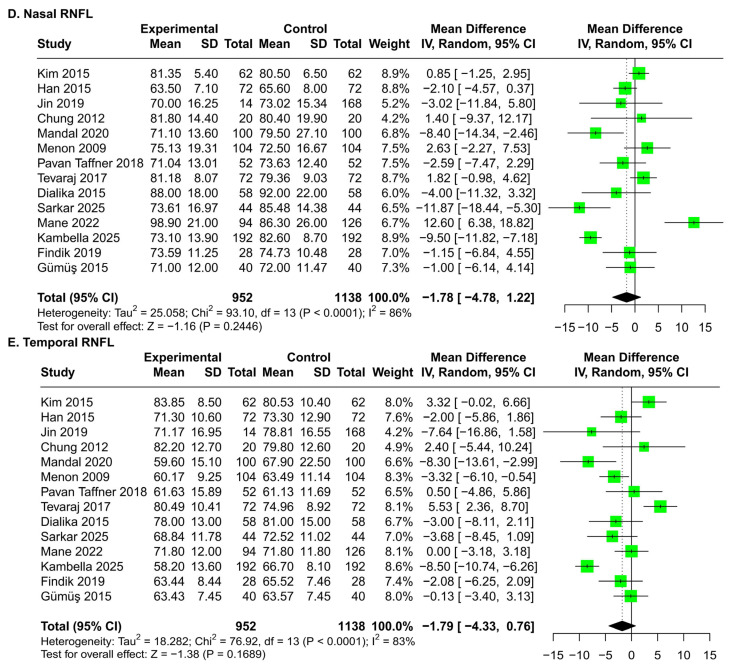
Meta-analysis of RNFL thicknesses before and after the ethambutol administration in average [11,25,26,27,28,29,30,31,32,33,34,35,36,37] (**A**) and in the superior (**B**), inferior (**C**), nasal (**D**), and temporal (**E**) subsectors. The sizes of green squares, black crosses, and vertical lines indicate the weights, mean differences, and 95% CIs in included studies, respectively. The dashed lines indicate the standardized mean differences obtained after meta-analysis. The sample sizes refer to the number of eyes. RNFL, retinal nerve fiber layer; SD, standard deviation; IV, inverse variance; CI, confidence interval.

**Figure 3:**
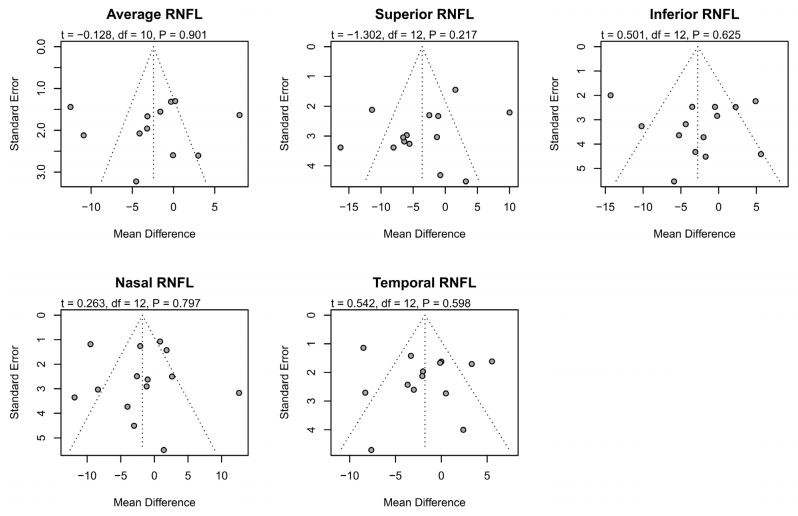
Publication bias of the meta-analysis combining RNFL data. The t-values, degrees of freedom, and *p*-values obtained in the Egger’s tests are shown at the top of the funnel plots. RNFL = retinal nerve fiber layer; df = degree of freedom.

No significant changes in RNFL thicknesses occurred in patients after the ethambutol administration compared with baseline (Figure 2), and funnel plots and Egger’s test did not show publication bias (Figure 3).

### 3.4. Factors Associated with Changes in RNFL Thicknesses

**Figure 4:**
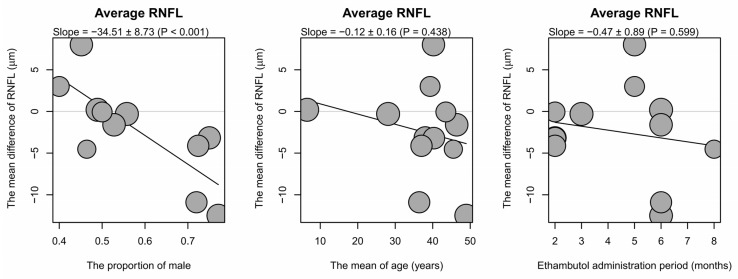
Bubble plots obtained in the meta-regression focusing on the relationship between RNFL thickness on average and the proportion of males, the mean age, and the period of the ethambutol administration. The sizes of the bubbles refer to the weights of the studies, and the black lines are obtained in the regression, while the gray lines represent the mean difference of 0. The slopes of the black lines are shown at the top of the bubble plots as estimates ± standard errors, followed by *p*-values. The results of meta-regression focusing on RNFL thicknesses in subsectors are shown in Table 3 and Figure S1. RNFL = retinal nerve fiber layer.

The association between male proportions, mean ages, ethambutol administration periods, and RNFL thickness changes was analyzed by the meta-regression (Figure 4 and Figure S1; Table 2). The association between the RNFL thickness changes and ethambutol dose was not included in the analysis due to the similarity of the dosages in these studies. The results of the analysis revealed a significantly negative correlation between the male proportion and the changes in average, superior, inferior, and nasal RNFL thicknesses, while the temporal subsector showed an insignificant trend. There was a significantly negative association between superior and nasal RNFL thicknesses and the mean age. The period of the ethambutol administration was not significantly correlated with RNFL thickness changes.

The association between countries and the changes in RNFL thicknesses was analyzed by subgroup analysis. Since male proportion and mean age showed significant influences on the RNFL thickness changes, we divided the studies with cut-off values of 2/3 in the male proportion and 40 in the mean age, and we conducted subgroup analysis subsequently. Table 3 and Figure S2 show the results of subgroup analysis. There was a statistically significant decrease in RNFL thicknesses of all subsectors in male-dominant studies and not in female-dominant studies, and the difference between the subgroups was significant in all subsectors except temporal RNFL. The meta-analysis synthesizing studies from Turkey showed a significant decrease in average, inferior, and superior RNFL thicknesses, and results from Indian studies revealed a significant decrease in inferior and temporal RNFL thicknesses. However, the difference among countries was significant only in the inferior RNFL. The RNFL decreases were significant in all subsectors except the temporal RNFL in studies conducted with Cirrus (Carl Zeiss Meditec, Dublin, CA, USA), and the difference among subgroups was significant in the inferior and nasal RNFL. There was no significant difference between subgroups with different mean ages.

### 3.5. RNFL Thickness Changes After the Same Periods of the Ethambutol Administration

**Figure 5:**
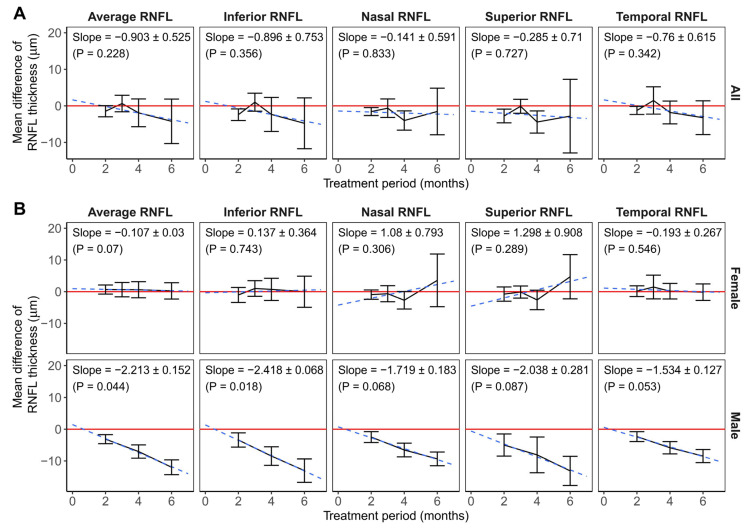
The relationship between RNFL thicknesses and the period of the ethambutol administration in (**A**) all studies and (**B**) studies with different dominant sexes. The lines connect the estimation of mean differences of RNFL thicknesses, and the error bars show the 95% CI obtained in the meta-analysis. Red lines highlight the difference of 0. Blue dashed lines refer to the results of the linear regression, and the slopes of them are shown at the top of the figures as estimation ± standard error, followed by the *p*-values. RNFL = retinal nerve fiber layer; OCT = optical coherence tomography.

Since RNFL thicknesses 2, 3, 4, and 6 months after the start of the ethambutol administration were reported in more than three studies, we conducted meta-analyses comparing the thicknesses measured at these times and at baseline (Figure 5A and Figure S4, and Table S3). Superior, inferior, nasal, and temporal RNFL thicknesses measured at month 2, and the superior and nasal RNFL thicknesses measured at month 4 showed significant changes when compared with baseline, and the changes in RNFL thicknesses in other subsectors and after other periods of ethambutol administration were not significant.

RNFL thicknesses measured at all time points and subsectors were significantly different from the baseline in male-dominant studies, while meta-analysis combining female-dominant studies did not show significant changes in RNFL thicknesses (Figure 5B and Table S3).

Linear regression analyzing all studies did not show a significant association between RNFL thicknesses and the ethambutol administration period. However, linear regression including male-dominant studies revealed significant decreases in average and inferior RNFL thicknesses with longer ethambutol administration periods, and the decreases in nasal, superior, and temporal RNFL showed a nonsignificant trend. There was no significant relationship between RNFL thicknesses and the ethambutol administration period in the linear regression analysis of all studies or female-dominant studies (Figure 5; Table S3). The inferior RNFL thickness showed a significant increase during ethambutol administration in Korean studies, and those of other subsectors did not show a significant decrease. In addition, the results of linear regression analyzing RNFL thicknesses and the administration period did not reveal significant changes in Indian studies, as well as in studies of both mean age groups (Figure S3; Table S3).

### 3.6. GCIPL Changes After the Ethambutol Administration

**Figure 6:**
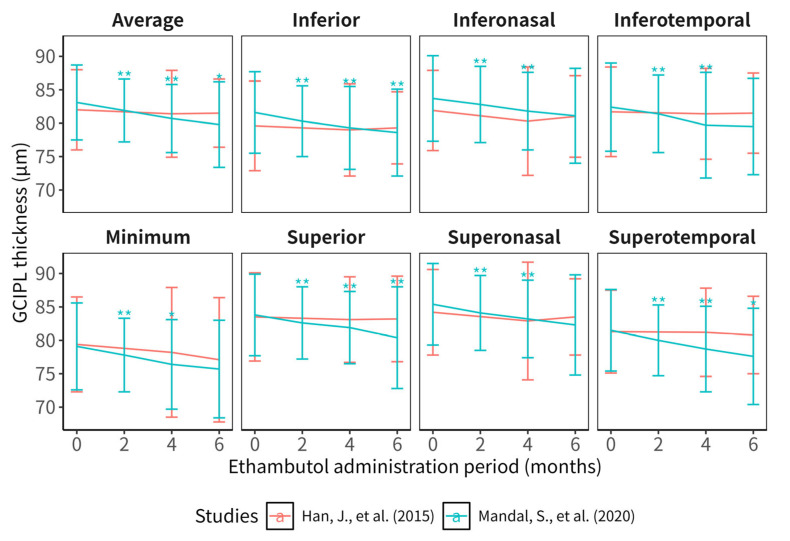
GCIPL thicknesses measured in studies included in this systematic review. The lines connect the means of the GCIPL thicknesses, and error bars refer to the standard deviations reported by the 2 studies eligible for the systematic review [29,33]. The results of tests comparing baseline GCIPL thicknesses and those after a period of ethambutol administration are shown as asterisks: * and ** refer to *p*-values of less than 0.05 and 0.01, respectively. Colors of the lines, error bars, and asterisks refer to different studies. GCIPL = ganglion cell layer and inner plexiform layer.

Since only two studies eligible for the systematic review reported GCIPL thicknesses, we focused on describing the results of GCIPL changes after the ethambutol administration rather than meta-analysis (Figure 6 and Table 4). Mandal and colleagues [33] reported significant decreases in GCIPL in all subsectors 2 and 4 months after the start of the ethambutol administration, and the decreases in average, superior, inferior, and superotemporal GCIPL were also significant in month 6. However, Han and colleagues [29] did not find significant changes in GCIPL in any subsectors in months 4 and 6. The male proportions were 0.720 and 0.528 in studies conducted by Mandal and Han and their colleagues, respectively.

## 4. Discussion

The meta-analysis combining data after the longest periods of ethambutol administration suggested that the effect of ethambutol on RNFL is insignificant overall. However, meta-regression and subgroup analysis revealed heterogeneity of the effect.

Meta-analysis synthesizing studies conducted by different OCT devices showed a significant difference, and the decrease in superior RNFL was more than the test–retest variability of RNFL thickness measured by OCT in previous studies (4.16 to 5.61 μm) [38,39,40,41] in the results synthesizing research conducted by Cirrus (Carl Zeiss Meditec, Dublin, CA, USA). Therefore, differences in RNFL thickness may result from differences in segmentation algorithms, axial resolutions, and standard databases across OCT devices.

Subgroup analysis also showed different effects of ethambutol in patients from different countries, and the thickness changes in superior RNFL in Turkey and inferior RNFL in India were higher than the test–retest variability. This may imply the role of genetic heterogeneity in the development of optic nerve damage induced by ethambutol. Several genes have been found to be related to EON. Zhang and colleagues found optic atrophy-1 (*OPA1*) gene mutation and Leber hereditary optic neuropathy (LHON)–mitochondrial DNA mutation in 46.8% of EON patients [42]. *OPA1* gene mutation was also found in an Indian patient with EON [43]. Since RNFL decrease is a subclinical damage caused by ethambutol, the difference in the decrease may be associated with the distribution of these genes.

In this meta-analysis, the data suggested that sex was related to changes in RNFL thickness caused by ethambutol. There were significant decreases in RNFL thickness in male-dominant studies, which exceeded the test–retest variability of RNFL thickness shown before, while the changes were insignificant in female-dominant studies. Additionally, male-dominant studies showed a significantly negative correlation between RNFL thicknesses and periods of the ethambutol administration. There were only two studies reporting GCIPL changes, and the study involving a higher proportion of males showed a significant decrease in GCIPL thicknesses, while the change in the other study was insignificant. This result was consistent with a retrospective study conducted by Guo and colleagues [18] which observed EON patients for 12 months and found a slower RNFL thinning in female patents compared to males. In addition, another retrospective study revealed better recovery in female EON patients [44]. Therefore, the data in this systematic review suggested higher risks of these changes in male patients treated with ethambutol, and a regular OCT test may be useful in the early diagnosis of EON and necessary for patients treated with ethambutol, especially for male patients.

The gender bias in ethambutol-induced ocular damage may be associated with the pathogenesis, in which ethambutol-induced mitochondrial dysfunction may play a role. Retinal nerve fibers are unmyelinated before they form optic nerves, and this process allows them to maintain the transparency of the retina, but it leads to higher energy demands and vulnerability to oxidative stress in these fibers [45], thereby contributing to EON. LHON is the mitochondrial optic neuropathy that has been studied most thoroughly, and the male–female ratio was approximately 3:1 [46]. The gender bias in LHON was considered to be associated with estrogens and exposure to tobacco and alcohol [47,48,49]. Therefore, these factors may also lead to a higher risk of ethambutol-induced ocular damage in males.

The association with male predominance may also be influenced by confounding factors. First, the prevalence of smoking is higher in males worldwide [50], including India [51], Turkey [52], and Korea [53], where most included studies were held. Smoking has been reported as an independent risk factor for EON [2] and may also cause subclinical damage. Second, research conducted in India, Turkey, and Indonesia tended to recruit patients with a higher male proportion than those conducted in Korea and Brazil. Therefore, regional variation may also contribute to the gender difference. Third, males were at a higher risk of tuberculosis infection and had a more severe form of the disease [54,55] and lower adherence to anti-tuberculosis treatment [56,57]. Therefore, the RNFL decrease in male patients may be associated with the higher levels of inflammation caused by tuberculosis and substandard treatment.

Previous studies have reported numerous factors correlated with EON, including age, ethambutol dosage, treatment period, smoking, hypertension, diabetes, and renal diseases [2,4,6,7]. However, since studies eligible for the meta-analysis did not report information about smoking, hypertension, diabetes, and renal diseases, and patients included in these studies were treated with ethambutol of similar dosage, the association between these factors and retinal layer thicknesses was not analyzed in this study.

This study did not find a significant association between mean ages and RNFL thickness changes, except nasal RNFL. A possible reason is that a significantly increased risk of ethambutol-induced ocular damage was observed in patients aged more than 40 years, especially in those more than 65 years [58]. Therefore, the limited range of mean ages reported by the included studies (28.1 to 46.4 years, except the study by Mane et al. [31]) may not be wide enough to reveal the association.

Our study has several limitations. First, this is a meta-analysis synthesizing data from observational studies. Therefore, selective and confounding biases are inevitable. For example, several studies showed the ocular toxicity of isoniazid [59,60] and the neuroprotective effects of rifampicin [61], which were also involved in these studies and may lead to the misestimation of the changes caused by ethambutol. Second, ethambutol has been proven to be effective in the treatment of tuberculosis for a long time, and OCT is a relatively novel device to detect the retinal layer thicknesses. Therefore, control groups without the administration of ethambutol were not included due to ethical reasons, and these studies were self-controlled. Since tuberculosis may also affect the ocular region [62], the changes in retinal layer thicknesses may be related to subclinical damage induced by tuberculosis. Third, there were only 14 studies eligible for the meta-analysis due to the limited availability of literature sources. Therefore, the small number of studies may not be enough to detect the RNFL thickness changes caused by ethambutol and related factors. The limited data also led to the absence of meta-analyses focusing on GCIPL thickness changes. Fourth, there may be a lack of power to detect RNFL thickness changes in the statistical analysis. Since most participants enrolled in these longitudinal studies underwent detection of RNFL thicknesses before and after the ethambutol administration, paired *t*-tests were used in these studies. Paired *t*-test was able to increase the power to detect the RNFL thickness changes since it reduced the individual differences that are independent of the changes caused by ethambutol. Therefore, a meta-analysis of paired *t*-tests was more appropriate for this study. However, SDs of RNFL changes or the correlation coefficients between RNFL before and after the ethambutol administration were needed in the calculation (Appendix A), and neither of them were reported in these studies. For this reason, we calculated the SMDs through the statistical methods typically used to synthesize unpaired data, which may reduce the power to detect the RNFL thickness changes. Fifth, although this study illustrated the RNFL changes in patients treated with ethambutol, the relationship between the decrease in RNFL and the risk of EON remains unclear. Therefore, further studies are needed to clarify the diagnostic value of OCT in subclinical EON.

## 5. Conclusions

In summary, the results of our meta-analysis suggest that ethambutol administration does not cause significant RNFL changes generally in patients with mycobacterial infection. However, RNFL thickness is reduced in male patients and patients in some countries after ethambutol administration. Further studies are needed to explore the relationship between ethambutol dosages, systemic diseases, smoking, and the changes in RNFL thickness, and the predictive value of RNFL in the development of EON in the future. In addition, we need more research to observe the thickness changes in other retinal layers in patients with ethambutol administration and without EON.

## Figures and Tables

**Table 1:** Characteristics of studies included in this meta-analysis.

Studies	Countries	Male Proportion	Mean Age	Dose (mg/kg)	Devices	Sample Sizes *	Items	Time Points of Follow-Up ^†^ (months)
Chung, 2012 [37]	Korea	0.400	39.3	15–19	S	20	RNFL	0, 1, 2, 3, 4, 5
Dialika, 2015 [25]	Indonesia	0.724	37.0	16.44	S	58	RNFL	0, 2
Findik, 2019 [35]	Turkey	NK	40.3	15	C	28	RNFL	0, 2
Gümüş, 2015 [28]	Turkey	0.750	38.0	15	C	40	RNFL	0, 2
Han, 2015 [29]	Korea	0.528	46.4	15–20	C	72	GCIPL, RNFL	0, 4, 6
Jin, 2019 [30]	Korea	0.464	45.5	14.72	C	168	RNFL	0, 1, 2, 3, 4, 5, 6, 7, 8
Kambella, 2025 [34]	India	0.771	48.9	15	P	192	RNFL	0, 2, 4, 6
Kim, 2015 [11]	Korea	0.452	40.2	15–19	S	62	RNFL	0, 1, 2, 3, 4, 5
Mandal, 2020 [33]	India	0.720	36.4	17.5	C	100	GCIPL, RNFL	0, 2, 4, 6
Mane, 2022 [31]	India	0.489	6.5	20	NK	126	RNFL	0, 2, 6
Menon, 2009 [32]	India	0.558	28.1	15–20	OCT-3	104	RNFL	0, 3
Pavan Taffner, 2018 [36]	Brazil	0.500	43.5	NK	C	52	RNFL	0, 2
Sarkar, 2025 [26]	India	0.545	34.5	NK	NK	44	RNFL	0, 2, 4
Tevaraj, 2017 [27]	Malaysia	0.611	40.0	15	H	72	RNFL	0, 3

* The sample sizes refer to the number of eyes detected in the studies. ^†^ The time point 0 refers to the time before the administration of ethambutol, and other time points refer to the intervals between the beginning of the ethambutol administration and OCT tests (in months). NK, not known; OCT = optical coherence tomography; RNFL, retinal nerve fiber layer; GCIPL, ganglion cell layer and inner plexiform layer; S, Stratus (Carl Zeiss Meditec, Dublin, CA, USA); C, Cirrus (Carl Zeiss Meditec, Dublin, CA, USA); OCT-3, Model 3000 OCT-3 (Carl Zeiss Meditec, Dublin, CA, USA); H, Spectralis (Heidelberg Engineering, Heidelberg, Germany); P, PRIMUS 200 (Carl Zeiss Suzhou Co. Ltd., Suzhou, China).

**Table 2:** The meta-regression results of the RNFL thicknesses.

Subsectors	Male Proportion			Mean Age			Treatment Period		
	Slope (Mean ± SE)	Z	*p*	Slope (Mean ± SE)	Z	*p*	Slope (Mean ± SE)	Z	*p*
Average	−34.51 ± 8.73	−3.95	**<0.001**	−0.12 ± 0.16	−0.77	0.438	−0.47 ± 0.89	−0.53	0.599
Superior	−35.46 ± 13.5	−2.63	**0.009**	−0.34 ± 0.14	−2.39	**0.017**	−0.14 ± 1.02	−0.14	0.887
Inferior	−34.96 ± 10.25	−3.41	**0.001**	−0.04 ± 0.18	−0.25	0.806	−0.42 ± 0.95	−0.44	0.659
Nasal	−24.05 ± 11.55	−2.08	**0.037**	−0.38 ± 0.12	−3.14	**0.002**	−0.22 ± 0.83	−0.27	0.789
Temporal	−17.08 ± 10.25	−1.67	0.096	−0.07 ± 0.13	−0.52	0.603	−0.89 ± 0.68	−1.30	0.193

*p*-values of less than 0.05 are shown in bold. RNFL = retinal nerve fiber layer; SE = standard error.

**Table 3:** Subgroup analysis of RNFL thicknesses.

Subsectors	Subgroups	Levels	*n* of Studies	Patients Before/After Treatment	Weight (%)	RNFL Difference (μm)	Within Subgroups		Among Subgroups	
							Z	*p*	Chi Square	*p*
Average	All		12	1022/836	100.0	−2.43 [−5.77, 0.91]	−1.42	0.154		
	Country	Korea	4	322/168	32.1	1.46 [−4.24, 7.17]	0.50	0.615	3.03	0.387
		India	4	522/490	34.9	−5.79 [−12.49, 0.91]	−1.69	0.091		
		Turkey	2	68/68	17.0	−3.18 [−5.67, −0.7]	−2.51	**0.012**		
		Others	2	110/110	16.0	−2.39 [−6.33, 1.54]	−1.19	0.234		
	Device	C	6	460/306	58.9	−3.88 [−6.81, −0.95]	−2.59	**0.010**	4.32	0.115
		S	3	140/140	29.9	2.36 [−5.27, 9.98]	0.61	0.545		
		Others	1	104/104	11.2	−0.3 [−2.88, 2.28]	−0.23	0.820		
	Dominant sex	Female	7	604/418	63.0	0.89 [−1.95, 3.73]	0.61	0.539	8.79	**0.003**
		Male	4	390/390	37.0	−7.71 [−12.63, −2.78]	−3.07	**0.002**		
	Mean age	Less than 40	6	448/416	50.7	−2.51 [−5.78, 0.75]	−1.51	0.131	0.00	0.956
		40 or more	6	574/420	49.3	−2.31 [−8.78, 4.16]	−0.70	0.484		
Superior	All		14	1138/952	100.0	−3.6 [−7.21, 0.02]	−1.95	0.051		
	Country	Korea	4	322/168	27.5	−1.85 [−5.05, 1.35]	−1.13	0.256	3.11	0.374
		India	5	566/534	37.0	−5.5 [−14.75, 3.74]	−1.17	0.243		
		Turkey	2	68/68	14.0	−6.1 [−10.46, −1.73]	−2.74	**0.006**		
		Others	3	182/182	21.4	−1.34 [−6.42, 3.74]	−0.52	0.604		
	Device	C	6	460/306	50.6	−6.26 [−10.58, −1.94]	−2.84	**0.005**	4.45	0.108
		S	3	140/140	26.2	−1.97 [−6.47, 2.54]	−0.85	0.393		
		Others	2	176/176	23.2	−0.07 [−3.97, 3.84]	−0.03	0.974		
	Dominant sex	Female	9	720/534	68.9	−0.4 [−3.95, 3.16]	−0.22	0.826	11.55	**0.001**
		Male	4	390/390	31.1	−10 [−14.24, −5.75]	−4.62	**<0.001**		
	Mean age	Less than 40	7	492/460	49.1	−3.68 [−10.38, 3.02]	−1.08	0.282	0.00	0.979
		40 or more	7	646/492	50.9	−3.57 [−7.72, 0.58]	−1.69	0.092		
Inferior	All		14	1138/952	100.0	−2.77 [−6.25, 0.72]	−1.56	0.119		
	Country	Korea	4	322/168	28.0	2.62 [−0.66, 5.91]	1.57	0.118	11.27	**0.010**
		India	5	566/534	36.9	−6.97 [−12.92, −1.02]	−2.29	**0.022**		
		Turkey	2	68/68	14.2	−4.74 [−9.44, −0.05]	−1.98	**0.048**		
		Others	3	182/182	20.9	0.2 [−3.45, 3.85]	0.11	0.916		
	Device	C	6	460/306	50.1	−4.18 [−7.21, −1.16]	−2.71	**0.007**	8.88	**0.012**
		S	3	140/140	26.8	3.17 [−1.21, 7.54]	1.42	0.156		
		Others	2	176/176	23.0	0.88 [−2.55, 4.31]	0.50	0.615		
	Dominant sex	Female	9	720/534	68.0	0.36 [−2, 2.72]	0.30	0.766	6.87	**0.009**
		Male	4	390/390	32.0	−8.15 [−14.06, −2.24]	−2.70	**0.007**		
	Mean age	Less than 40	7	492/460	48.5	−3.1 [−6.34, 0.15]	−1.87	0.061	0.03	0.868
		40 or more	7	646/492	51.5	−2.5 [−8.69, 3.69]	−0.79	0.428		
Nasal	All		14	1138/952	100.0	−1.78 [−4.78, 1.22]	−1.16	0.245		
	Country	Korea	4	322/168	27.2	−0.53 [−2.45, 1.39]	−0.54	0.588	0.37	0.947
		India	5	566/534	36.3	−2.98 [−11.16, 5.19]	−0.72	0.474		
		Turkey	2	68/68	14.3	−1.07 [−4.88, 2.75]	−0.55	0.584		
		Others	3	182/182	22.2	−0.66 [−4.38, 3.05]	−0.35	0.727		
	Device	C	6	460/306	49.8	−2.54 [−4.32, −0.76]	−2.80	**0.005**	10.17	**0.006**
		S	3	140/140	26.1	0.51 [−1.48, 2.5]	0.50	0.614		
		Others	2	176/176	24.1	2.02 [−0.41, 4.45]	1.63	0.103		
	Dominant sex	Female	9	720/534	68.7	0.08 [−2.92, 3.08]	0.05	0.958	5.20	**0.023**
		Male	4	390/390	31.3	−6.12 [−10.52, −1.71]	−2.72	**0.006**		
Nasal	Mean age	Less than 40	7	492/460	45.1	−1.25 [−7.25, 4.75]	−0.41	0.683	0.07	0.786
		40 or more	7	646/492	54.9	−2.23 [−5.92, 1.46]	−1.18	0.237		
Temporal	All		14	1138/952	100.0	−1.79 [−4.33, 0.76]	−1.38	0.169		
	Country	Korea	4	322/168	24.7	−0.16 [−4.39, 4.07]	−0.07	0.941	4.81	0.186
		India	5	566/534	38.6	−4.69 [−8.22, −1.16]	−2.60	**0.009**		
		Turkey	2	68/68	15.4	−0.87 [−3.44, 1.7]	−0.66	0.506		
		Others	3	182/182	21.4	1.32 [−4.03, 6.67]	0.48	0.629		
	Device	C	6	460/306	52.2	−2.49 [−5.05, 0.07]	−1.90	0.057	2.23	0.327
		S	3	140/140	25.1	1.01 [−3.26, 5.29]	0.47	0.642		
		Others	2	176/176	22.7	1.07 [−7.6, 9.74]	0.24	0.809		
	Dominant sex	Female	9	720/534	67.7	−0.13 [−2.7, 2.45]	−0.10	0.924	3.18	0.075
		Male	4	390/390	32.3	−4.98 [−9.66, −0.3]	−2.09	**0.037**		
	Mean age	Less than 40	7	492/460	49.6	−2.29 [−4.4, −0.17]	−2.12	**0.034**	0.14	0.712
		40 or more	7	646/492	50.4	−1.31 [−6.06, 3.44]	−0.54	0.589		

RNFL thicknesses are shown as mean [95% confidence interval]. *p*-values of less than 0.05 are shown in bold. RNFL, retinal nerve fiber layer; S, Stratus (Carl Zeiss Meditec, Dublin, CA, USA); C, Cirrus (Carl Zeiss Meditec, Dublin, CA, USA).

**Table 4:** The changes in GCIPL in tuberculosis patients treated with ethambutol.

Studies	Countries	*n*	Male Proportion	Subsectors	Month 0	Month 2		Month 4		Month 6	
						Mean ± SD	*p*	Mean ± SD	*p*	Mean ± SD	*p*
Han, 2015 [29]	Korea	72	0.528	Average	82 ± 6			81.4 ± 6.5	0.767	81.5 ± 5.1	0.862
				Minimum	79.4 ± 7.1			78.2 ± 9.7	0.424	77.1 ± 9.3	0.264
				Superonasal	84.2 ± 6.4			82.9 ± 8.8	0.838	83.5 ± 5.7	0.981
				Superior	83.5 ± 6.6			83.1 ± 6.4	0.537	83.2 ± 6.4	0.545
				Superotemporal	81.3 ± 6.2			81.2 ± 6.6	0.736	80.8 ± 5.8	0.845
				Inferonasal	81.9 ± 6			80.3 ± 8.1	0.305	81 ± 6.1	0.260
				Inferior	79.6 ± 6.7			79 ± 6.9	0.939	79.3 ± 5.4	0.954
				Inferotemporal	81.7 ± 6.7			81.4 ± 6.8	0.945	81.5 ± 6	0.538
Mandal, 2020 [33]	India	100	0.720	Average	83.1 ± 5.6	81.9 ± 4.7	**0.001**	80.7 ± 5.1	**0.001**	79.8 ± 6.4	**0.011**
				Minimum	79.1 ± 6.5	77.8 ± 5.5	**0.001**	76.4 ± 6.7	**0.025**	75.7 ± 7.3	0.822
				Superior	83.8 ± 6.1	82.6 ± 5.4	**0.001**	81.9 ± 5.4	**0.002**	80.4 ± 7.6	**0.002**
				Superonasal	85.4 ± 6.1	84.1 ± 5.6	**0.001**	83.2 ± 5.8	**0.001**	82.3 ± 7.5	0.058
				Inferonasal	83.7 ± 6.4	82.8 ± 5.7	**0.002**	81.8 ± 5.8	**0.001**	81.1 ± 7.1	0.099
				Inferior	81.6 ± 6.1	80.3 ± 5.3	**0.001**	79.3 ± 6.2	**0.003**	78.6 ± 6.5	**0.005**
				Inferotemporal	82.4 ± 6.6	81.4 ± 5.8	**0.002**	79.7 ± 7.9	**0.004**	79.5 ± 7.2	0.733
				Superotemporal	81.5 ± 6.1	80 ± 5.3	**0.001**	78.7 ± 6.4	**0.002**	77.6 ± 7.2	**0.021**

*p*-values are obtained by comparing baseline (month 0) and those after ethambutol administration. *p*-values of less than 0.05 are shown in bold. GCIPL = ganglion cell layer and inner plexiform layer; SD = standard deviation.

## Data Availability

All original data in this study are published and cited in the article.

## Supplementary Materials

The following supporting information can be downloaded at https://www.mdpi.com/article/10.3390/jcm15031230/s1, Table S1. PRISMA-COSMIN 2024 checklist. Table S2. Risk of bias evaluated using ROBINS-I criteria. Table S3. The results of meta-analysis focusing on the same period of ethambutol administration. Figure S1. Meta-regression of RNFL thicknesses in subsectors. Figure S2. Subgroup analysis of RNFL thicknesses. Figure S3. Meta-regression of RNFL thicknesses in studies with different mean ages and countries. Figure S4. RNFL thicknesses after the same period of the administration of ethambutol.

## Abbreviations

The following abbreviations are used in this manuscript:

EON	Ethambutol-induced optic neuropathy
RNFL	Retinal nerve fiber layer
GCIPL	Ganglion cell layer and inner plexiform layer
OCT	Optical coherence tomography
PRISMA-COSMIN	Meta-Analyses COnsensus-based Standards for the selection of health Measurement Instruments
OMI	Outcome measurement instrument
SD	Standard deviation
SMD	Standardized mean difference
CI	Confidence interval
LHON	Leber’s hereditary optic neuropathy
*OPA1*	Optic atrophy-1
V	Variance
SE	Standard error
MD	Mean difference

## Appendix A

In the synthesis of data, the first step is to calculate the value, variance (V), and standard error (SE) of the mean difference (MD) in each study, all of which are the basis for synthesizing the results.

For meta-analysis of unpaired *t*-tests, these statistical magnitudes can be calculated by (A1) to (A3), as shown below:

(A1) $$MD={Mean}_{post}-{Mean}_{pre}$$

(A2) $$V=\frac{{SD}_{pre}^{2}+{SD}_{post}^{2}}{N}$$

(A3) $$SE=\sqrt{V}$$

where

${Mean}_{pre}$	Mean RNFL thickness before the ethambutol administration;
${Mean}_{post}$	Mean RNFL thickness after the ethambutol administration;
${SD}_{pre}$	The standard deviation of RNFL thickness before the ethambutol administration;
${SD}_{post}$	The standard deviation of RNFL thickness after the ethambutol administration;
N	The number of eyes.

Since these values were reported in all included studies, a meta-analysis of unpaired *t*-tests could be conducted.

In a meta-analysis of paired *t*-tests, the MD and SE can also be calculated using (A1) and (A3). However, the V should be calculated through any equations shown below:

(A4) $$V=\frac{{SD}_{post-pre}^{2}}{N}$$

(A5) $$V=\frac{{SD}_{pre}^{2}+{SD}_{post}^{2}-2\cdot r\cdot {SD}_{pre}\cdot {SD}_{post}}{N}$$

where

${SD}_{post-pre}$	The standard deviation of RNFL thickness changes before and after the ethambutol administration;
r	The correlation coefficient of RNFL thicknesses before and after the ethambutol administration.

Unfortunately, none of the included studies reported ${SD}_{post-pre}$ or r. Therefore, a meta-analysis of paired *t*-test could not be conducted.
