# Supplementary material for: Longitudinal Observation by Optical Coherence Tomography in Patients Treated with Ethambutol: A Systematic Review and Meta-Analysis
Source: J Clin Med. 2026 Feb 4;15(3):1230. doi: 10.3390/jcm15031230 (PMC12898144; doi:10.3390/jcm15031230)
Supplement: Supplementary file 1 [file jcm-15-01230-s001.zip › jcm-4100878-supplementary.pdf]

Table S1. PRISMA-COSMIN 2024 checklist

Section and Topic	#	Checklist item[a]	Location
TITLE			
Title	1	Identify the report as a systematic review and include as applicable the following (in any order): outcome domain of interest, population of interest, name/type of OMIs of interest, and measurement properties of interest.	Page 1
ABSTRACT			
OPEN SCIENCE			
Funding[b]	2.2	Specify the primary source of funding for the review.	Page 16
Registration	2.3	Provide the register name and registration number.	Page 2
BACKGROUND			
Objectives	2.4	Provide an explicit statement of the main objective(s) or question(s) the review addresses.	Page 2
METHODS			
Eligibility criteria	2.5	Specify the inclusion and exclusion criteria for the review.	Page 2-3
Information sources	2.6	Specify the information sources (e.g., databases, registers) used to identify studies and the date when each was last searched.	Page 3
Risk of bias	2.7	Specify the methods used to assess risk of bias in the included studies.	Page 3
Measurement properties	2.8	Specify the methods used to rate the results of a measurement property.	Page 3
Synthesis methods	2.9	Specify the methods used to present and synthesize results.	Page 3-4
RESULTS			
Included studies	2.10	Give the total number of included OMIs and study reports.	Page 4
Synthesis of results	2.11	Present the syntheses of results of OMIs, indicating the certainty of the evidence.	Page 4-13
DISCUSSION			
Limitations of evidence	2.12	Provide a brief summary of the limitations of the evidence included in the review (e.g., study risk of bias, inconsistency, and imprecision).	Page 15
Interpretation	2.13	Provide a general interpretation of the results and important implications.	Page 15-16
PLAIN LANGUAGE SUMMARY			
Plain language summary	3	If allowed by the journal, provide a plain language summary with background information and key findings.	-
OPEN SCIENCE			
Registration and protocol	4a	Provide registration information for the review, including register name and	Page 2

Section and Topic	#	Checklist item[a]	Location
		registration number, or state that the review was not registered.	
	4b	Indicate where the review protocol can be accessed, or state that a protocol was not prepared.	Page 2
	4c	Describe and explain any amendments to information provided at registration or in the protocol.	Page 2
Support	5	Describe sources of financial or non-financial support for the review, and the role of the funders in the review.	Page 16
Competing interests	6	Declare any competing interests of review authors.	Page 16
Availability of data, code, and other materials	7	Report which of the following are publicly available and where they can be found: template data collection forms; data extracted from included studies; data used for all analyses; analytic code; any other materials used in the review.	Page 16
INTRODUCTION			
Rationale	8	Describe the rationale for the review in the context of existing knowledge.	Page 2
Objectives	9	Provide an explicit statement of the objective(s) or question(s) the review addresses and include as applicable the following (in any order): outcome domain of interest, population of interest, name/type of OMIs of interest, and measurement properties of interest.	Page 2
METHODS			
Followed guidelines	10	Specify, with references, the methodology and/or guidelines used to conduct the systematic review.	Page 2
Eligibility criteria	11	Specify the inclusion and exclusion criteria for the review.	Page 2-3
Information sources	12	Specify all databases, registers, preprint servers, websites, organizations, reference lists and other sources searched or consulted to identify studies. Specify the date when each source was last searched or consulted.	Page 3
Search strategy	13	Present the full search strategies for all databases, registers, and websites, including any filters and limits used.	Page 3
Selection process	14	Specify the methods used to decide whether a study met the inclusion criteria of the review, e.g., including how many reviewers screened each record and each report retrieved, whether they worked independently, and if applicable, details of automation tools/AI used in the process.	Page 3
Data collection process	15	Specify the methods used to collect data from reports, e.g., including how many reviewers collected data from each report, whether they worked independently, any processes for obtaining or confirming data from study investigators, and if applicable, details of	Page 3

		automation tools/AI used in the process.	
Data items	16	List and define which data were extracted (e.g., characteristics of study populations and OMIs, measurement properties' results, and aspects of feasibility and interpretability). Describe methods used to deal with any missing or unclear information.	Page 3
Study risk of bias assessment	17	Specify the methods used to assess risk of bias in the included studies, e.g., including details of the tool(s) used, how many reviewers assessed each study and whether they worked independently, and if applicable, details of automation tools/AI used in the process.	Page 3
Measurement properties	18	Specify the methods used to rate the results of a measurement property for each individual study and for the summarized or pooled results, e.g., including how many reviewers rated each study and whether they worked independently.	Page 3
Synthesis methods	19a	Describe the processes used to decide which studies were eligible for each synthesis.	Page 3-4
	19b	Describe any methods used to synthesize results.	Page 3-4
	19c	If applicable, describe any methods used to explore possible causes of inconsistency among study results (e.g., subgroup analysis).	Page 3-4
	19d	If applicable, describe any sensitivity analyses conducted to assess robustness of the synthesized results.	-
Certainty assessment	20	Describe any methods used to assess certainty (or confidence) in the body of evidence.	Page 3
Formulating recommendations	21	If appropriate, describe any methods used to formulate recommendations regarding the suitability of OMIs for a particular use.	-
RESULTS			
Study selection	22a	Describe the results of the search and selection process, from the number of records identified in the search to the number of study reports included in the review, ideally using a flow diagram. If applicable, also report the final number of OMIs included and the number of study reports relevant to each OMI. [T]	Page 4
	22b	Cite study reports that might appear to meet the inclusion criteria, but which were excluded, and explain why they were excluded.	Page 4
OMI characteristics	23a	Present characteristics of each included OMI, with appropriate references. [T]	Page 5
	23b	If applicable, present interpretability aspects for each included OMI. [T]	-
	23c	If applicable, present feasibility aspects for each included OMI. [T]	-
Study characteristics	24	Cite each included study report evaluating one or more measurement properties and present its characteristics. [T]	Page 6

Section and Topic	#	Checklist item[a]	Location
Risk of bias in studies	25	Present assessments of risk of bias for each included study. [T]	Table S2
Results of individual studies	26	For all measurement properties, present for each study: (a) the reported result and (b) the rating against quality criteria, ideally using structured tables or plots. [T]	Page 5
Results of syntheses	27a	Present results of all syntheses conducted. For each measurement property of an OMI, present: (a) the summarized or pooled result and (b) the overall rating against quality criteria. [T]	Page 6-7
	27b	If applicable, present results of all investigations of possible causes of inconsistency among study results.	Page 8-13
	27c	If applicable, present results of all sensitivity analyses conducted to assess the robustness of the synthesized results.	-
Certainty of evidence	28	Present assessments of certainty (or confidence) in the body of evidence for each measurement property of an OMI assessed. [T]	-
Recommendations	29	If appropriate, make recommendations for suitable OMIs for a particular use.	-
DISCUSSION			
Discussion	30a	Provide a general interpretation of the results in the context of other evidence.	Page 14-15
	30b	Discuss any limitations of the evidence included in the review.	Page 15
	30c	Discuss any limitations of the review processes used.	Page 15-17
	30d	Discuss implications of the results for practice, policy, and future research.	Page 16-17

Table S2. Risk of bias evaluated using ROBINS-I criteria

Reference (first author and year)	Confounding bias	Selection of participants	Classification of interventions	Deviation from interventions	Missing data	Measurement of outcomes	Results reporting
Chung 2012	Moderate	Low	Low	Low	Low	Low	Moderate
Dialika 2015	Moderate	Low	Low	Low	Low	Low	Moderate
Findik 2019	Moderate	Low	Low	Low	Low	Low	Moderate
Gümüş 2015	Moderate	Low	Low	Low	Low	Low	Moderate
Han 2015	Moderate	Low	Low	Low	Low	Low	Moderate
Jin 2019	Moderate	Low	Low	Low	Moderate	Low	Moderate
Kambella 2025	Moderate	Low	Low	Low	Low	Low	Moderate
Kim 2015	Moderate	Low	Low	Low	Low	Low	Moderate
Mandal 2020	Moderate	Low	Low	Low	Low	Low	Moderate
Mane 2022	Moderate	Low	Low	Low	Moderate	Low	Moderate
Menon 2009	Moderate	Low	Low	Low	Low	Low	Moderate
Pavan Taffner 2018	Moderate	Low	Low	Low	Low	Low	Moderate
Sarkar 2025	Moderate	Low	Low	Low	Low	Low	Moderate
Tevaraj 2017	Moderate	Low	Low	Low	Low	Low	Moderate

Table S3. The results of meta-analysis focusing on the same period of ethambutol administration

Groups	Positions	Treatment periods (months)	RNFL differences	Z	P	Linear regression		
						Slopes	Standard errors	P
All	Average	2	-1.47 [-2.99, 0.06]	-1.88	0.06	-0.90	0.53	0.228
		3	0.64 [-1.62, 2.9]	0.56	0.577			
		4	-1.91 [-5.71, 1.9]	-0.98	0.326			
		6	-4.23 [-10.32, 1.86]	-1.36	0.174			
	Inferior	2	-2.42 [-4.01, -0.83]	-2.99	**0.003**	-0.90	0.75	0.356
		3	1.01 [-1.46, 3.47]	0.80	0.423			
		4	-2.34 [-7, 2.31]	-0.99	0.324			
		6	-4.77 [-11.73, 2.19]	-1.34	0.179			
	Nasal	2	-1.57 [-2.66, -0.47]	-2.80	**0.005**	-0.14	0.59	0.833
		3	-0.64 [-3.17, 1.89]	-0.49	0.622			
		4	-4.01 [-6.64, -1.38]	-2.98	**0.003**			
		6	-1.53 [-7.91, 4.84]	-0.47	0.637			
	Superior	2	-2.77 [-4.64, -0.89]	-2.89	**0.004**	-0.29	0.71	0.727
		3	-0.11 [-2.02, 1.8]	-0.12	0.908			
		4	-4.42 [-7.45, -1.38]	-2.85	**0.004**			
		6	-2.81 [-12.89, 7.27]	-0.55	0.585			
	Temporal	2	-1.25 [-2.36, -0.15]	-2.22	**0.026**	-0.76	0.62	0.342
		3	1.47 [-2.27, 5.22]	0.77	0.44			
		4	-1.81 [-4.93, 1.31]	-1.14	0.256			
		6	-3.24 [-7.86, 1.38]	-1.37	0.17			
Country = India	Average	2	-2.17 [-4.9, 0.57]	-1.55	0.121	-1.72	0.88	0.192
		3	-0.3 [-2.88, 2.28]	-0.23	0.82			
		4	-7.04 [-9.14, -4.94]	-6.57	**< 0.001**			
		6	-7.67 [-16.43, 1.08]	-1.72	0.086			
	Inferior	2	-3.42 [-5.55, -1.29]	-3.15	**0.002**	-1.93	1.07	0.212
		3	-0.48 [-5.33, 4.37]	-0.19	0.846			
		4	-8.29 [-11.12, -5.47]	-5.75	**< 0.001**			
		6	-9.4 [-16.3, -2.51]	-2.67	**0.008**			
	Nasal	2	-2.76 [-5.15, -0.38]	-2.27	**0.023**	-0.39	1.66	0.838
		3	2.63 [-2.27, 7.53]	1.05	0.293			
		4	-7.27 [-9.75, -4.79]	-5.75	**< 0.001**			
		6	-1.96 [-14.61, 10.69]	-0.30	0.761			
	Superior	2	-3.25 [-7.03, 0.53]	-1.69	0.092	-0.84	0.77	0.389
		3	-2.49 [-7, 2.02]	-1.08	0.279			
		4	-7.62 [-10.99, -4.24]	-4.42	**< 0.001**			
		6	-5.79 [-21.96, 10.37]	-0.70	0.482			
	Temporal	2	-1.9 [-3.69, -0.11]	-2.08	**0.037**	-0.91	0.36	0.125
		3	-3.32 [-6.1, -0.54]	-2.34	**0.019**			
		4	-5.54 [-7.33, -3.74]	-6.04	**< 0.001**			
		6	-5.51 [-11.52, 0.49]	-1.80	0.072			
Country = Korea	Average	2	1.26 [-0.83, 3.35]	1.18	0.238	-0.11	0.09	0.337
		3	1.11 [-2.23, 4.44]	0.65	0.515			
		4	0.62 [-1.92, 3.16]	0.48	0.633			
		6	0.85 [-4.69, 6.4]	0.30	0.763			

Groups	Positions	Treatment periods (months)	RNFL differences	Z	P	Linear regression		
						Slopes	Standard errors	P
	Inferior	2	0.9 [-2.29, 4.09]	0.55	0.581	0.34	0.03	**0.009**
		3	1.14 [-3.59, 5.88]	0.47	0.636			
		4	1.4 [-2.05, 4.85]	0.80	0.426			
		6	2.24 [-3.68, 8.16]	0.74	0.459			
	Nasal	2	-0.44 [-2.12, 1.23]	-0.52	0.606	-0.10	0.36	0.807
		3	-2.59 [-4.2, -0.98]	-3.16	**0.002**			
		4	-1.55 [-3.12, 0.02]	-1.93	0.054			
		6	-1.42 [-4.07, 1.23]	-1.05	0.294			
	Superior	2	-0.32 [-3.26, 2.63]	-0.21	0.832	0.49	0.43	0.368
		3	-1.03 [-4.19, 2.12]	-0.64	0.521			
		4	-1.56 [-4.47, 1.36]	-1.05	0.295			
		6	1.5 [-4.44, 7.44]	0.50	0.62			
	Temporal	2	0.35 [-2.1, 2.79]	0.28	0.782	-0.12	0.33	0.755
		3	2.06 [-0.65, 4.77]	1.49	0.136			
		4	1.19 [-1.09, 3.47]	1.03	0.305			
		6	0.37 [-5.34, 6.08]	0.13	0.899			
Dominant sex = Female	Average	2	0.68 [-0.76, 2.11]	0.93	0.355	-0.11	0.03	0.070
		3	0.64 [-1.62, 2.9]	0.56	0.577			
		4	0.62 [-1.92, 3.16]	0.48	0.633			
		6	0.26 [-2.31, 2.83]	0.20	0.845			
	Inferior	2	-1.03 [-3.4, 1.34]	-0.85	0.394	0.14	0.36	0.743
		3	1.01 [-1.46, 3.47]	0.80	0.423			
		4	0.73 [-2.76, 4.23]	0.41	0.681			
		6	-0.01 [-4.92, 4.89]	-0.01	0.996			
	Nasal	2	-0.92 [-2.39, 0.55]	-1.23	0.22	1.08	0.79	0.306
		3	-0.64 [-3.17, 1.89]	-0.49	0.622			
		4	-2.7 [-5.47, 0.06]	-1.92	0.055			
		6	3.57 [-4.73, 11.88]	0.84	0.399			
	Superior	2	-0.75 [-3, 1.49]	-0.66	0.511	1.30	0.91	0.289
		3	-0.11 [-2.02, 1.8]	-0.12	0.908			
		4	-2.6 [-5.67, 0.47]	-1.66	0.097			
		6	4.71 [-2.26, 11.69]	1.32	0.185			
	Temporal	2	0.16 [-1.53, 1.85]	0.18	0.853	-0.19	0.27	0.546
		3	1.47 [-2.27, 5.22]	0.77	0.44			
		4	0.16 [-2.29, 2.61]	0.13	0.899			
		6	-0.15 [-2.75, 2.45]	-0.11	0.909			
Dominant sex = Male	Average	2	-3.14 [-4.56, -1.72]	-4.32	**< 0.001**	-2.21	0.15	**0.044**
		4	-7.04 [-9.14, -4.94]	-6.57	**< 0.001**			
		6	-11.99 [-14.33, -9.66]	-10.06	**< 0.001**			
	Inferior	2	-3.39 [-5.64, -1.15]	-2.97	**0.003**	-2.42	0.07	**0.018**
		4	-8.46 [-11.39, -5.54]	-5.67	**< 0.001**			
		6	-13.07 [-16.75, -9.38]	-6.95	**< 0.001**			
	Nasal	2	-2.48 [-4.2, -0.76]	-2.83	**0.005**	-1.72	0.18	0.068
		4	-6.55 [-8.71, -4.39]	-5.94	**< 0.001**			
		6	-9.35 [-11.52, -7.19]	-8.49	**< 0.001**			

Groups	Positions	Treatment periods (months)	RNFL differences	Z	P	Linear regression		
						Slopes	Standard errors	P
	Superior	2	-4.99 [-8.48, -1.5]	-2.80	**0.005**	-2.04	0.28	0.087
		4	-8.09 [-13.73, -2.46]	-2.81	**0.005**			
		6	-13.14 [-17.74, -8.54]	-5.60	**< 0.001**			
	Temporal	2	-2.33 [-3.89, -0.78]	-2.94	**0.003**	-1.53	0.13	0.053
		4	-5.84 [-7.78, -3.9]	-5.91	**< 0.001**			
		6	-8.47 [-10.53, -6.41]	-8.05	**< 0.001**			
Mean age = 40 or more	Average	2	-0.68 [-2.87, 1.51]	-0.61	0.541	-0.89	0.44	0.179
		3	0.92 [-3.97, 5.81]	0.37	0.713			
		4	-1.78 [-6.47, 2.9]	-0.75	0.456			
		6	-3.51 [-12.88, 5.87]	-0.73	0.464			
	Inferior	2	-1.85 [-4.14, 0.43]	-1.59	0.112	-0.52	0.46	0.375
		3	0.26 [-6.12, 6.64]	0.08	0.935			
		4	-1.94 [-8.37, 4.49]	-0.59	0.554			
		6	-3.17 [-15.54, 9.21]	-0.50	0.616			
	Nasal	2	-1.3 [-2.52, -0.09]	-2.10	**0.035**	-0.57	0.13	0.050
		3	-2.61 [-4.24, -0.98]	-3.14	**0.002**			
		4	-2.9 [-5.61, -0.19]	-2.10	**0.036**			
		6	-3.78 [-9.99, 2.42]	-1.20	0.232			
	Superior	2	-1.54 [-3.45, 0.37]	-1.58	0.114	-0.44	0.39	0.380
		3	-1.37 [-4.73, 1.99]	-0.80	0.423			
		4	-3.92 [-6.17, -1.67]	-3.41	**< 0.001**			
		6	-2.92 [-12.68, 6.83]	-0.59	0.557			
	Temporal	2	-1.4 [-3, 0.2]	-1.72	0.086	-0.64	0.74	0.477
		3	2.2 [-0.7, 5.1]	1.49	0.137			
		4	-1.11 [-5.78, 3.55]	-0.47	0.64			
		6	-2.72 [-9.46, 4.01]	-0.79	0.428			
Mean age = Less than 40	Average	2	-2.4 [-4.84, 0.05]	-1.92	0.055	-0.93	0.61	0.266
		3	0.04 [-2.3, 2.38]	0.03	0.974			
		4	-2.13 [-11.44, 7.18]	-0.45	0.654			
		6	-5.22 [-16.1, 5.65]	-0.94	0.346			
	Inferior	2	-3.14 [-5.74, -0.54]	-2.37	**0.018**	-1.25	0.99	0.337
		3	1.31 [-1.89, 4.52]	0.80	0.422			
		4	-3.05 [-10.58, 4.49]	-0.79	0.428			
		6	-6.51 [-13.04, 0.02]	-1.95	0.051			
	Nasal	2	-2.7 [-5.24, -0.17]	-2.09	**0.037**	0.71	1.75	0.724
		3	1.82 [-0.55, 4.18]	1.51	0.132			
		4	-7.24 [-13.2, -1.29]	-2.38	**0.017**			
		6	2.08 [-18.5, 22.66]	0.20	0.843			
	Superior	2	-4.41 [-7.77, -1.05]	-2.57	**0.01**	-0.09	1.10	0.939
		3	0.36 [-2.29, 3]	0.26	0.791			
		4	-5.88 [-14.05, 2.29]	-1.41	0.158			
		6	-3.03 [-28.8, 22.75]	-0.23	0.818			
	Temporal	2	-0.82 [-2.55, 0.9]	-0.93	0.35	-1.01	0.60	0.234
		3	1.07 [-5.55, 7.7]	0.32	0.751			
		4	-3.15 [-6.87, 0.57]	-1.66	0.097			

Groups	Positions	Treatment periods (months)	RNFL differences	Z	P	Linear regression		
						Slopes	Standard errors	P
		6	-3.87 [-11.98, 4.25]	-0.93	0.35			

This table summarizes the results of meta-analysis focusing on the same period of ethambutol administration, along with the subgroup analysis and the linear regression. The RNFL thickness differences are shown as mean [95 % confidence interval]. P values of less than 0.05 are highlighted in bold. RNFL = retinal nerve fiber layer.

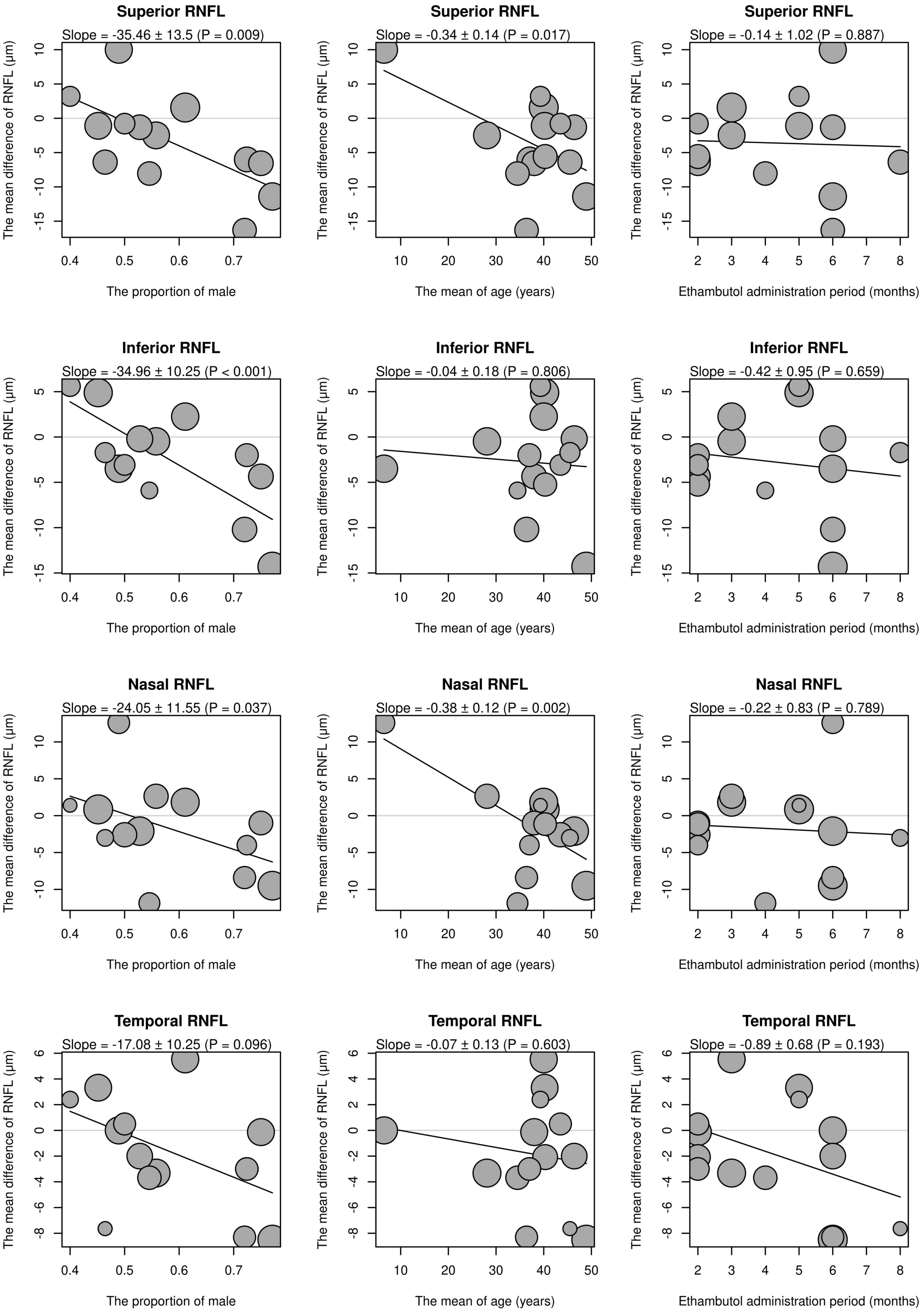

Figure S1. Meta-regression of RNFL thicknesses in subsectors. This figure shows bubble plots obtained in the meta-regression of RNFL thicknesses in subsectors, which visualize the relationship between RNFL thicknesses and the proportion of males, the mean age, as well as the period of the ethambutol administration. The sizes of the bubbles refer to the weights of the studies, and the black lines are obtained in the regression, while the gray lines represent the mean difference of 0. The slopes of the black lines are shown at the top of the bubble plots as estimates ± standard errors, followed by *p*-values.RNFL = retinal nerve fiber layer.

A

i. Country

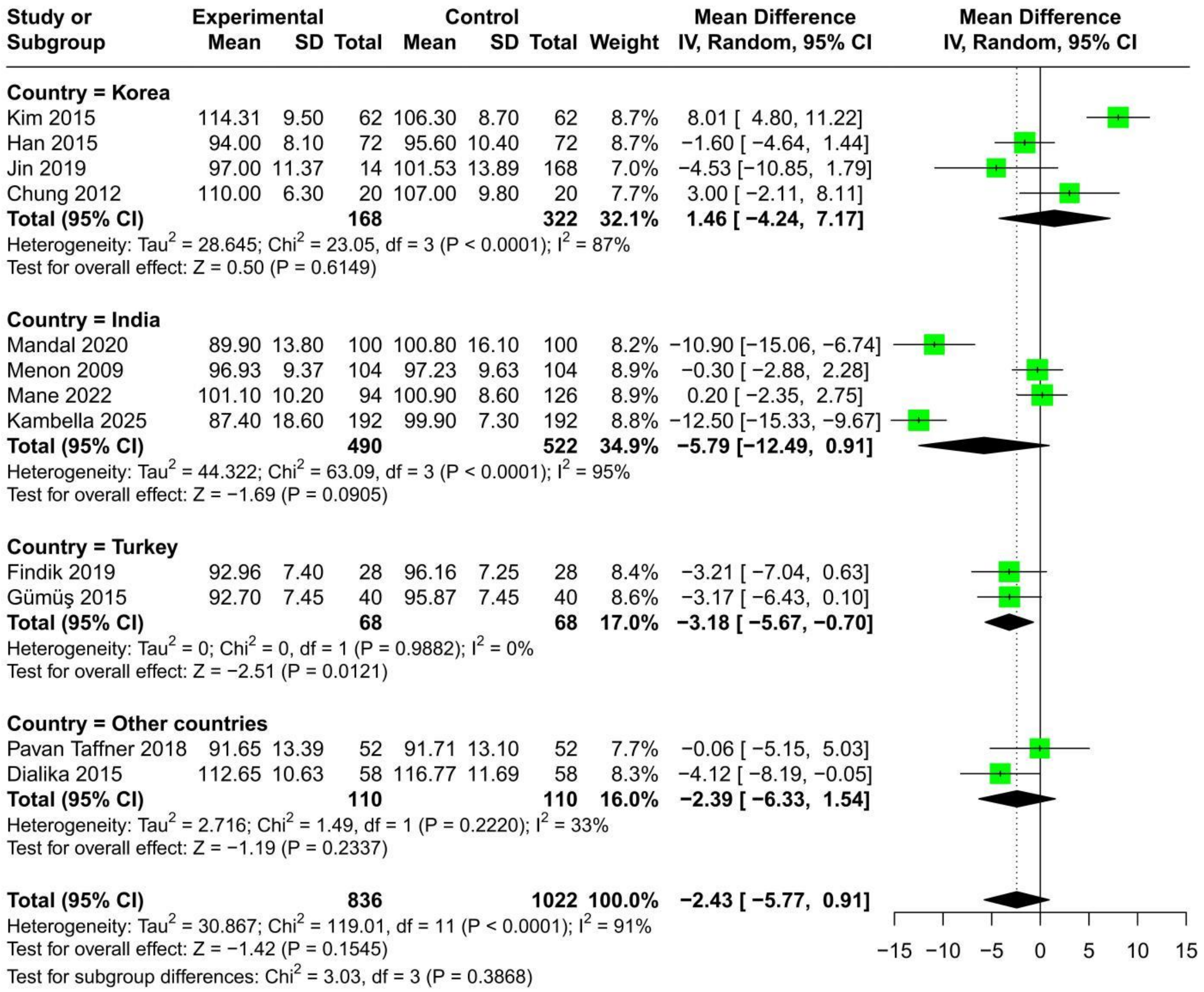

ii. Dominant sex

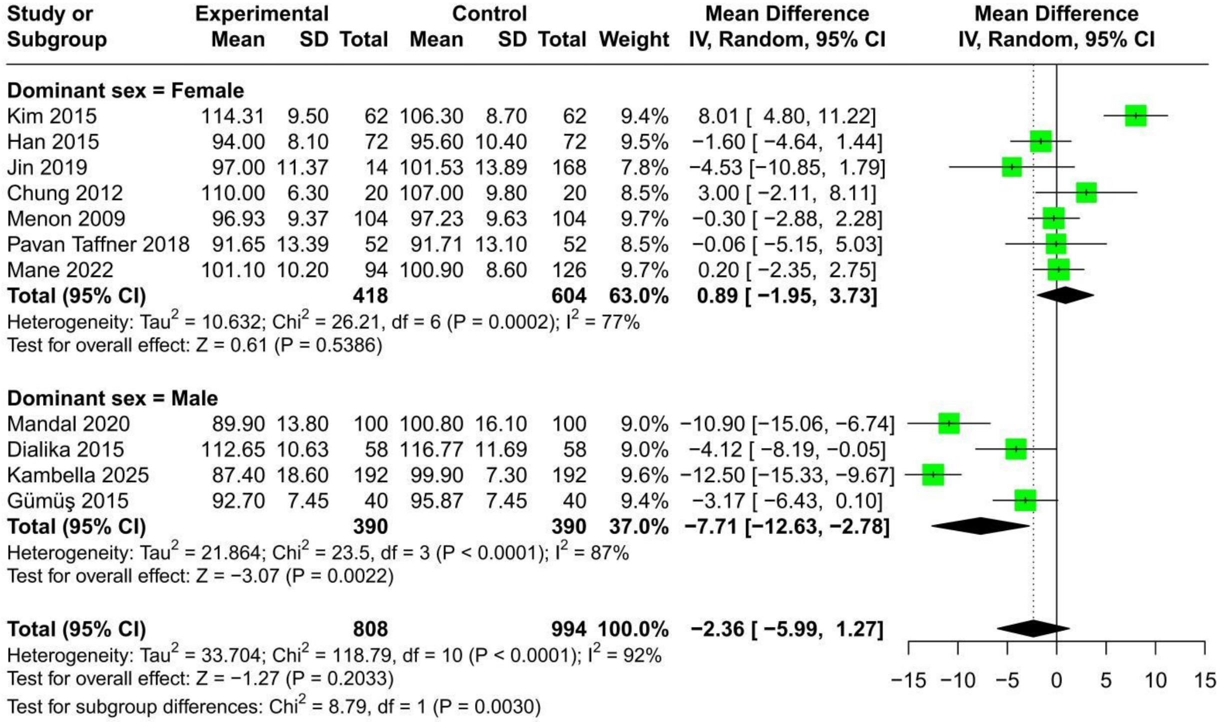

Figure S2. Continue.

A **iii. Mean age**

Study or Subgroup	Experimental Mean	Experimental SD	Experimental Total	Control Mean	Control SD	Control Total	Weight	Mean Difference IV, Random, 95% CI
**Mean age = Less than 40**								
Chung 2012	110.00	6.30	20	107.00	9.80	20	7.7%	3.00 [−2.11, 8.11]
Mandal 2020	89.90	13.80	100	100.80	16.10	100	8.2%	−10.90 [−15.06, −6.74]
Menon 2009	96.93	9.37	104	97.23	9.63	104	8.9%	−0.30 [−2.88, 2.28]
Dialika 2015	112.65	10.63	58	116.77	11.69	58	8.3%	−4.12 [−8.19, −0.05]
Mane 2022	101.10	10.20	94	100.90	8.60	126	8.9%	0.20 [−2.35, 2.75]
Gümüş 2015	92.70	7.45	40	95.87	7.45	40	8.6%	−3.17 [−6.43, 0.10]
**Total (95% CI)**			**416**			**448**	**50.7%**	**−2.51 [−5.78, 0.75]**
Heterogeneity: $Tau^2 = 13.230$; $Chi^2 = 27.35$, df = 5 (P < 0.0001); $I^2 = 82\%$								
Test for overall effect: Z = −1.51 (P = 0.1315)								
**Mean age = 40 or more**								
Kim 2015	114.31	9.50	62	106.30	8.70	62	8.7%	8.01 [4.80, 11.22]
Han 2015	94.00	8.10	72	95.60	10.40	72	8.7%	−1.60 [−4.64, 1.44]
Jin 2019	97.00	11.37	14	101.53	13.89	168	7.0%	−4.53 [−10.85, 1.79]
Pavan Taffner 2018	91.65	13.39	52	91.71	13.10	52	7.7%	−0.06 [−5.15, 5.03]
Kambella 2025	87.40	18.60	192	99.90	7.30	192	8.8%	−12.50 [−15.33, −9.67]
Findik 2019	92.96	7.40	28	96.16	7.25	28	8.4%	−3.21 [−7.04, 0.63]
**Total (95% CI)**			**420**			**574**	**49.3%**	**−2.31 [−8.78, 4.16]**
Heterogeneity: $Tau^2 = 60.844$; $Chi^2 = 90.95$, df = 5 (P < 0.0001); $I^2 = 95\%$								
Test for overall effect: Z = −0.70 (P = 0.4842)								
**Total (95% CI)**			**836**			**1022**	**100.0%**	**−2.43 [−5.77, 0.91]**
Heterogeneity: $Tau^2 = 30.867$; $Chi^2 = 119.01$, df = 11 (P < 0.0001); $I^2 = 91\%$								
Test for overall effect: Z = −1.42 (P = 0.1545)								
Test for subgroup differences: $Chi^2 = 0.00$, df = 1 (P = 0.9558)								

Mean Difference IV, Random, 95% CI

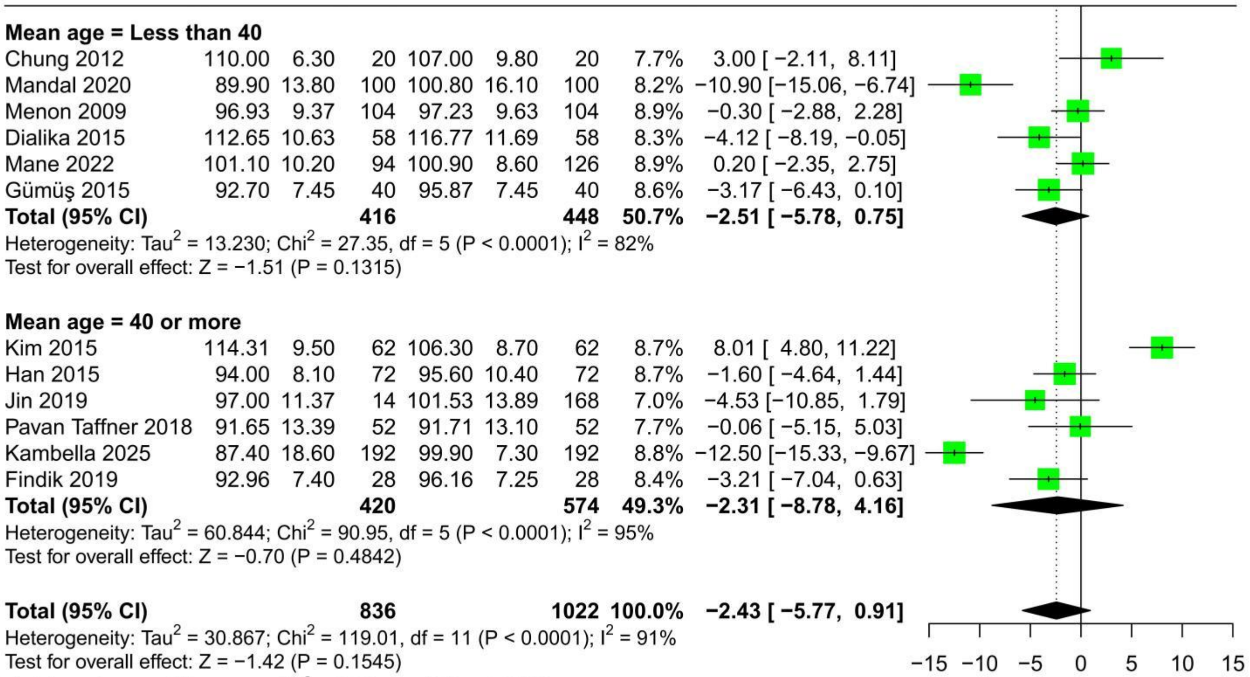

iiii. Device

Study or Subgroup	Experimental Mean	Experimental SD	Experimental Total	Control Mean	Control SD	Control Total	Weight	Mean Difference IV, Random, 95% CI
**Device = C**								
Han 2015	94.00	8.10	72	95.60	10.40	72	10.8%	−1.60 [−4.64, 1.44]
Jin 2019	97.00	11.37	14	101.53	13.89	168	8.1%	−4.53 [−10.85, 1.79]
Mandal 2020	89.90	13.80	100	100.80	16.10	100	10.0%	−10.90 [−15.06, −6.74]
Pavan Taffner 2018	91.65	13.39	52	91.71	13.10	52	9.2%	−0.06 [−5.15, 5.03]
Findik 2019	92.96	7.40	28	96.16	7.25	28	10.2%	−3.21 [−7.04, 0.63]
Gümüş 2015	92.70	7.45	40	95.87	7.45	40	10.7%	−3.17 [−6.43, 0.10]
**Total (95% CI)**			**306**			**460**	**58.9%**	**−3.88 [−6.81, −0.95]**
Heterogeneity: $Tau^2 = 8.771$; $Chi^2 = 15.55$, df = 5 (P = 0.0083); $I^2 = 68\%$								
Test for overall effect: Z = −2.59 (P = 0.0095)								
**Device = S**								
Kim 2015	114.31	9.50	62	106.30	8.70	62	10.7%	8.01 [4.80, 11.22]
Chung 2012	110.00	6.30	20	107.00	9.80	20	9.1%	3.00 [−2.11, 8.11]
Dialika 2015	112.65	10.63	58	116.77	11.69	58	10.0%	−4.12 [−8.19, −0.05]
**Total (95% CI)**			**140**			**140**	**29.9%**	**2.36 [−5.27, 9.98]**
Heterogeneity: $Tau^2 = 40.839$; $Chi^2 = 21.09$, df = 2 (P < 0.0001); $I^2 = 91\%$								
Test for overall effect: Z = 0.61 (P = 0.5446)								
**Device = Other devices**								
Menon 2009	96.93	9.37	104	97.23	9.63	104	11.2%	−0.30 [−2.88, 2.28]
**Total (95% CI)**			**550**			**704**	**100.0%**	**−1.61 [−4.74, 1.52]**
Heterogeneity: $Tau^2 = 21.141$; $Chi^2 = 61.42$, df = 9 (P < 0.0001); $I^2 = 85\%$								
Test for overall effect: Z = −1.01 (P = 0.3133)								
Test for subgroup differences: $Chi^2 = 4.32$, df = 2 (P = 0.1151)								

Mean Difference IV, Random, 95% CI

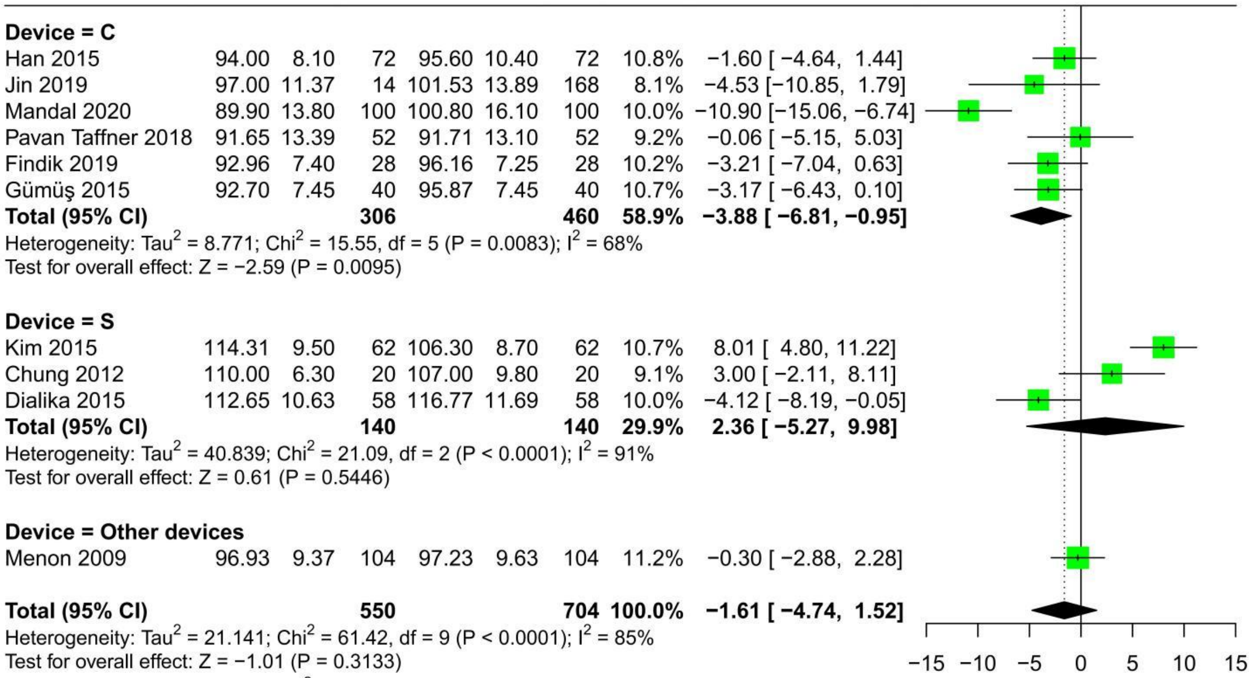

Figure S2. Continue.

B **i. Country**

Study or Subgroup	Experimental Mean	Experimental SD	Experimental Total	Control Mean	Control SD	Control Total	Weight	Mean Difference IV, Random, 95% CI
**Country = Korea**								
Kim 2015	135.00	13.60	62	136.10	12.30	62	7.7%	−1.10 [−5.66, 3.46]
Han 2015	118.50	15.70	72	119.80	20.40	72	7.1%	−1.30 [−7.25, 4.65]
Jin 2019	120.17	10.87	14	126.56	16.85	168	7.0%	−6.39 [−12.63, −0.15]
Chung 2012	134.80	14.40	20	131.60	14.20	20	5.8%	3.20 [−5.66, 12.06]
**Total (95% CI)**			**168**			**322**	**27.5%**	**−1.85 [−5.05, 1.35]**
Heterogeneity: Tau^2 = 1.353; Chi^2 = 3.42, df = 3 (P = 0.3315); I^2 = 12%								
Test for overall effect: Z = −1.13 (P = 0.2564)								
**Country = India**								
Mandal 2020	113.50	22.90	100	129.80	24.90	100	6.8%	−16.30 [−22.93, −9.67]
Menon 2009	123.87	16.14	104	126.36	17.03	104	7.7%	−2.49 [−7.00, 2.02]
Sarkar 2025	112.20	16.67	44	120.25	15.03	44	6.8%	−8.05 [−14.68, −1.42]
Mane 2022	144.00	15.10	94	134.00	17.60	126	7.8%	10.00 [5.67, 14.33]
Kambella 2025	111.30	27.20	192	122.70	11.10	192	7.9%	−11.40 [−15.56, −7.24]
**Total (95% CI)**			**534**			**566**	**37.0%**	**−5.50 [−14.75, 3.74]**
Heterogeneity: Tau^2 = 103.774; Chi^2 = 67.39, df = 4 (P < 0.0001); I^2 = 94%								
Test for overall effect: Z = −1.17 (P = 0.2433)								
**Country = Turkey**								
Findik 2019	117.37	11.95	28	122.94	12.45	28	6.9%	−5.57 [−11.97, 0.82]
Gümüş 2015	116.11	11.47	40	122.66	15.48	40	7.1%	−6.55 [−12.52, −0.58]
**Total (95% CI)**			**68**			**68**	**14.0%**	**−6.10 [−10.46, −1.73]**
Heterogeneity: Tau^2 = 0; Chi^2 = 0.05, df = 1 (P = 0.8263); I^2 = 0%								
Test for overall effect: Z = −2.74 (P = 0.0062)								
**Country = Other countries**								
Pavan Taffner 2018	112.12	21.46	52	112.90	22.51	52	5.9%	−0.78 [−9.23, 7.67]
Tevaraj 2017	134.42	8.16	72	132.85	9.17	72	8.3%	1.57 [−1.27, 4.41]
Dialika 2015	141.00	16.00	58	147.00	16.00	58	7.2%	−6.00 [−11.82, −0.18]
**Total (95% CI)**			**182**			**182**	**21.4%**	**−1.34 [−6.42, 3.74]**
Heterogeneity: Tau^2 = 12.338; Chi^2 = 5.29, df = 2 (P = 0.0711); I^2 = 62%								
Test for overall effect: Z = −0.52 (P = 0.6044)								
**Total (95% CI)**			**952**			**1138**	**100.0%**	**−3.60 [−7.21, 0.02]**
Heterogeneity: Tau^2 = 38.641; Chi^2 = 84.66, df = 13 (P < 0.0001); I^2 = 85%								
Test for overall effect: Z = −1.95 (P = 0.0511)								
Test for subgroup differences: Chi^2 = 3.11, df = 3 (P = 0.3744)								

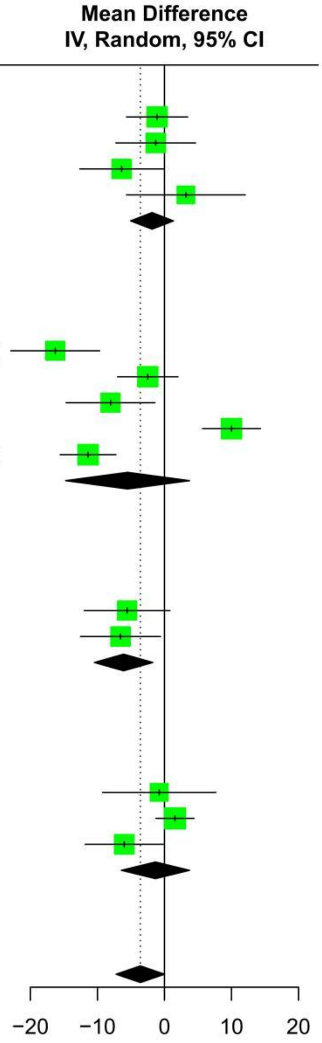

ii. Dominant sex

Study or Subgroup	Experimental Mean	Experimental SD	Experimental Total	Control Mean	Control SD	Control Total	Weight	Mean Difference IV, Random, 95% CI
**Dominant sex = Female**								
Kim 2015	135.00	13.60	62	136.10	12.30	62	8.3%	−1.10 [−5.66, 3.46]
Han 2015	118.50	15.70	72	119.80	20.40	72	7.6%	−1.30 [−7.25, 4.65]
Jin 2019	120.17	10.87	14	126.56	16.85	168	7.5%	−6.39 [−12.63, −0.15]
Chung 2012	134.80	14.40	20	131.60	14.20	20	6.2%	3.20 [−5.66, 12.06]
Menon 2009	123.87	16.14	104	126.36	17.03	104	8.3%	−2.49 [−7.00, 2.02]
Pavan Taffner 2018	112.12	21.46	52	112.90	22.51	52	6.4%	−0.78 [−9.23, 7.67]
Tevaraj 2017	134.42	8.16	72	132.85	9.17	72	8.9%	1.57 [−1.27, 4.41]
Sarkar 2025	112.20	16.67	44	120.25	15.03	44	7.3%	−8.05 [−14.68, −1.42]
Mane 2022	144.00	15.10	94	134.00	17.60	126	8.4%	10.00 [5.67, 14.33]
**Total (95% CI)**			**534**			**720**	**68.9%**	**−0.40 [−3.95, 3.16]**
Heterogeneity: Tau^2 = 20.954; Chi^2 = 33.05, df = 8 (P < 0.0001); I^2 = 76%								
Test for overall effect: Z = −0.22 (P = 0.8263)								
**Dominant sex = Male**								
Mandal 2020	113.50	22.90	100	129.80	24.90	100	7.3%	−16.30 [−22.93, −9.67]
Dialika 2015	141.00	16.00	58	147.00	16.00	58	7.7%	−6.00 [−11.82, −0.18]
Kambella 2025	111.30	27.20	192	122.70	11.10	192	8.4%	−11.40 [−15.56, −7.24]
Gümüş 2015	116.11	11.47	40	122.66	15.48	40	7.6%	−6.55 [−12.52, −0.58]
**Total (95% CI)**			**390**			**390**	**31.1%**	**−10.00 [−14.24, −5.75]**
Heterogeneity: Tau^2 = 10.592; Chi^2 = 7, df = 3 (P = 0.0720); I^2 = 57%								
Test for overall effect: Z = −4.62 (P < 0.0001)								
**Total (95% CI)**			**924**			**1110**	**100.0%**	**−3.45 [−7.29, 0.38]**
Heterogeneity: Tau^2 = 40.872; Chi^2 = 83.71, df = 12 (P < 0.0001); I^2 = 86%								
Test for overall effect: Z = −1.77 (P = 0.0774)								
Test for subgroup differences: Chi^2 = 11.55, df = 1 (P = 0.0007)								

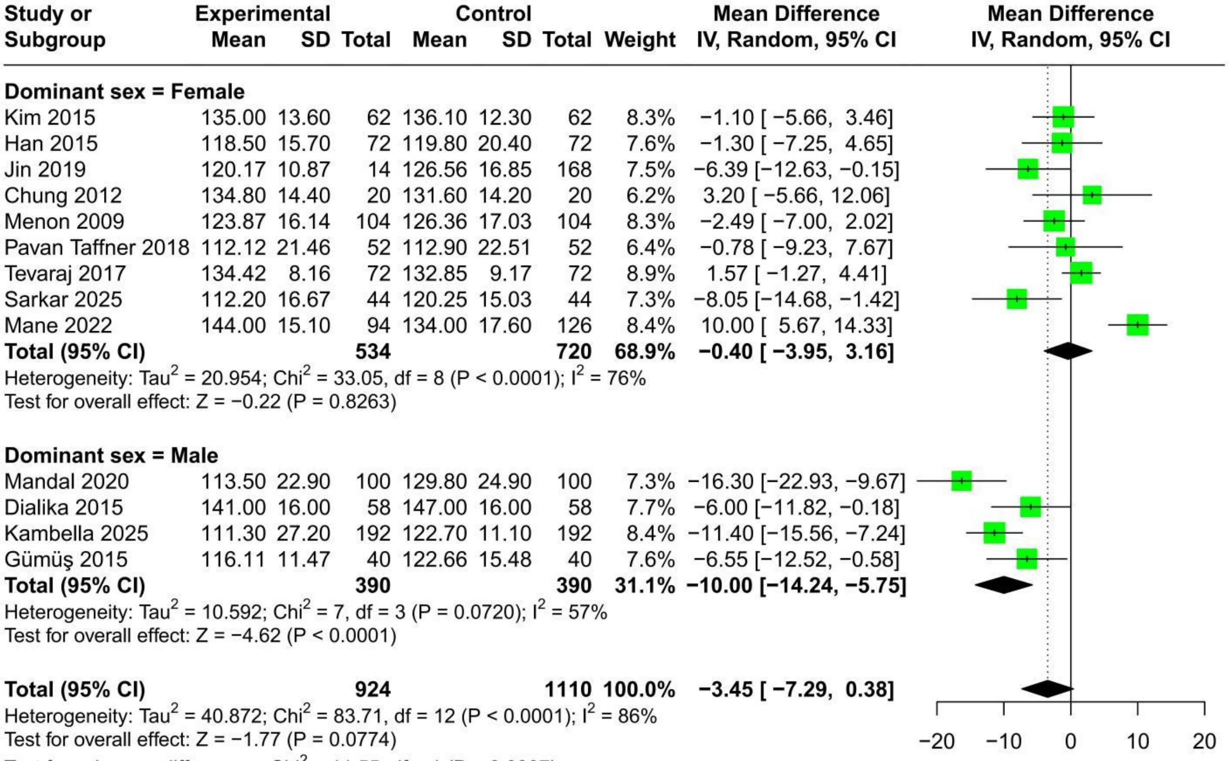

Figure S2. Continue.

B **iii. Mean age**

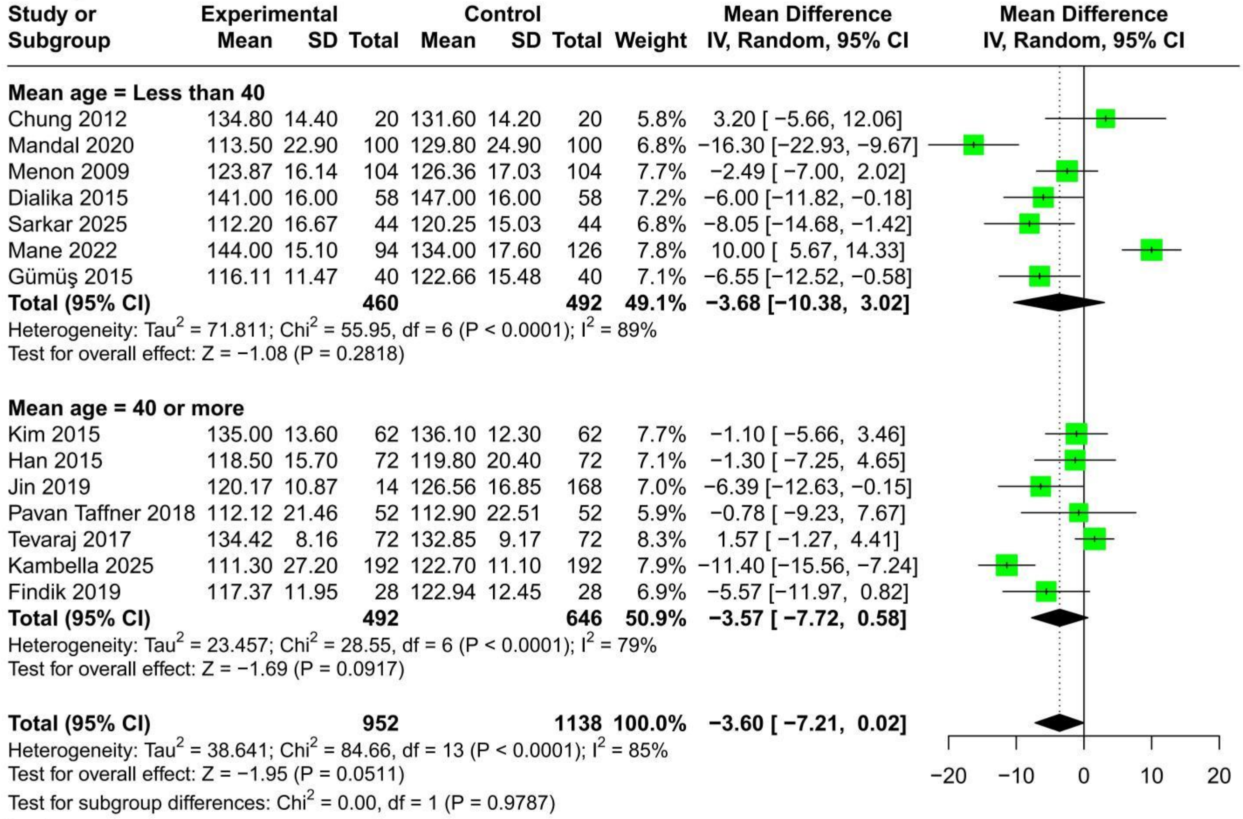

iiii. Device

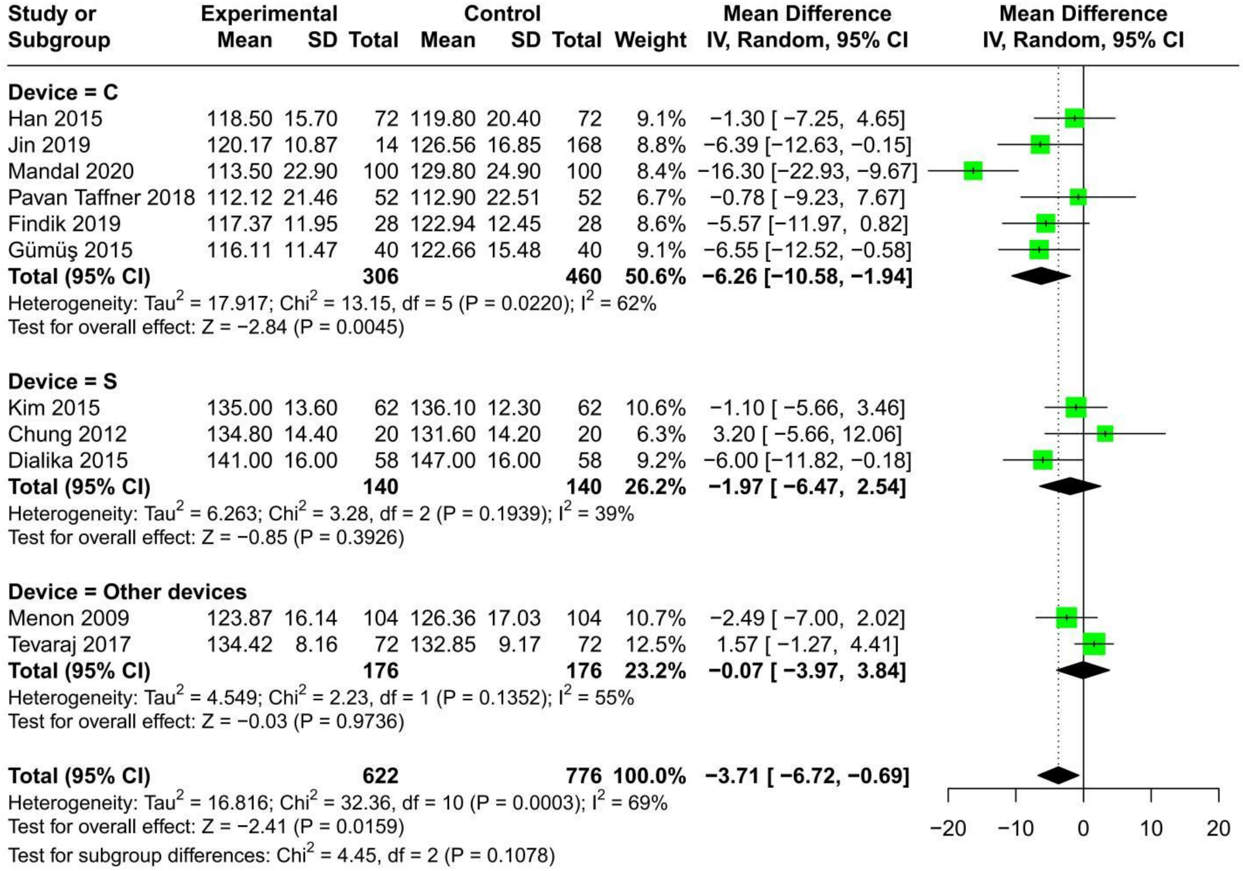

Figure S2. Continue.

C **i. Country**

Study or Subgroup	Experimental Mean	Experimental SD	Experimental Total	Control Mean	Control SD	Control Total	Weight	Mean Difference IV, Random, 95% CI
**Country = Korea**								
Kim 2015	142.10	11.80	62	137.21	13.10	62	8.3%	4.89 [0.50, 9.28]
Han 2015	123.00	16.50	72	123.20	17.60	72	7.7%	−0.20 [−5.77, 5.37]
Jin 2019	127.83	15.71	14	129.55	21.59	168	5.9%	−1.72 [−10.57, 7.13]
Chung 2012	142.20	12.10	20	136.60	15.60	20	6.0%	5.60 [−3.05, 14.25]
**Total (95% CI)**			**168**			**322**	**28.0%**	**2.62 [−0.66, 5.91]**
Heterogeneity: Tau^2 = 1.370; Chi^2 = 3.39, df = 3 (P = 0.3359); I^2 = 11%								
Test for overall effect: Z = 1.57 (P = 0.1175)								
**Country = India**								
Mandal 2020	115.90	21.50	100	126.10	24.60	100	7.2%	−10.20 [−16.60, −3.80]
Menon 2009	124.86	18.04	104	125.34	17.63	104	8.1%	−0.48 [−5.33, 4.37]
Sarkar 2025	117.50	17.48	44	123.40	32.27	44	5.0%	−5.90 [−16.74, 4.94]
Mane 2022	134.80	19.00	94	138.30	16.90	126	8.1%	−3.50 [−8.34, 1.34]
Kambella 2025	105.30	25.30	192	119.60	11.20	192	8.5%	−14.30 [−18.21, −10.39]
**Total (95% CI)**			**534**			**566**	**36.9%**	**−6.97 [−12.92, −1.02]**
Heterogeneity: Tau^2 = 36.230; Chi^2 = 23.02, df = 4 (P = 0.0001); I^2 = 83%								
Test for overall effect: Z = −2.29 (P = 0.0218)								
**Country = Turkey**								
Findik 2019	113.99	15.15	28	119.23	11.88	28	6.8%	−5.25 [−12.38, 1.89]
Gümüş 2015	118.92	12.89	40	123.27	15.48	40	7.3%	−4.36 [−10.60, 1.88]
**Total (95% CI)**			**68**			**68**	**14.2%**	**−4.74 [−9.44, −0.05]**
Heterogeneity: Tau^2 = 0; Chi^2 = 0.03, df = 1 (P = 0.8542); I^2 = 0%								
Test for overall effect: Z = −1.98 (P = 0.0478)								
**Country = Other countries**								
Pavan Taffner 2018	118.48	22.49	52	121.56	21.61	52	6.1%	−3.08 [−11.56, 5.40]
Tevaraj 2017	128.83	14.88	72	126.58	14.87	72	8.1%	2.25 [−2.61, 7.11]
Dialika 2015	144.00	19.00	58	146.00	21.00	58	6.8%	−2.00 [−9.29, 5.29]
**Total (95% CI)**			**182**			**182**	**20.9%**	**0.20 [−3.45, 3.85]**
Heterogeneity: Tau^2 = 0; Chi^2 = 1.61, df = 2 (P = 0.4473); I^2 = 0%								
Test for overall effect: Z = 0.11 (P = 0.9158)								
**Total (95% CI)**			**952**			**1138**	**100.0%**	**−2.77 [−6.25, 0.72]**
Heterogeneity: Tau^2 = 33.001; Chi^2 = 60.58, df = 13 (P < 0.0001); I^2 = 79%								
Test for overall effect: Z = −1.56 (P = 0.1195)								
Test for subgroup differences: Chi^2 = 11.27, df = 3 (P = 0.0104)								

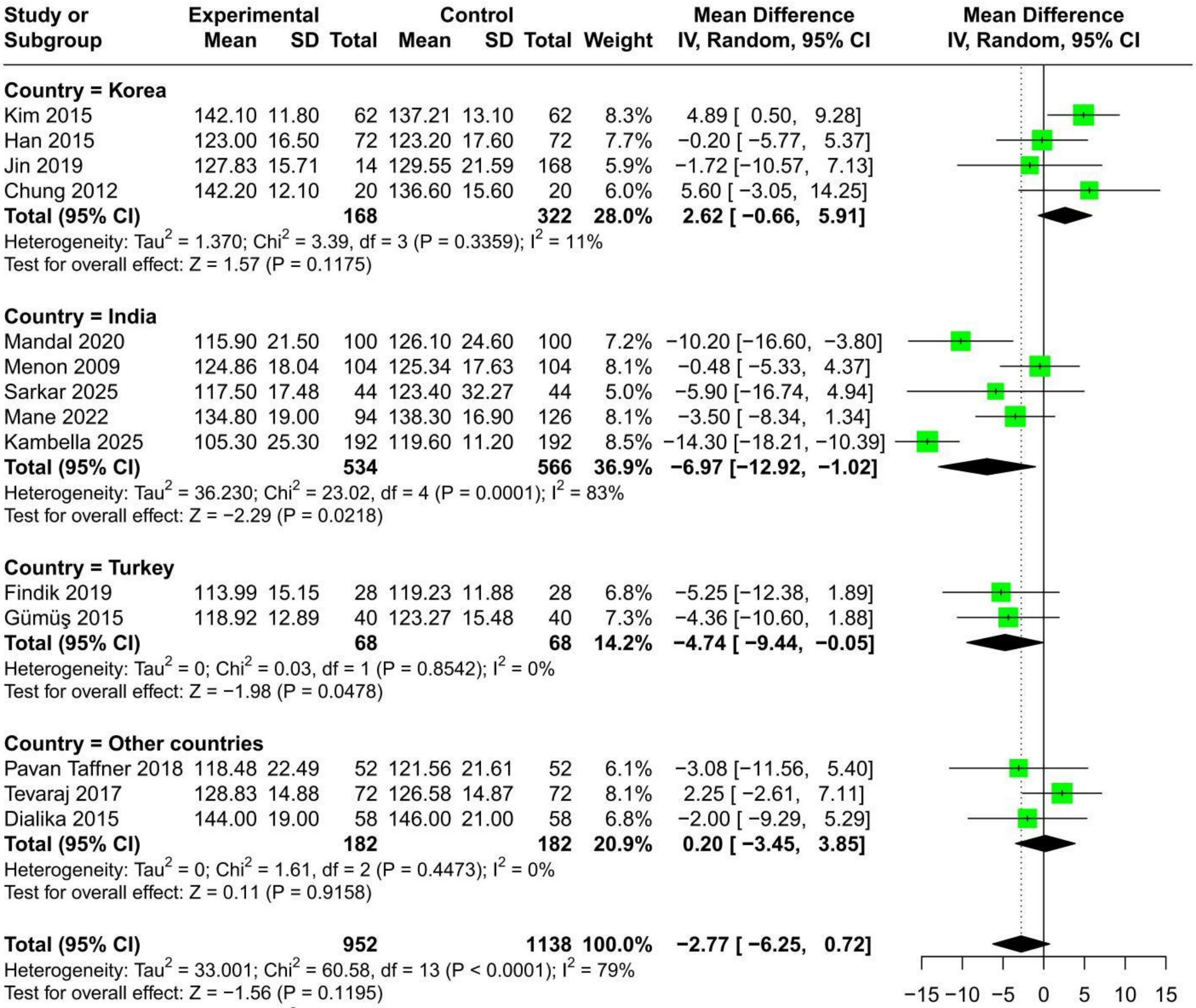

ii. Dominant sex

Study or Subgroup	Experimental Mean	Experimental SD	Experimental Total	Control Mean	Control SD	Control Total	Weight	Mean Difference IV, Random, 95% CI
**Dominant sex = Female**								
Kim 2015	142.10	11.80	62	137.21	13.10	62	8.9%	4.89 [0.50, 9.28]
Han 2015	123.00	16.50	72	123.20	17.60	72	8.2%	−0.20 [−5.77, 5.37]
Jin 2019	127.83	15.71	14	129.55	21.59	168	6.4%	−1.72 [−10.57, 7.13]
Chung 2012	142.20	12.10	20	136.60	15.60	20	6.5%	5.60 [−3.05, 14.25]
Menon 2009	124.86	18.04	104	125.34	17.63	104	8.6%	−0.48 [−5.33, 4.37]
Pavan Taffner 2018	118.48	22.49	52	121.56	21.61	52	6.6%	−3.08 [−11.56, 5.40]
Tevaraj 2017	128.83	14.88	72	126.58	14.87	72	8.6%	2.25 [−2.61, 7.11]
Sarkar 2025	117.50	17.48	44	123.40	32.27	44	5.4%	−5.90 [−16.74, 4.94]
Mane 2022	134.80	19.00	94	138.30	16.90	126	8.6%	−3.50 [−8.34, 1.34]
**Total (95% CI)**			**534**			**720**	**68.0%**	**0.36 [−2.00, 2.72]**
Heterogeneity: Tau^2 = 3.249; Chi^2 = 10.78, df = 8 (P = 0.2145); I^2 = 26%								
Test for overall effect: Z = 0.30 (P = 0.7660)								
**Dominant sex = Male**								
Mandal 2020	115.90	21.50	100	126.10	24.60	100	7.8%	−10.20 [−16.60, −3.80]
Dialika 2015	144.00	19.00	58	146.00	21.00	58	7.3%	−2.00 [−9.29, 5.29]
Kambella 2025	105.30	25.30	192	119.60	11.20	192	9.1%	−14.30 [−18.21, −10.39]
Gümüş 2015	118.92	12.89	40	123.27	15.48	40	7.9%	−4.36 [−10.60, 1.88]
**Total (95% CI)**			**390**			**390**	**32.0%**	**−8.15 [−14.06, −2.24]**
Heterogeneity: Tau^2 = 27.084; Chi^2 = 12.41, df = 3 (P = 0.0061); I^2 = 76%								
Test for overall effect: Z = −2.70 (P = 0.0069)								
**Total (95% CI)**			**924**			**1110**	**100.0%**	**−2.58 [−6.29, 1.13]**
Heterogeneity: Tau^2 = 35.396; Chi^2 = 60.23, df = 12 (P < 0.0001); I^2 = 80%								
Test for overall effect: Z = −1.37 (P = 0.1722)								
Test for subgroup differences: Chi^2 = 6.87, df = 1 (P = 0.0088)								

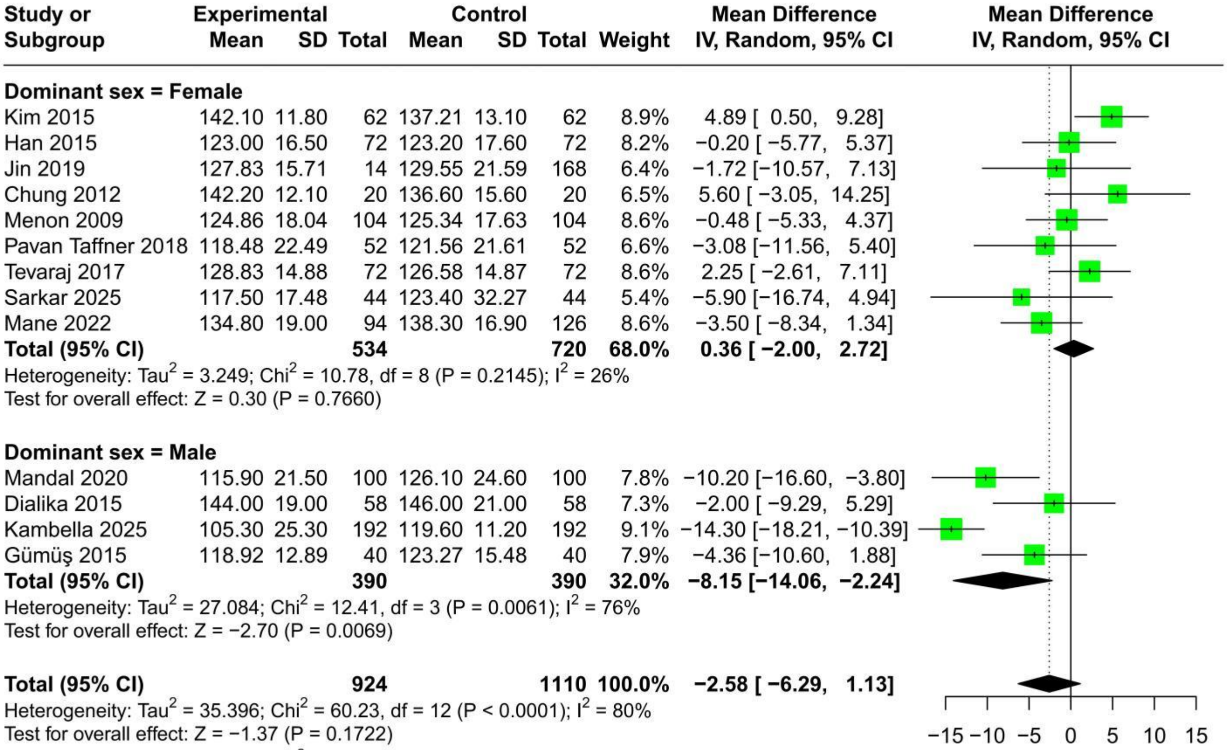

Figure S2. Continue.

C **iii. Mean age**

Study or Subgroup	Experimental Mean	SD	Total	Control Mean	SD	Total	Weight	Mean Difference IV, Random, 95% CI
**Mean age = Less than 40**								
Chung 2012	142.20	12.10	20	136.60	15.60	20	6.0%	5.60 [−3.05, 14.25]
Mandal 2020	115.90	21.50	100	126.10	24.60	100	7.2%	−10.20 [−16.60, −3.80]
Menon 2009	124.86	18.04	104	125.34	17.63	104	8.1%	−0.48 [−5.33, 4.37]
Dialika 2015	144.00	19.00	58	146.00	21.00	58	6.8%	−2.00 [−9.29, 5.29]
Sarkar 2025	117.50	17.48	44	123.40	32.27	44	5.0%	−5.90 [−16.74, 4.94]
Mane 2022	134.80	19.00	94	138.30	16.90	126	8.1%	−3.50 [−8.34, 1.34]
Gümüş 2015	118.92	12.89	40	123.27	15.48	40	7.3%	−4.36 [−10.60, 1.88]
**Total (95% CI)**			**460**			**492**	**48.5%**	**−3.10 [−6.34, 0.15]**
Heterogeneity: $Tau^2 = 7.651$; $Chi^2 = 10.25$, df = 6 (P = 0.1144); $I^2 = 41\%$								
Test for overall effect: Z = −1.87 (P = 0.0613)								
**Mean age = 40 or more**								
Kim 2015	142.10	11.80	62	137.21	13.10	62	8.3%	4.89 [0.50, 9.28]
Han 2015	123.00	16.50	72	123.20	17.60	72	7.7%	−0.20 [−5.77, 5.37]
Jin 2019	127.83	15.71	14	129.55	21.59	168	5.9%	−1.72 [−10.57, 7.13]
Pavan Taffner 2018	118.48	22.49	52	121.56	21.61	52	6.1%	−3.08 [−11.56, 5.40]
Tevaraj 2017	128.83	14.88	72	126.58	14.87	72	8.1%	2.25 [−2.61, 7.11]
Kambella 2025	105.30	25.30	192	119.60	11.20	192	8.5%	−14.30 [−18.21, −10.39]
Findik 2019	113.99	15.15	28	119.23	11.88	28	6.8%	−5.25 [−12.38, 1.89]
**Total (95% CI)**			**492**			**646**	**51.5%**	**−2.50 [−8.69, 3.69]**
Heterogeneity: $Tau^2 = 59.518$; $Chi^2 = 50.33$, df = 6 (P < 0.0001); $I^2 = 88\%$								
Test for overall effect: Z = −0.79 (P = 0.4279)								
**Total (95% CI)**			**952**			**1138**	**100.0%**	**−2.77 [−6.25, 0.72]**
Heterogeneity: $Tau^2 = 33.001$; $Chi^2 = 60.58$, df = 13 (P < 0.0001); $I^2 = 79\%$								
Test for overall effect: Z = −1.56 (P = 0.1195)								
Test for subgroup differences: $Chi^2 = 0.03$, df = 1 (P = 0.8681)								

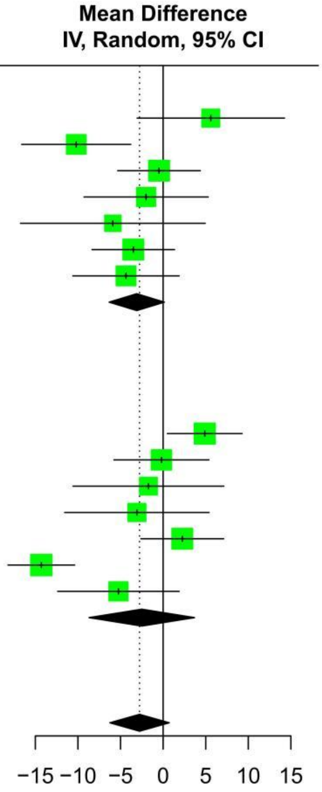

iiii. Device

Study or Subgroup	Experimental Mean	SD	Total	Control Mean	SD	Total	Weight	Mean Difference IV, Random, 95% CI
**Device = C**								
Han 2015	123.00	16.50	72	123.20	17.60	72	10.4%	−0.20 [−5.77, 5.37]
Jin 2019	127.83	15.71	14	129.55	21.59	168	6.3%	−1.72 [−10.57, 7.13]
Mandal 2020	115.90	21.50	100	126.10	24.60	100	9.1%	−10.20 [−16.60, −3.80]
Pavan Taffner 2018	118.48	22.49	52	121.56	21.61	52	6.7%	−3.08 [−11.56, 5.40]
Findik 2019	113.99	15.15	28	119.23	11.88	28	8.2%	−5.25 [−12.38, 1.89]
Gümüş 2015	118.92	12.89	40	123.27	15.48	40	9.4%	−4.36 [−10.60, 1.88]
**Total (95% CI)**			**306**			**460**	**50.1%**	**−4.18 [−7.21, −1.16]**
Heterogeneity: $Tau^2 = 1.995$; $Chi^2 = 5.8$, df = 5 (P = 0.3257); $I^2 = 14\%$								
Test for overall effect: Z = −2.71 (P = 0.0067)								
**Device = S**								
Kim 2015	142.10	11.80	62	137.21	13.10	62	12.3%	4.89 [0.50, 9.28]
Chung 2012	142.20	12.10	20	136.60	15.60	20	6.5%	5.60 [−3.05, 14.25]
Dialika 2015	144.00	19.00	58	146.00	21.00	58	8.0%	−2.00 [−9.29, 5.29]
**Total (95% CI)**			**140**			**140**	**26.8%**	**3.17 [−1.21, 7.54]**
Heterogeneity: $Tau^2 = 4.549$; $Chi^2 = 2.8$, df = 2 (P = 0.2467); $I^2 = 29\%$								
Test for overall effect: Z = 1.42 (P = 0.1559)								
**Device = Other devices**								
Menon 2009	124.86	18.04	104	125.34	17.63	104	11.5%	−0.48 [−5.33, 4.37]
Tevaraj 2017	128.83	14.88	72	126.58	14.87	72	11.5%	2.25 [−2.61, 7.11]
**Total (95% CI)**			**176**			**176**	**23.0%**	**0.88 [−2.55, 4.31]**
Heterogeneity: $Tau^2 = 0$; $Chi^2 = 0.61$, df = 1 (P = 0.4357); $I^2 = 0\%$								
Test for overall effect: Z = 0.50 (P = 0.6145)								
**Total (95% CI)**			**622**			**776**	**100.0%**	**−1.10 [−3.88, 1.69]**
Heterogeneity: $Tau^2 = 11.334$; $Chi^2 = 21.51$, df = 10 (P = 0.0178); $I^2 = 54\%$								
Test for overall effect: Z = −0.77 (P = 0.4400)								
Test for subgroup differences: $Chi^2 = 8.88$, df = 2 (P = 0.0118)								

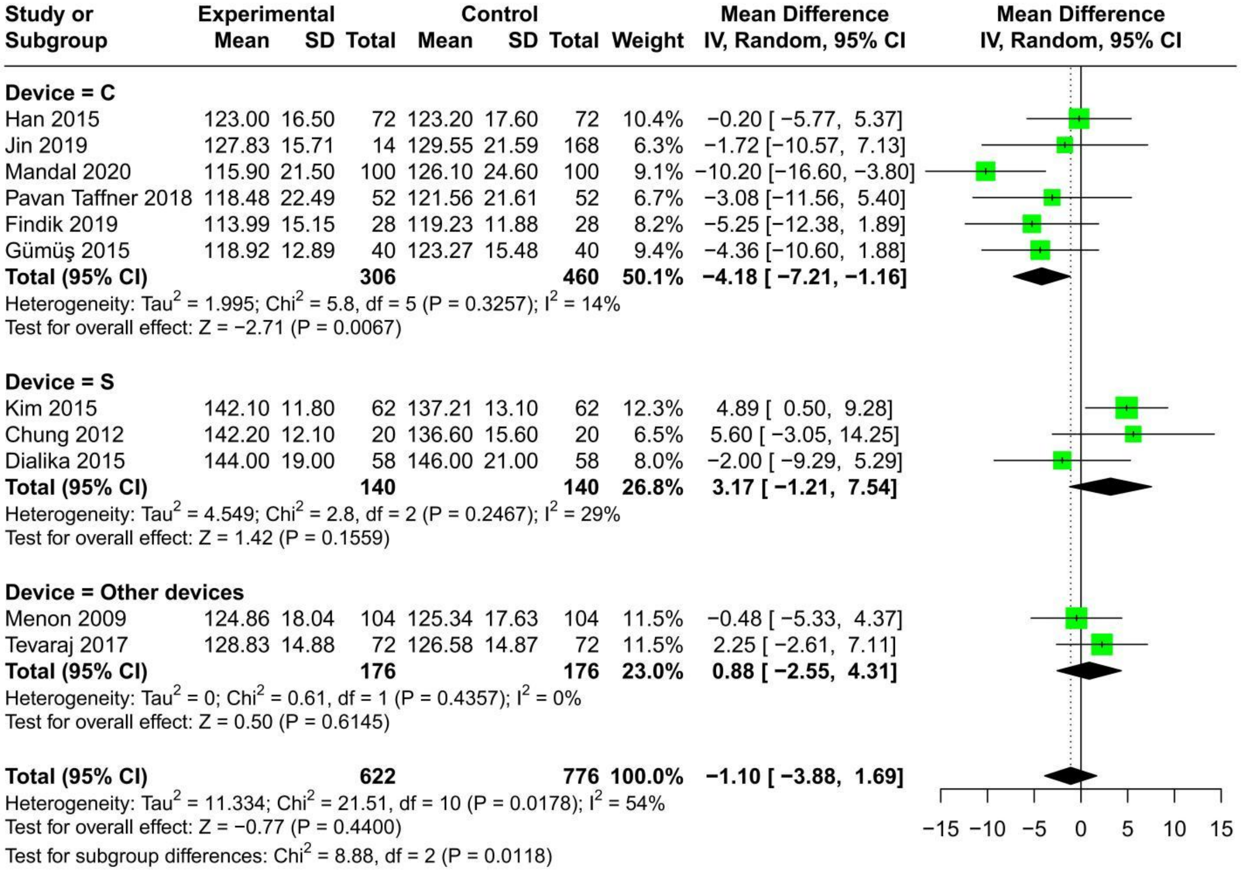

Figure S2. Continue.

D **i. Country**

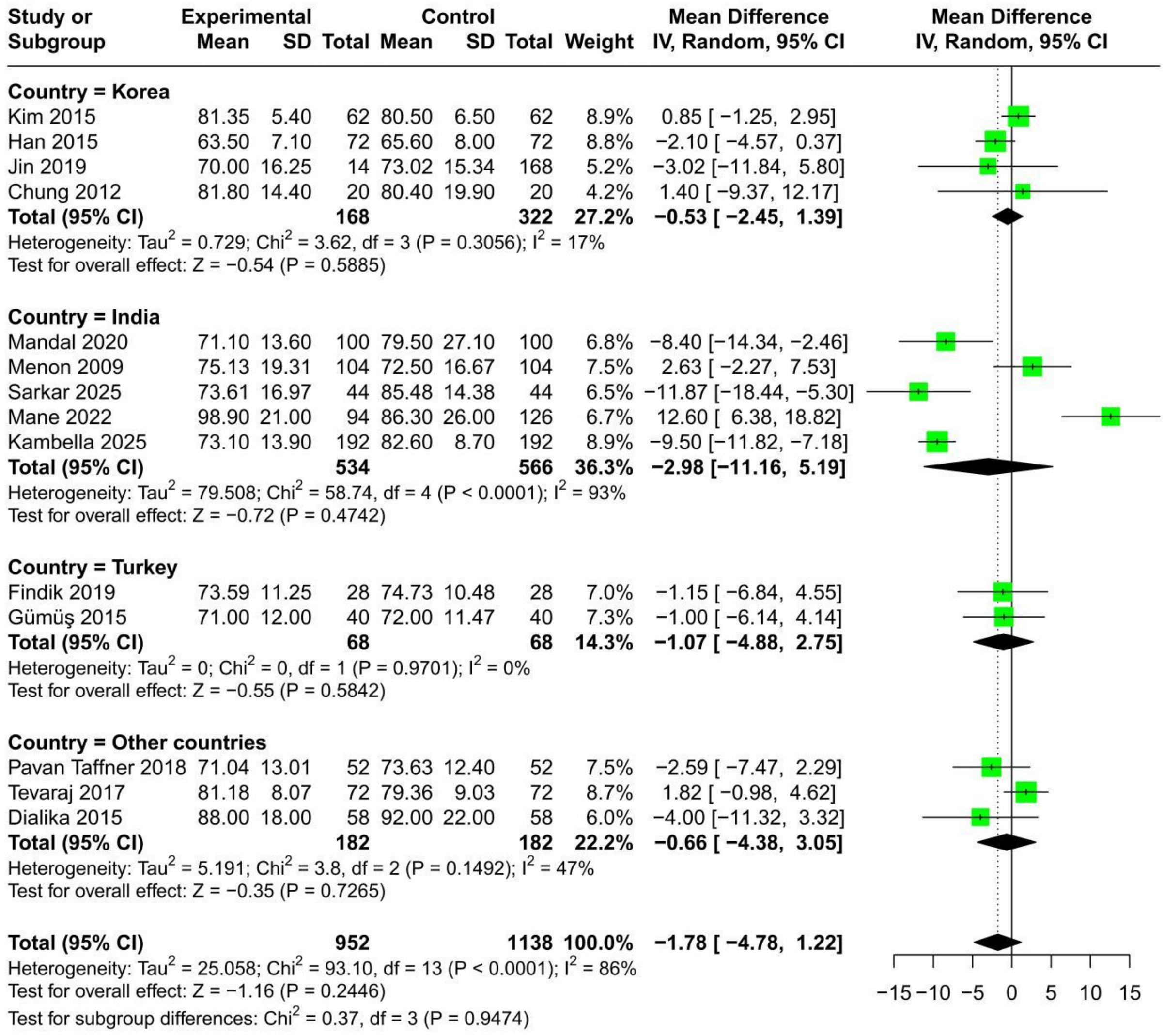

ii. Dominant sex

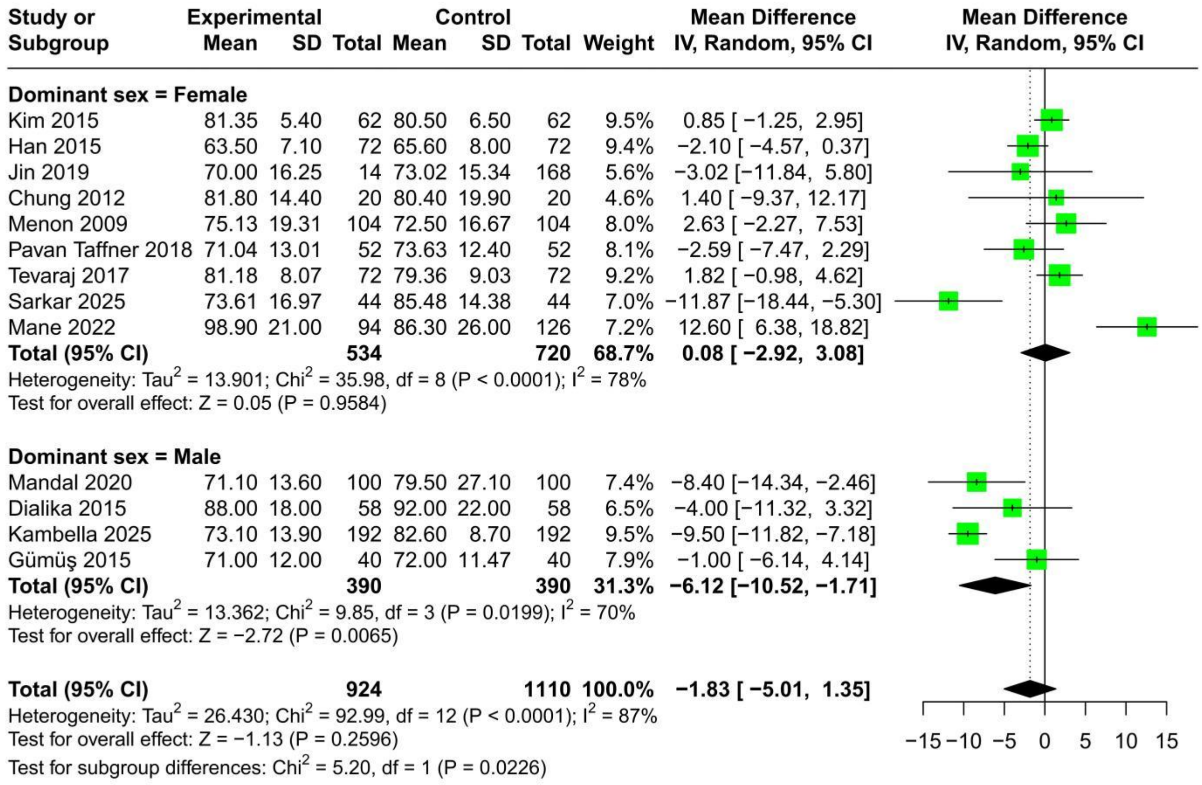

Figure S2. Continue.

D **iii. Mean age**

Study or Subgroup	Experimental Mean	SD	Total	Control Mean	SD	Total	Weight	Mean Difference IV, Random, 95% CI
**Mean age = Less than 40**								
Chung 2012	81.80	14.40	20	80.40	19.90	20	4.2%	1.40 [−9.37, 12.17]
Mandal 2020	71.10	13.60	100	79.50	27.10	100	6.8%	−8.40 [−14.34, −2.46]
Menon 2009	75.13	19.31	104	72.50	16.67	104	7.5%	2.63 [−2.27, 7.53]
Dialika 2015	88.00	18.00	58	92.00	22.00	58	6.0%	−4.00 [−11.32, 3.32]
Sarkar 2025	73.61	16.97	44	85.48	14.38	44	6.5%	−11.87 [−18.44, −5.30]
Mane 2022	98.90	21.00	94	86.30	26.00	126	6.7%	12.60 [6.38, 18.82]
Gümüş 2015	71.00	12.00	40	72.00	11.47	40	7.3%	−1.00 [−6.14, 4.14]
**Total (95% CI)**			**460**			**492**	**45.1%**	**−1.25 [−7.25, 4.75]**
Heterogeneity: $Tau^2 = 53.859$; $Chi^2 = 37.8$, df = 6 (P < 0.0001); $I^2 = 84\%$								
Test for overall effect: Z = −0.41 (P = 0.6827)								
**Mean age = 40 or more**								
Kim 2015	81.35	5.40	62	80.50	6.50	62	8.9%	0.85 [−1.25, 2.95]
Han 2015	63.50	7.10	72	65.60	8.00	72	8.8%	−2.10 [−4.57, 0.37]
Jin 2019	70.00	16.25	14	73.02	15.34	168	5.2%	−3.02 [−11.84, 5.80]
Pavan Taffner 2018	71.04	13.01	52	73.63	12.40	52	7.5%	−2.59 [−7.47, 2.29]
Tevaraj 2017	81.18	8.07	72	79.36	9.03	72	8.7%	1.82 [−0.98, 4.62]
Kambella 2025	73.10	13.90	192	82.60	8.70	192	8.9%	−9.50 [−11.82, −7.18]
Findik 2019	73.59	11.25	28	74.73	10.48	28	7.0%	−1.15 [−6.84, 4.55]
**Total (95% CI)**			**492**			**646**	**54.9%**	**−2.23 [−5.92, 1.46]**
Heterogeneity: $Tau^2 = 20.153$; $Chi^2 = 54.17$, df = 6 (P < 0.0001); $I^2 = 89\%$								
Test for overall effect: Z = −1.18 (P = 0.2366)								
**Total (95% CI)**			**952**			**1138**	**100.0%**	**−1.78 [−4.78, 1.22]**
Heterogeneity: $Tau^2 = 25.058$; $Chi^2 = 93.10$, df = 13 (P < 0.0001); $I^2 = 86\%$								
Test for overall effect: Z = −1.16 (P = 0.2446)								
Test for subgroup differences: $Chi^2 = 0.07$, df = 1 (P = 0.7859)								

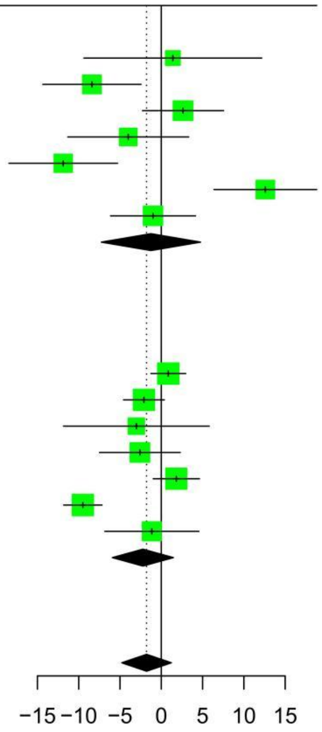

iiii. Device

Study or Subgroup	Experimental Mean	SD	Total	Control Mean	SD	Total	Weight	Mean Difference IV, Random, 95% CI
**Device = C**								
Han 2015	63.50	7.10	72	65.60	8.00	72	17.4%	−2.10 [−4.57, 0.37]
Jin 2019	70.00	16.25	14	73.02	15.34	168	3.3%	−3.02 [−11.84, 5.80]
Mandal 2020	71.10	13.60	100	79.50	27.10	100	6.3%	−8.40 [−14.34, −2.46]
Pavan Taffner 2018	71.04	13.01	52	73.63	12.40	52	8.4%	−2.59 [−7.47, 2.29]
Findik 2019	73.59	11.25	28	74.73	10.48	28	6.7%	−1.15 [−6.84, 4.55]
Gümüş 2015	71.00	12.00	40	72.00	11.47	40	7.8%	−1.00 [−6.14, 4.14]
**Total (95% CI)**			**306**			**460**	**49.8%**	**−2.54 [−4.32, −0.76]**
Heterogeneity: $Tau^2 = 0$; $Chi^2 = 4.44$, df = 5 (P = 0.4876); $I^2 = 0\%$								
Test for overall effect: Z = −2.80 (P = 0.0051)								
**Device = S**								
Kim 2015	81.35	5.40	62	80.50	6.50	62	19.3%	0.85 [−1.25, 2.95]
Chung 2012	81.80	14.40	20	80.40	19.90	20	2.3%	1.40 [−9.37, 12.17]
Dialika 2015	88.00	18.00	58	92.00	22.00	58	4.5%	−4.00 [−11.32, 3.32]
**Total (95% CI)**			**140**			**140**	**26.1%**	**0.51 [−1.48, 2.50]**
Heterogeneity: $Tau^2 = 0$; $Chi^2 = 1.59$, df = 2 (P = 0.4523); $I^2 = 0\%$								
Test for overall effect: Z = 0.50 (P = 0.6142)								
**Device = Other devices**								
Menon 2009	75.13	19.31	104	72.50	16.67	104	8.3%	2.63 [−2.27, 7.53]
Tevaraj 2017	81.18	8.07	72	79.36	9.03	72	15.7%	1.82 [−0.98, 4.62]
**Total (95% CI)**			**176**			**176**	**24.1%**	**2.02 [−0.41, 4.45]**
Heterogeneity: $Tau^2 = 0$; $Chi^2 = 0.08$, df = 1 (P = 0.7785); $I^2 = 0\%$								
Test for overall effect: Z = 1.63 (P = 0.1034)								
**Total (95% CI)**			**622**			**776**	**100.0%**	**−0.84 [−2.54, 0.86]**
Heterogeneity: $Tau^2 = 2.723$; $Chi^2 = 16.28$, df = 10 (P = 0.0918); $I^2 = 39\%$								
Test for overall effect: Z = −0.97 (P = 0.3311)								
Test for subgroup differences: $Chi^2 = 10.17$, df = 2 (P = 0.0062)								

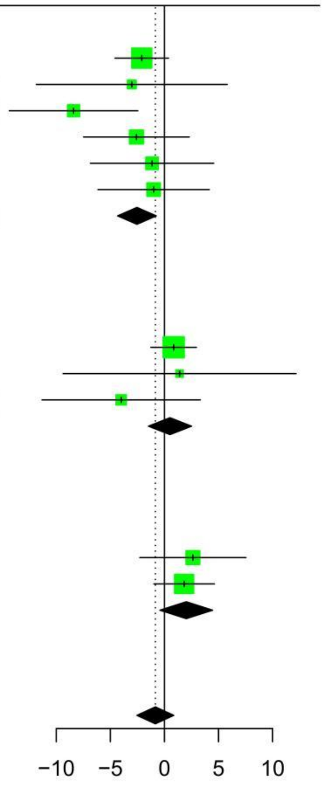

Figure S2. Continue.

E **i. Country**

Study or Subgroup	Experimental Mean	SD	Total	Control Mean	SD	Total	Weight	Mean Difference IV, Random, 95% CI
**Country = Korea**								
Kim 2015	83.85	8.50	62	80.53	10.40	62	8.0%	3.32 [−0.02, 6.66]
Han 2015	71.30	10.60	72	73.30	12.90	72	7.6%	−2.00 [−5.86, 1.86]
Jin 2019	71.17	16.95	14	78.81	16.55	168	4.2%	−7.64 [−16.86, 1.58]
Chung 2012	82.20	12.70	20	79.80	12.60	20	4.9%	2.40 [−5.44, 10.24]
**Total (95% CI)**			**168**			**322**	**24.7%**	**−0.16 [−4.39, 4.07]**
Heterogeneity: $Tau^2 = 10.337$; $Chi^2 = 7.56$, df = 3 (P = 0.0561); $I^2 = 60\%$								
Test for overall effect: Z = −0.07 (P = 0.9412)								
**Country = India**								
Mandal 2020	59.60	15.10	100	67.90	22.50	100	6.6%	−8.30 [−13.61, −2.99]
Menon 2009	60.17	9.25	104	63.49	11.14	104	8.3%	−3.32 [−6.10, −0.54]
Sarkar 2025	68.84	11.78	44	72.52	11.02	44	7.0%	−3.68 [−8.45, 1.09]
Mane 2022	71.80	12.00	94	71.80	11.80	126	8.1%	0.00 [−3.18, 3.18]
Kambella 2025	58.20	13.60	192	66.70	8.10	192	8.6%	−8.50 [−10.74, −6.26]
**Total (95% CI)**			**534**			**566**	**38.6%**	**−4.69 [−8.22, −1.16]**
Heterogeneity: $Tau^2 = 12.690$; $Chi^2 = 22.03$, df = 4 (P = 0.0002); $I^2 = 82\%$								
Test for overall effect: Z = −2.60 (P = 0.0093)								
**Country = Turkey**								
Findik 2019	63.44	8.44	28	65.52	7.46	28	7.4%	−2.08 [−6.25, 2.09]
Gümüş 2015	63.43	7.45	40	63.57	7.45	40	8.0%	−0.13 [−3.40, 3.13]
**Total (95% CI)**			**68**			**68**	**15.4%**	**−0.87 [−3.44, 1.70]**
Heterogeneity: $Tau^2 = 0$; $Chi^2 = 0.52$, df = 1 (P = 0.4719); $I^2 = 0\%$								
Test for overall effect: Z = −0.66 (P = 0.5064)								
**Country = Other countries**								
Pavan Taffner 2018	61.63	15.89	52	61.13	11.69	52	6.5%	0.50 [−4.86, 5.86]
Tevaraj 2017	80.49	10.41	72	74.96	8.92	72	8.1%	5.53 [2.36, 8.70]
Dialika 2015	78.00	13.00	58	81.00	15.00	58	6.7%	−3.00 [−8.11, 2.11]
**Total (95% CI)**			**182**			**182**	**21.4%**	**1.32 [−4.03, 6.67]**
Heterogeneity: $Tau^2 = 16.941$; $Chi^2 = 8.49$, df = 2 (P = 0.0143); $I^2 = 76\%$								
Test for overall effect: Z = 0.48 (P = 0.6292)								
**Total (95% CI)**			**952**			**1138**	**100.0%**	**−1.79 [−4.33, 0.76]**
Heterogeneity: $Tau^2 = 18.282$; $Chi^2 = 76.92$, df = 13 (P < 0.0001); $I^2 = 83\%$								
Test for overall effect: Z = −1.38 (P = 0.1689)								
Test for subgroup differences: $Chi^2 = 4.81$, df = 3 (P = 0.1862)								

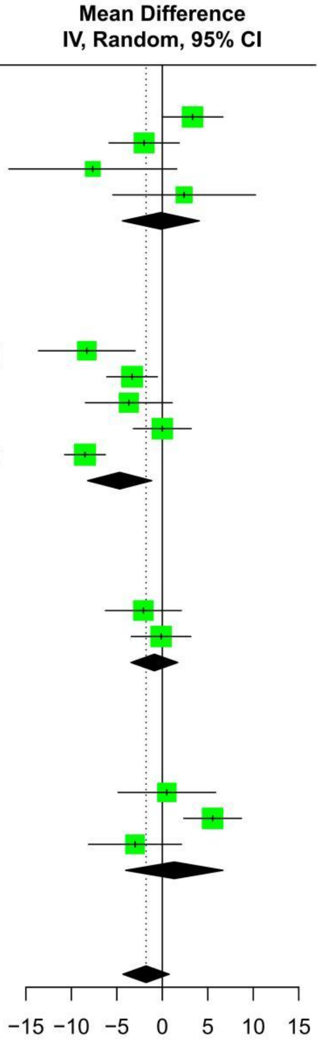

ii. Dominant sex

Study or Subgroup	Experimental Mean	SD	Total	Control Mean	SD	Total	Weight	Mean Difference IV, Random, 95% CI
**Dominant sex = Female**								
Kim 2015	83.85	8.50	62	80.53	10.40	62	8.6%	3.32 [−0.02, 6.66]
Han 2015	71.30	10.60	72	73.30	12.90	72	8.2%	−2.00 [−5.86, 1.86]
Jin 2019	71.17	16.95	14	78.81	16.55	168	4.6%	−7.64 [−16.86, 1.58]
Chung 2012	82.20	12.70	20	79.80	12.60	20	5.4%	2.40 [−5.44, 10.24]
Menon 2009	60.17	9.25	104	63.49	11.14	104	8.9%	−3.32 [−6.10, −0.54]
Pavan Taffner 2018	61.63	15.89	52	61.13	11.69	52	7.1%	0.50 [−4.86, 5.86]
Tevaraj 2017	80.49	10.41	72	74.96	8.92	72	8.7%	5.53 [2.36, 8.70]
Sarkar 2025	68.84	11.78	44	72.52	11.02	44	7.6%	−3.68 [−8.45, 1.09]
Mane 2022	71.80	12.00	94	71.80	11.80	126	8.7%	0.00 [−3.18, 3.18]
**Total (95% CI)**			**534**			**720**	**67.7%**	**−0.13 [−2.70, 2.45]**
Heterogeneity: $Tau^2 = 9.985$; $Chi^2 = 27.27$, df = 8 (P = 0.0006); $I^2 = 71\%$								
Test for overall effect: Z = −0.10 (P = 0.9241)								
**Dominant sex = Male**								
Mandal 2020	59.60	15.10	100	67.90	22.50	100	7.2%	−8.30 [−13.61, −2.99]
Dialika 2015	78.00	13.00	58	81.00	15.00	58	7.3%	−3.00 [−8.11, 2.11]
Kambella 2025	58.20	13.60	192	66.70	8.10	192	9.2%	−8.50 [−10.74, −6.26]
Gümüş 2015	63.43	7.45	40	63.57	7.45	40	8.6%	−0.13 [−3.40, 3.13]
**Total (95% CI)**			**390**			**390**	**32.3%**	**−4.98 [−9.66, −0.30]**
Heterogeneity: $Tau^2 = 18.515$; $Chi^2 = 19.16$, df = 3 (P = 0.0003); $I^2 = 84\%$								
Test for overall effect: Z = −2.09 (P = 0.0369)								
**Total (95% CI)**			**924**			**1110**	**100.0%**	**−1.77 [−4.51, 0.97]**
Heterogeneity: $Tau^2 = 19.886$; $Chi^2 = 76.92$, df = 12 (P < 0.0001); $I^2 = 84\%$								
Test for overall effect: Z = −1.27 (P = 0.2050)								
Test for subgroup differences: $Chi^2 = 3.18$, df = 1 (P = 0.0746)								

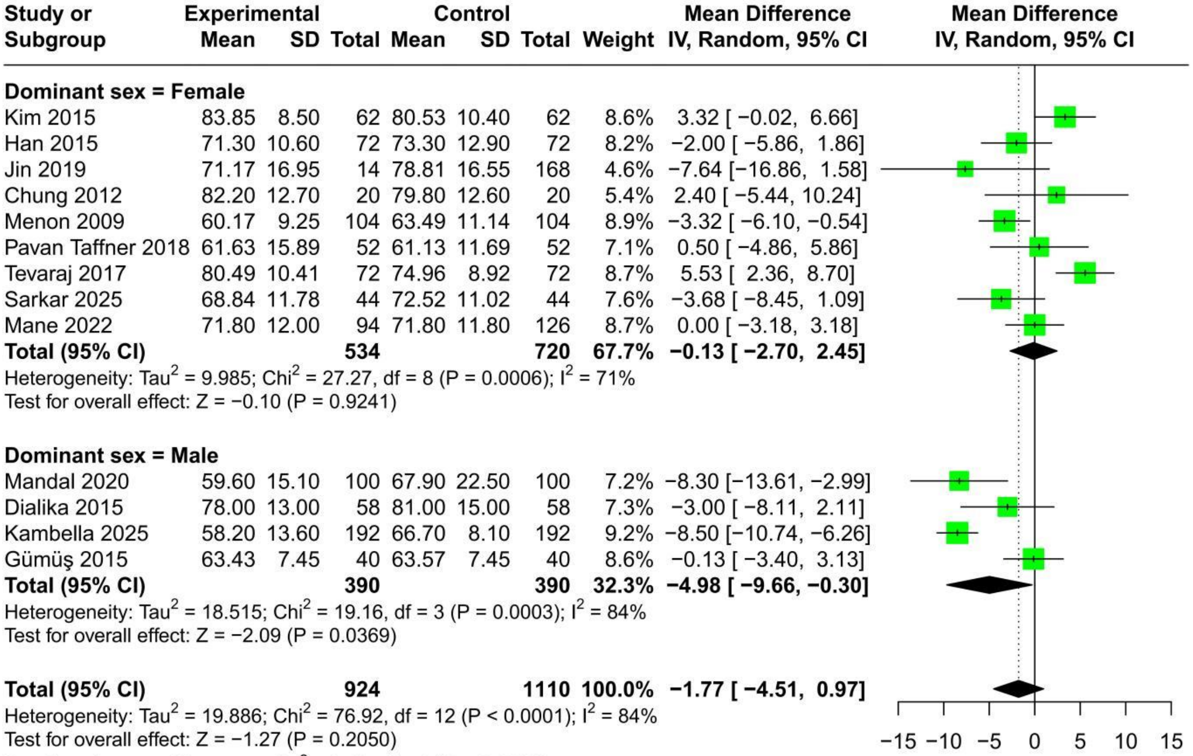

Figure S2. Continue.

E **iii. Mean age**

Study or Subgroup	Experimental Mean	SD	Total	Control Mean	SD	Total	Weight	Mean Difference IV, Random, 95% CI
**Mean age = Less than 40**								
Chung 2012	82.20	12.70	20	79.80	12.60	20	4.9%	2.40 [−5.44, 10.24]
Mandal 2020	59.60	15.10	100	67.90	22.50	100	6.6%	−8.30 [−13.61, −2.99]
Menon 2009	60.17	9.25	104	63.49	11.14	104	8.3%	−3.32 [−6.10, −0.54]
Dialika 2015	78.00	13.00	58	81.00	15.00	58	6.7%	−3.00 [−8.11, 2.11]
Sarkar 2025	68.84	11.78	44	72.52	11.02	44	7.0%	−3.68 [−8.45, 1.09]
Mane 2022	71.80	12.00	94	71.80	11.80	126	8.1%	0.00 [−3.18, 3.18]
Gümüş 2015	63.43	7.45	40	63.57	7.45	40	8.0%	−0.13 [−3.40, 3.13]
**Total (95% CI)**			**460**			**492**	**49.6%**	**−2.29 [−4.40, −0.17]**
Heterogeneity: $Tau^2 = 3.431$; $Chi^2 = 10.84$, df = 6 (P = 0.0933); $I^2 = 45\%$								
Test for overall effect: Z = −2.12 (P = 0.0339)								
**Mean age = 40 or more**								
Kim 2015	83.85	8.50	62	80.53	10.40	62	8.0%	3.32 [−0.02, 6.66]
Han 2015	71.30	10.60	72	73.30	12.90	72	7.6%	−2.00 [−5.86, 1.86]
Jin 2019	71.17	16.95	14	78.81	16.55	168	4.2%	−7.64 [−16.86, 1.58]
Pavan Taffner 2018	61.63	15.89	52	61.13	11.69	52	6.5%	0.50 [−4.86, 5.86]
Tevaraj 2017	80.49	10.41	72	74.96	8.92	72	8.1%	5.53 [2.36, 8.70]
Kambella 2025	58.20	13.60	192	66.70	8.10	192	8.6%	−8.50 [−10.74, −6.26]
Findik 2019	63.44	8.44	28	65.52	7.46	28	7.4%	−2.08 [−6.25, 2.09]
**Total (95% CI)**			**492**			**646**	**50.4%**	**−1.31 [−6.06, 3.44]**
Heterogeneity: $Tau^2 = 35.509$; $Chi^2 = 66.08$, df = 6 (P < 0.0001); $I^2 = 91\%$								
Test for overall effect: Z = −0.54 (P = 0.5892)								
**Total (95% CI)**			**952**			**1138**	**100.0%**	**−1.79 [−4.33, 0.76]**
Heterogeneity: $Tau^2 = 18.282$; $Chi^2 = 76.92$, df = 13 (P < 0.0001); $I^2 = 83\%$								
Test for overall effect: Z = −1.38 (P = 0.1689)								
Test for subgroup differences: $Chi^2 = 0.14$, df = 1 (P = 0.7117)								

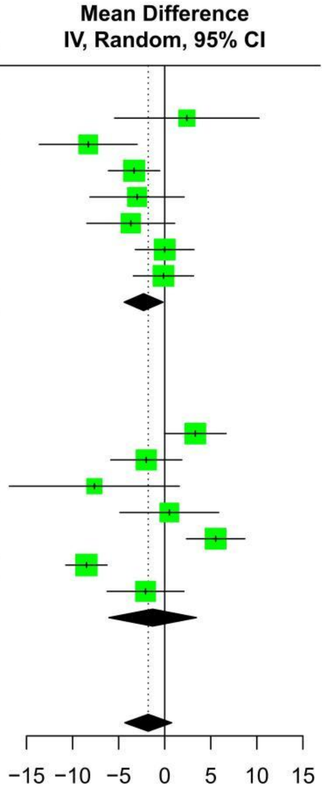

iiii. Device

Study or Subgroup	Experimental Mean	SD	Total	Control Mean	SD	Total	Weight	Mean Difference IV, Random, 95% CI
**Device = C**								
Han 2015	71.30	10.60	72	73.30	12.90	72	10.2%	−2.00 [−5.86, 1.86]
Jin 2019	71.17	16.95	14	78.81	16.55	168	4.7%	−7.64 [−16.86, 1.58]
Mandal 2020	59.60	15.10	100	67.90	22.50	100	8.3%	−8.30 [−13.61, −2.99]
Pavan Taffner 2018	61.63	15.89	52	61.13	11.69	52	8.3%	0.50 [−4.86, 5.86]
Findik 2019	63.44	8.44	28	65.52	7.46	28	9.8%	−2.08 [−6.25, 2.09]
Gümüş 2015	63.43	7.45	40	63.57	7.45	40	11.0%	−0.13 [−3.40, 3.13]
**Total (95% CI)**			**306**			**460**	**52.2%**	**−2.49 [−5.05, 0.07]**
Heterogeneity: $Tau^2 = 4.316$; $Chi^2 = 8.92$, df = 5 (P = 0.1122); $I^2 = 44\%$								
Test for overall effect: Z = −1.90 (P = 0.0568)								
**Device = S**								
Kim 2015	83.85	8.50	62	80.53	10.40	62	10.9%	3.32 [−0.02, 6.66]
Chung 2012	82.20	12.70	20	79.80	12.60	20	5.7%	2.40 [−5.44, 10.24]
Dialika 2015	78.00	13.00	58	81.00	15.00	58	8.6%	−3.00 [−8.11, 2.11]
**Total (95% CI)**			**140**			**140**	**25.1%**	**1.01 [−3.26, 5.29]**
Heterogeneity: $Tau^2 = 7.386$; $Chi^2 = 4.17$, df = 2 (P = 0.1244); $I^2 = 52\%$								
Test for overall effect: Z = 0.47 (P = 0.6416)								
**Device = Other devices**								
Menon 2009	60.17	9.25	104	63.49	11.14	104	11.6%	−3.32 [−6.10, −0.54]
Tevaraj 2017	80.49	10.41	72	74.96	8.92	72	11.1%	5.53 [2.36, 8.70]
**Total (95% CI)**			**176**			**176**	**22.7%**	**1.07 [−7.60, 9.74]**
Heterogeneity: $Tau^2 = 36.848$; $Chi^2 = 16.93$, df = 1 (P < 0.0001); $I^2 = 94\%$								
Test for overall effect: Z = 0.24 (P = 0.8087)								
**Total (95% CI)**			**622**			**776**	**100.0%**	**−0.96 [−3.41, 1.50]**
Heterogeneity: $Tau^2 = 11.553$; $Chi^2 = 36.22$, df = 10 (P < 0.0001); $I^2 = 72\%$								
Test for overall effect: Z = −0.76 (P = 0.4453)								
Test for subgroup differences: $Chi^2 = 2.23$, df = 2 (P = 0.3272)								

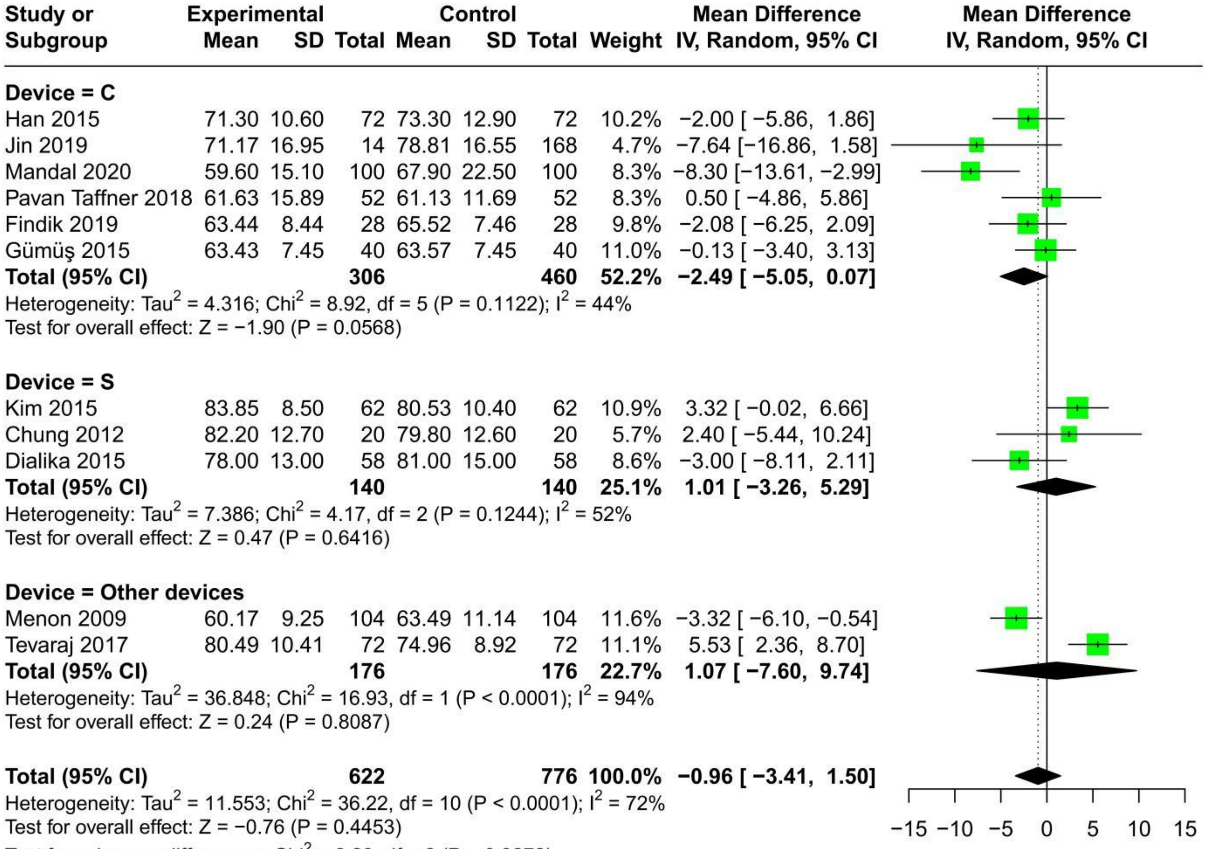

Figure S2. Subgroup analysis of RNFL thicknesses. This figure shows subgroup analysis of RNFL thicknesses in (A) average and in the (B) superior, (C) inferior, (D) nasal, and (E) temporal subsectors after grouped by (i) countries, (ii) dominant sexes, (iii) mean age groups, and (iiii) devices. The sizes of green squares, black crosses, and vertical lines indicate the weights, mean differences, and 95% CIs in included studies, respectively. The dashed lines indicate the standardized mean differences obtained after meta-analysis. The sample sizes refer to the numbers of eyes. RNFL = retinal nerve fiber layer, SD = standard deviation, IV = inverse variance, CI = confidence interval.

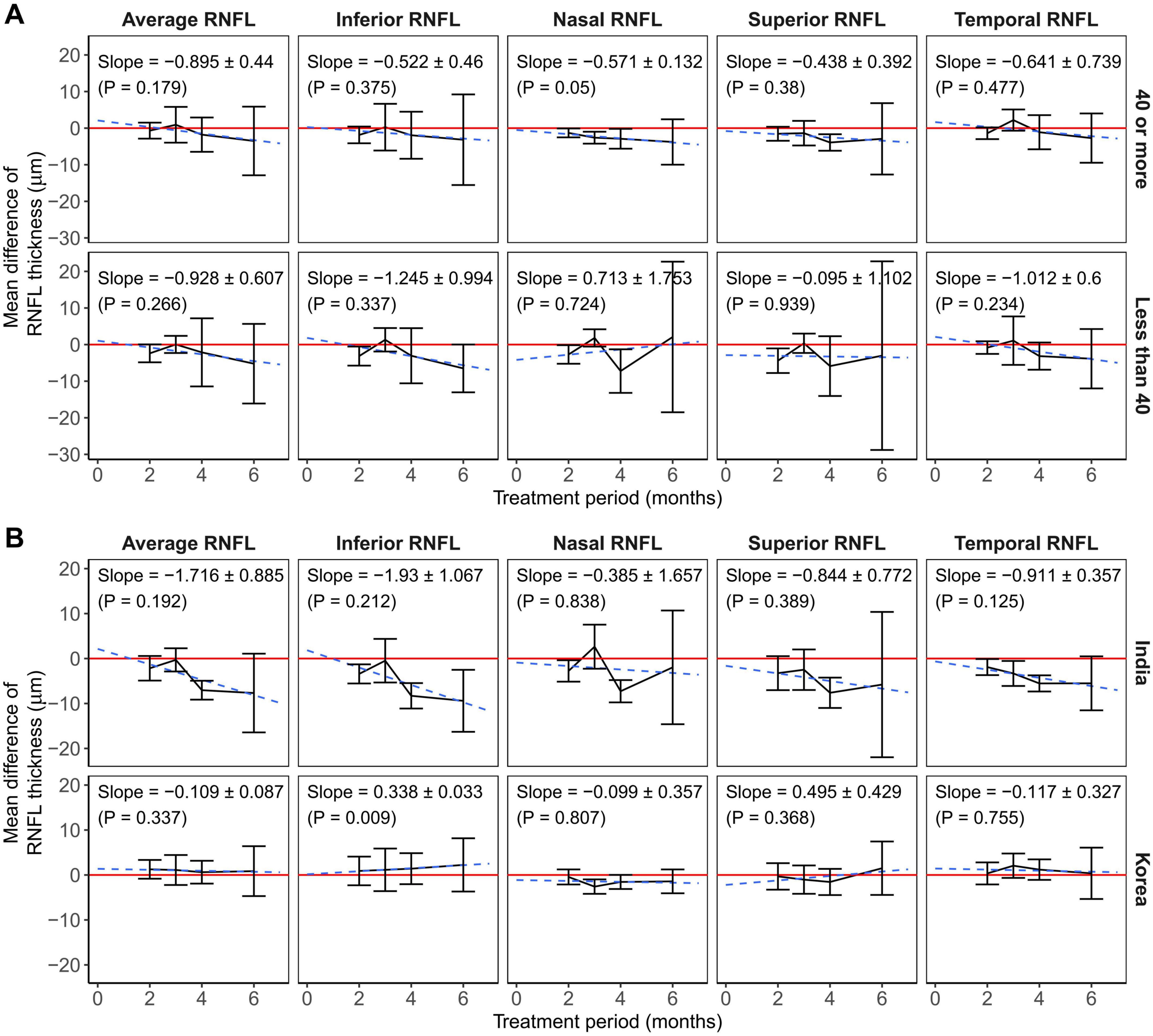

Figure S3. Meta-regression of RNFL thicknesses in studies with different mean ages and contries. This figure shows the relationship between RNFL thicknesses and the period of the ethambutol administration in studies with different (A) mean ages and (B) contries. The lines connect the estimation of mean differences of RNFL thicknesses, and the error bars show the 95 % CI obtained in the meta-analysis. Red lines highlight the difference of 0. Blue dashed lines refer to the results of the linear regression, and the slopes of them are shown at the top of the figures as estimations ± standard errors, followed by the P values. RNFL = retinal nerve fiber layer, OCT = optical coherence tomography.

A **i. Average RNFL**

Study	Experimental Mean	Experimental SD	Experimental Total	Control Mean	Control SD	Control Total	Weight	Mean Difference IV, Random, 95% CI
Kim 2015	109.04	9.10	62	106.30	8.70	62	11.1%	2.74 [−0.39, 5.87]
Jin 2019	101.51	13.43	124	101.53	13.89	168	11.0%	−0.02 [−3.18, 3.14]
Chung 2012	107.40	9.80	20	107.00	9.80	20	4.8%	0.40 [−5.67, 6.47]
Mandal 2020	95.20	13.50	100	100.80	16.10	100	8.3%	−5.60 [−9.72, −1.48]
Pavan Taffner 2018	91.65	13.39	52	91.71	13.10	52	6.3%	−0.06 [−5.15, 5.03]
Dialika 2015	112.65	10.63	58	116.77	11.69	58	8.4%	−4.12 [−8.19, −0.05]
Mane 2022	101.10	7.70	98	100.90	8.60	126	14.7%	0.20 [−1.94, 2.34]
Kambella 2025	97.50	11.20	192	99.90	7.30	192	15.7%	−2.40 [−4.29, −0.51]
Findik 2019	92.96	7.40	28	96.16	7.25	28	9.0%	−3.21 [−7.04, 0.63]
Gümüş 2015	92.70	7.45	40	95.87	7.45	40	10.7%	−3.17 [−6.43, 0.10]
**Total (95% CI)**			**774**			**846**	**100.0%**	**−1.47 [−2.99, 0.06]**

Heterogeneity: $Tau^2 = 2.926$; $Chi^2 = 18.95$, df = 9 (P = 0.0256); $I^2 = 53\%$
Test for overall effect: Z = −1.88 (P = 0.0599)

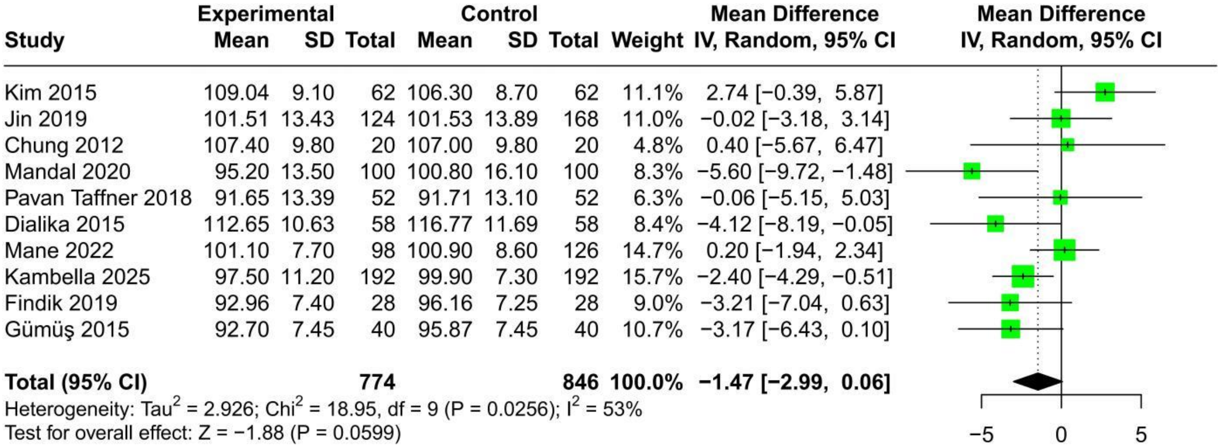

ii. Superior RNFL

Study	Experimental Mean	Experimental SD	Experimental Total	Control Mean	Control SD	Control Total	Weight	Mean Difference IV, Random, 95% CI
Kim 2015	135.87	14.10	62	136.10	12.30	62	11.2%	−0.23 [−4.89, 4.43]
Jin 2019	126.14	19.20	124	126.56	16.85	168	12.7%	−0.42 [−4.65, 3.81]
Chung 2012	131.40	13.80	20	131.60	14.20	20	4.1%	−0.20 [−8.88, 8.48]
Mandal 2020	120.80	22.90	100	129.80	24.90	100	6.6%	−9.00 [−15.63, −2.37]
Pavan Taffner 2018	112.12	21.46	52	112.90	22.51	52	4.3%	−0.78 [−9.23, 7.67]
Dialika 2015	141.00	16.00	58	147.00	16.00	58	8.1%	−6.00 [−11.82, −0.18]
Sarkar 2025	114.10	15.98	44	120.25	15.03	44	6.8%	−6.15 [−12.63, 0.33]
Mane 2022	135.00	18.10	98	134.00	17.60	126	11.0%	1.00 [−3.72, 5.72]
Kambella 2025	120.90	15.80	192	122.70	11.10	192	20.5%	−1.80 [−4.53, 0.93]
Findik 2019	117.37	11.95	28	122.94	12.45	28	7.0%	−5.57 [−11.97, 0.82]
Gümüş 2015	116.11	11.47	40	122.66	15.48	40	7.8%	−6.55 [−12.52, −0.58]
**Total (95% CI)**			**818**			**890**	**100.0%**	**−2.77 [−4.64, −0.89]**

Heterogeneity: $Tau^2 = 2.521$; $Chi^2 = 13.59$, df = 10 (P = 0.1926); $I^2 = 26\%$
Test for overall effect: Z = −2.89 (P = 0.0038)

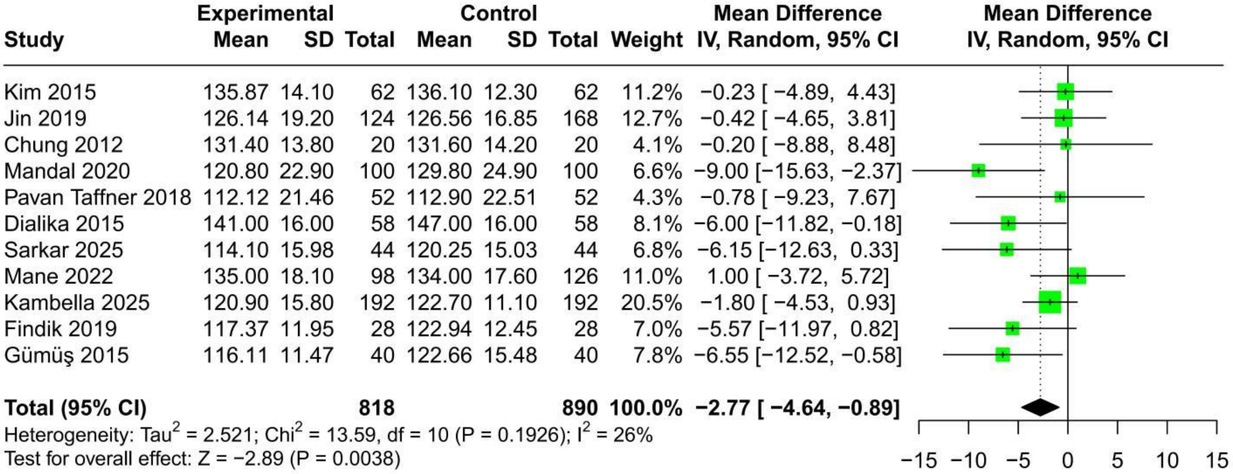

iii. Inferior RNFL

Study	Experimental Mean	Experimental SD	Experimental Total	Control Mean	Control SD	Control Total	Weight	Mean Difference IV, Random, 95% CI
Kim 2015	138.60	13.50	62	137.21	13.10	62	11.5%	1.39 [−3.29, 6.07]
Jin 2019	130.01	22.00	124	129.55	21.59	168	9.8%	0.46 [−4.60, 5.52]
Chung 2012	137.10	11.60	20	136.60	15.60	20	3.5%	0.50 [−8.02, 9.02]
Mandal 2020	122.10	22.80	100	126.10	24.60	100	5.8%	−4.00 [−10.57, 2.57]
Pavan Taffner 2018	118.48	22.49	52	121.56	21.61	52	3.5%	−3.08 [−11.56, 5.40]
Dialika 2015	144.00	19.00	58	146.00	21.00	58	4.7%	−2.00 [−9.29, 5.29]
Sarkar 2025	116.20	17.27	44	123.40	32.27	44	2.2%	−7.20 [−18.01, 3.61]
Mane 2022	135.40	14.80	98	138.30	16.90	126	14.6%	−2.90 [−7.06, 1.26]
Kambella 2025	116.30	16.00	192	119.60	11.20	192	33.0%	−3.30 [−6.06, −0.54]
Findik 2019	113.99	15.15	28	119.23	11.88	28	5.0%	−5.25 [−12.38, 1.89]
Gümüş 2015	118.92	12.89	40	123.27	15.48	40	6.5%	−4.36 [−10.60, 1.88]
**Total (95% CI)**			**818**			**890**	**100.0%**	**−2.42 [−4.01, −0.83]**

Heterogeneity: $Tau^2 = 0$; $Chi^2 = 6.66$, df = 10 (P = 0.7572); $I^2 = 0\%$
Test for overall effect: Z = −2.99 (P = 0.0028)

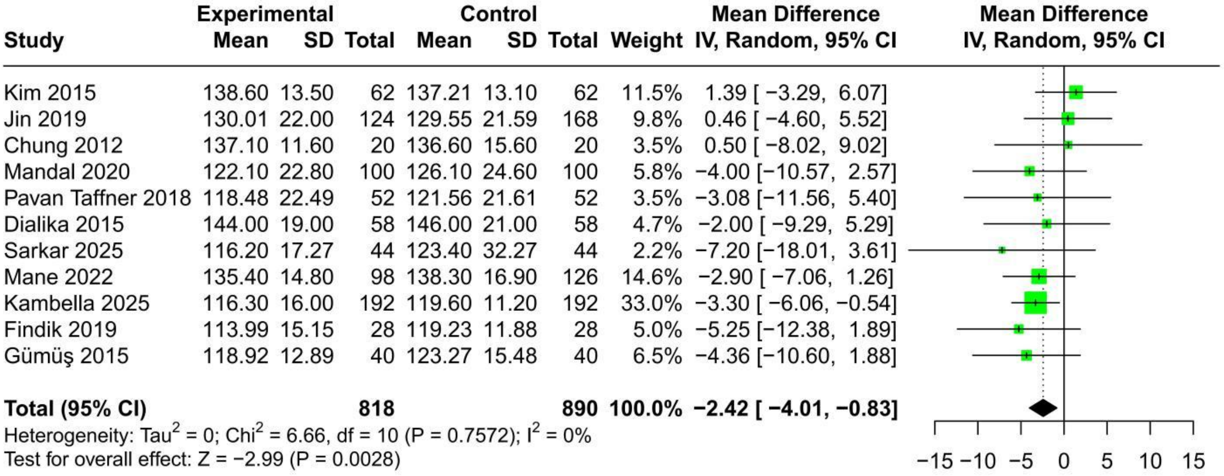

Figure S4. Continue.

A

iiii. Nasal RNFL

Study	Experimental Mean	Experimental SD	Experimental Total	Control Mean	Control SD	Control Total	Weight	Mean Difference IV, Random, 95% CI
Kim 2015	80.41	4.50	62	80.50	6.50	62	31.0%	−0.09 [−2.06, 1.88]
Jin 2019	71.62	13.71	124	73.02	15.34	168	10.7%	−1.40 [−4.75, 1.95]
Chung 2012	79.40	13.70	20	80.40	19.90	20	1.1%	−1.00 [−11.59, 9.59]
Mandal 2020	74.20	16.10	100	79.50	27.10	100	3.1%	−5.30 [−11.48, 0.88]
Pavan Taffner 2018	71.04	13.01	52	73.63	12.40	52	5.0%	−2.59 [−7.47, 2.29]
Dialika 2015	88.00	18.00	58	92.00	22.00	58	2.2%	−4.00 [−11.32, 3.32]
Sarkar 2025	79.05	13.90	44	85.48	14.38	44	3.4%	−6.43 [−12.34, −0.52]
Mane 2022	87.00	14.20	98	86.30	26.00	126	4.2%	0.70 [−4.64, 6.04]
Kambella 2025	80.30	10.90	192	82.60	8.70	192	30.9%	−2.30 [−4.27, −0.33]
Findik 2019	73.59	11.25	28	74.73	10.48	28	3.7%	−1.15 [−6.84, 4.55]
Gümüş 2015	71.00	12.00	40	72.00	11.47	40	4.5%	−1.00 [−6.14, 4.14]
**Total (95% CI)**			**818**			**890**	**100.0%**	**−1.57 [−2.66, −0.47]**

Heterogeneity: $Tau^2 = 0$; $Chi^2 = 8.07$, df = 10 (P = 0.6218); $I^2 = 0\%$

Test for overall effect: Z = −2.80 (P = 0.0051)

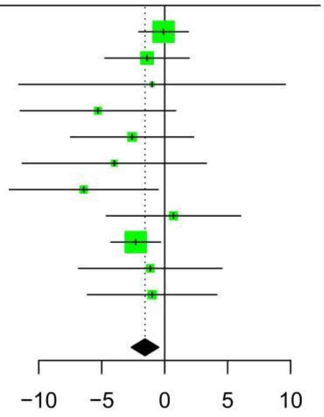

iiiii. Temporal RNFL

Study	Experimental Mean	Experimental SD	Experimental Total	Control Mean	Control SD	Control Total	Weight	Mean Difference IV, Random, 95% CI
Kim 2015	81.37	9.80	62	80.53	10.40	62	9.7%	0.84 [−2.72, 4.40]
Jin 2019	78.61	16.06	124	78.81	16.55	168	8.6%	−0.20 [−3.98, 3.58]
Chung 2012	80.10	11.70	20	79.80	12.60	20	2.2%	0.30 [−7.24, 7.84]
Mandal 2020	64.40	13.10	100	67.90	22.50	100	4.7%	−3.50 [−8.60, 1.60]
Pavan Taffner 2018	61.63	15.89	52	61.13	11.69	52	4.3%	0.50 [−4.86, 5.86]
Dialika 2015	78.00	13.00	58	81.00	15.00	58	4.7%	−3.00 [−8.11, 2.11]
Sarkar 2025	70.86	12.04	44	72.52	11.02	44	5.3%	−1.66 [−6.48, 3.16]
Mane 2022	72.30	11.70	98	71.80	11.80	126	12.7%	0.50 [−2.60, 3.60]
Kambella 2025	63.80	11.90	192	66.70	8.10	192	29.5%	−2.90 [−4.94, −0.86]
Findik 2019	63.44	8.44	28	65.52	7.46	28	7.0%	−2.08 [−6.25, 2.09]
Gümüş 2015	63.43	7.45	40	63.57	7.45	40	11.5%	−0.13 [−3.40, 3.13]
**Total (95% CI)**			**818**			**890**	**100.0%**	**−1.25 [−2.36, −0.15]**

Heterogeneity: $Tau^2 = 0$; $Chi^2 = 7.77$, df = 10 (P = 0.6515); $I^2 = 0\%$

Test for overall effect: Z = −2.22 (P = 0.0263)

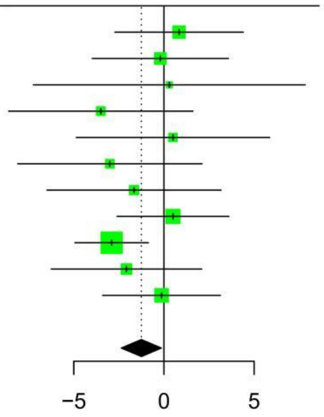

Figure S4. Continue.

B **i. Average RNFL**

Study	Experimental Mean	Experimental SD	Experimental Total	Control Mean	Control SD	Control Total	Weight	Mean Difference IV, Random, 95% CI
Kim 2015	109.65	9.10	62	106.30	8.70	62	28.1%	3.35 [0.22, 6.48]
Jin 2019	99.89	13.41	88	101.53	13.89	168	24.8%	−1.64 [−5.14, 1.86]
Chung 2012	108.60	8.00	20	107.00	9.80	20	13.1%	1.60 [−3.94, 7.14]
Menon 2009	96.93	9.37	104	97.23	9.63	104	34.0%	−0.30 [−2.88, 2.28]
**Total (95% CI)**			**274**			**354**	**100.0%**	**0.64 [−1.62, 2.90]**

Heterogeneity: $Tau^2 = 2.166$; $Chi^2 = 5.12$, df = 3 (P = 0.1631); $I^2 = 41\%$
Test for overall effect: Z = 0.56 (P = 0.5772)

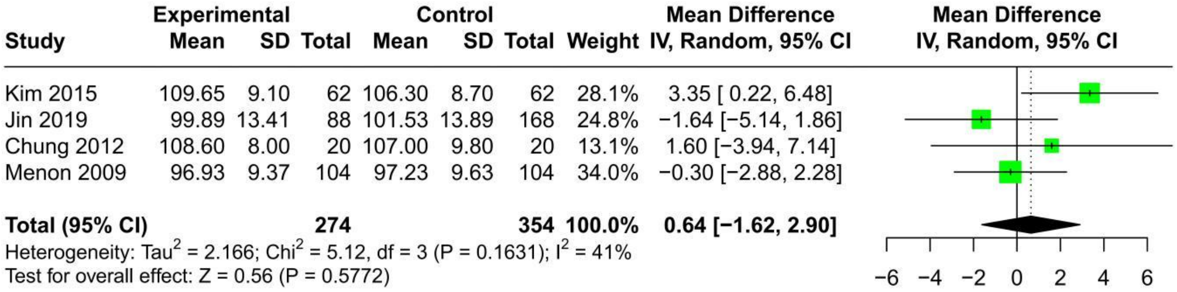

ii. Superior RNFL

Study	Experimental Mean	Experimental SD	Experimental Total	Control Mean	Control SD	Control Total	Weight	Mean Difference IV, Random, 95% CI
Kim 2015	136.17	13.80	62	136.10	12.30	62	17.2%	0.07 [−4.53, 4.67]
Jin 2019	123.54	20.12	88	126.56	16.85	168	15.1%	−3.02 [−7.94, 1.90]
Chung 2012	133.10	15.40	20	131.60	14.20	20	4.3%	1.50 [−7.68, 10.68]
Menon 2009	123.87	16.14	104	126.36	17.03	104	17.9%	−2.49 [−7.00, 2.02]
Tevaraj 2017	134.42	8.16	72	132.85	9.17	72	45.4%	1.57 [−1.27, 4.41]
**Total (95% CI)**			**346**			**426**	**100.0%**	**−0.11 [−2.02, 1.80]**

Heterogeneity: $Tau^2 = 0$; $Chi^2 = 3.89$, df = 4 (P = 0.4212); $I^2 = 0\%$
Test for overall effect: Z = −0.12 (P = 0.9075)

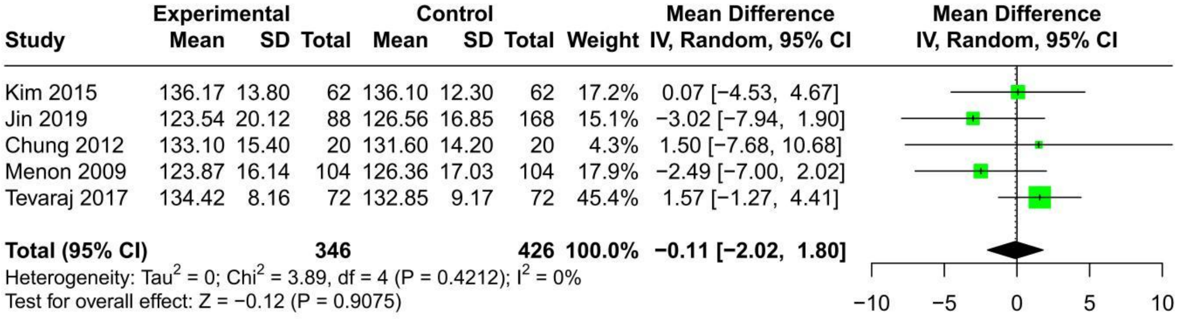

iii. Inferior RNFL

Study	Experimental Mean	Experimental SD	Experimental Total	Control Mean	Control SD	Control Total	Weight	Mean Difference IV, Random, 95% CI
Kim 2015	140.56	14.10	62	137.21	13.10	62	25.1%	3.35 [−1.44, 8.14]
Jin 2019	126.38	21.95	88	129.55	21.59	168	18.5%	−3.17 [−8.80, 2.46]
Chung 2012	140.90	13.40	20	136.60	15.60	20	7.4%	4.30 [−4.71, 13.31]
Menon 2009	124.86	18.04	104	125.34	17.63	104	24.6%	−0.48 [−5.33, 4.37]
Tevaraj 2017	128.83	14.88	72	126.58	14.87	72	24.5%	2.25 [−2.61, 7.11]
**Total (95% CI)**			**346**			**426**	**100.0%**	**1.01 [−1.46, 3.47]**

Heterogeneity: $Tau^2 = 0.310$; $Chi^2 = 4.16$, df = 4 (P = 0.3849); $I^2 = 4\%$
Test for overall effect: Z = 0.80 (P = 0.4234)

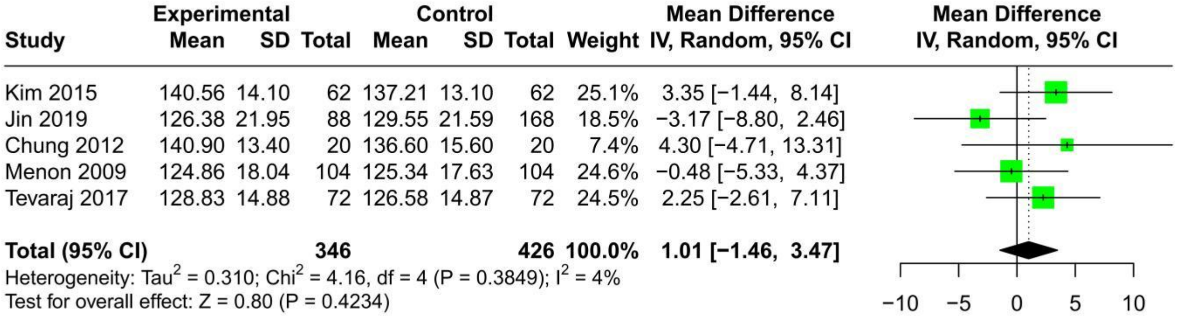

iiii. Nasal RNFL

Study	Experimental Mean	Experimental SD	Experimental Total	Control Mean	Control SD	Control Total	Weight	Mean Difference IV, Random, 95% CI
Kim 2015	77.98	3.20	62	80.50	6.50	62	32.1%	−2.52 [−4.32, −0.72]
Jin 2019	70.02	14.20	88	73.02	15.34	168	20.8%	−3.00 [−6.77, 0.77]
Chung 2012	78.60	12.30	20	80.40	19.90	20	5.3%	−1.80 [−12.05, 8.45]
Menon 2009	75.13	19.31	104	72.50	16.67	104	15.7%	2.63 [−2.27, 7.53]
Tevaraj 2017	81.18	8.07	72	79.36	9.03	72	26.1%	1.82 [−0.98, 4.62]
**Total (95% CI)**			**346**			**426**	**100.0%**	**−0.64 [−3.17, 1.89]**

Heterogeneity: $Tau^2 = 4.346$; $Chi^2 = 9.77$, df = 4 (P = 0.0445); $I^2 = 59\%$
Test for overall effect: Z = −0.49 (P = 0.6216)

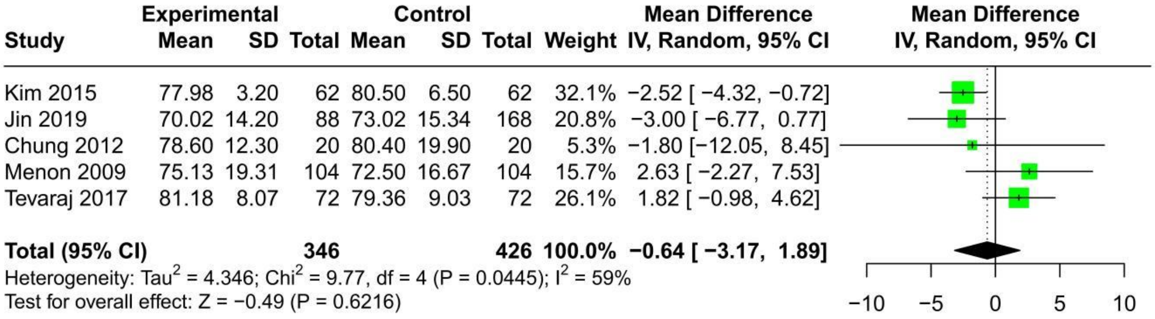

iiiii. Temporal RNFL

Study	Experimental Mean	Experimental SD	Experimental Total	Control Mean	Control SD	Control Total	Weight	Mean Difference IV, Random, 95% CI
Kim 2015	83.17	10.70	62	80.53	10.40	62	21.5%	2.64 [−1.07, 6.35]
Jin 2019	80.32	18.68	88	78.81	16.55	168	19.2%	1.51 [−3.13, 6.15]
Chung 2012	80.90	12.20	20	79.80	12.60	20	12.7%	1.10 [−6.59, 8.79]
Menon 2009	60.17	9.25	104	63.49	11.14	104	23.7%	−3.32 [−6.10, −0.54]
Tevaraj 2017	80.49	10.41	72	74.96	8.92	72	22.8%	5.53 [2.36, 8.70]
**Total (95% CI)**			**346**			**426**	**100.0%**	**1.47 [−2.27, 5.22]**

Heterogeneity: $Tau^2 = 13.367$; $Chi^2 = 17.90$, df = 4 (P = 0.0013); $I^2 = 78\%$
Test for overall effect: Z = 0.77 (P = 0.4404)

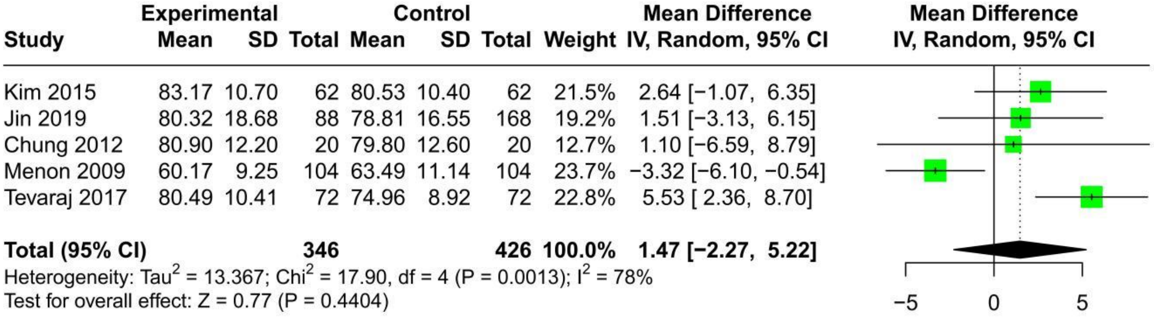

Figure S4. Continue.

C **i. Average RNFL**

Study	Experimental Mean	Experimental SD	Experimental Total	Control Mean	Control SD	Control Total	Weight	Mean Difference IV, Random, 95% CI
Kim 2015	109.31	7.80	62	106.30	8.70	62	17.8%	3.01 [0.10, 5.92]
Han 2015	94.20	9.90	72	95.60	10.40	72	17.3%	−1.40 [−4.72, 1.92]
Jin 2019	100.11	13.19	76	101.53	13.89	168	16.8%	−1.42 [−5.05, 2.21]
Chung 2012	109.70	7.10	20	107.00	9.80	20	14.3%	2.70 [−2.60, 8.00]
Mandal 2020	94.00	17.90	100	100.80	16.10	100	15.2%	−6.80 [−11.52, −2.08]
Kambella 2025	92.80	14.90	192	99.90	7.30	192	18.5%	−7.10 [−9.45, −4.75]
**Total (95% CI)**			**522**			**614**	**100.0%**	**−1.91 [−5.71, 1.90]**

Heterogeneity: $Tau^2 = 18.906$; $Chi^2 = 36.19$, df = 5 ($P < 0.0001$); $I^2 = 86\%$
Test for overall effect: Z = −0.98 (P = 0.3258)

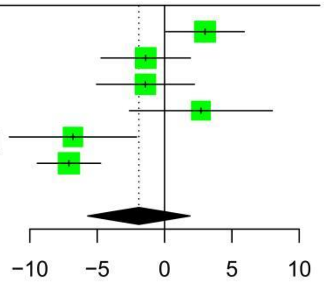

ii. Superior RNFL

Study	Experimental Mean	Experimental SD	Experimental Total	Control Mean	Control SD	Control Total	Weight	Mean Difference IV, Random, 95% CI
Kim 2015	135.17	15.20	62	136.10	12.30	62	16.5%	−0.93 [−5.80, 3.94]
Han 2015	117.60	19.30	72	119.80	20.40	72	12.4%	−2.20 [−8.69, 4.29]
Jin 2019	122.87	19.95	76	126.56	16.85	168	15.7%	−3.69 [−8.85, 1.47]
Chung 2012	134.90	12.80	20	131.60	14.20	20	9.0%	3.30 [−5.08, 11.68]
Mandal 2020	118.10	20.90	100	129.80	24.90	100	12.7%	−11.70 [−18.07, −5.33]
Sarkar 2025	112.20	16.67	44	120.25	15.03	44	12.1%	−8.05 [−14.68, −1.42]
Kambella 2025	116.90	20.30	192	122.70	11.10	192	21.5%	−5.80 [−9.07, −2.53]
**Total (95% CI)**			**566**			**658**	**100.0%**	**−4.42 [−7.45, −1.38]**

Heterogeneity: $Tau^2 = 8.337$; $Chi^2 = 12.57$, df = 6 ($P = 0.0504$); $I^2 = 52\%$
Test for overall effect: Z = −2.85 (P = 0.0043)

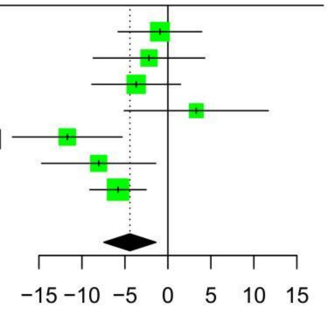

iii. Inferior RNFL

Study	Experimental Mean	Experimental SD	Experimental Total	Control Mean	Control SD	Control Total	Weight	Mean Difference IV, Random, 95% CI
Kim 2015	141.59	12.90	62	137.21	13.10	62	16.5%	4.38 [−0.20, 8.96]
Han 2015	122.60	17.40	72	123.20	17.60	72	15.2%	−0.60 [−6.32, 5.12]
Jin 2019	127.34	20.97	76	129.55	21.59	168	15.1%	−2.21 [−7.94, 3.52]
Chung 2012	141.20	13.00	20	136.60	15.60	20	11.4%	4.60 [−4.30, 13.50]
Mandal 2020	118.90	21.40	100	126.10	24.60	100	14.3%	−7.20 [−13.59, −0.81]
Sarkar 2025	117.50	17.48	44	123.40	32.27	44	9.5%	−5.90 [−16.74, 4.94]
Kambella 2025	110.80	20.40	192	119.60	11.20	192	17.9%	−8.80 [−12.09, −5.51]
**Total (95% CI)**			**566**			**658**	**100.0%**	**−2.34 [−7.00, 2.31]**

Heterogeneity: $Tau^2 = 28.731$; $Chi^2 = 27.19$, df = 6 ($P = 0.0001$); $I^2 = 78\%$
Test for overall effect: Z = −0.99 (P = 0.3238)

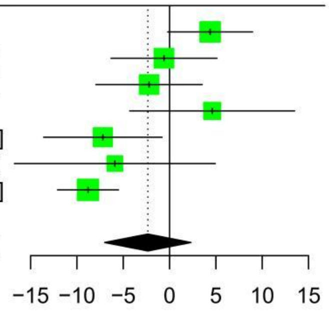

iiii. Nasal RNFL

Study	Experimental Mean	Experimental SD	Experimental Total	Control Mean	Control SD	Control Total	Weight	Mean Difference IV, Random, 95% CI
Kim 2015	79.25	6.70	62	80.50	6.50	62	20.1%	−1.25 [−3.57, 1.07]
Han 2015	63.30	8.20	72	65.60	8.00	72	19.2%	−2.30 [−4.95, 0.35]
Jin 2019	71.97	13.50	76	73.02	15.34	168	15.8%	−1.05 [−4.87, 2.77]
Chung 2012	81.10	15.80	20	80.40	19.90	20	4.5%	0.70 [−10.44, 11.84]
Mandal 2020	72.60	13.60	100	79.50	27.10	100	10.8%	−6.90 [−12.84, −0.96]
Sarkar 2025	73.61	16.97	44	85.48	14.38	44	9.6%	−11.87 [−18.44, −5.30]
Kambella 2025	76.10	13.90	192	82.60	8.70	192	20.1%	−6.50 [−8.82, −4.18]
**Total (95% CI)**			**566**			**658**	**100.0%**	**−4.01 [−6.64, −1.38]**

Heterogeneity: $Tau^2 = 7.603$; $Chi^2 = 20.41$, df = 6 ($P = 0.0023$); $I^2 = 71\%$
Test for overall effect: Z = −2.98 (P = 0.0028)

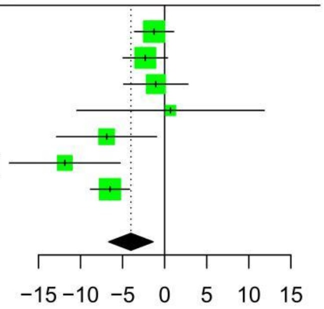

iiiii. Temporal RNFL

Study	Experimental Mean	Experimental SD	Experimental Total	Control Mean	Control SD	Control Total	Weight	Mean Difference IV, Random, 95% CI
Kim 2015	83.60	9.90	62	80.53	10.40	62	16.2%	3.07 [−0.50, 6.64]
Han 2015	72.80	12.00	72	73.30	12.90	72	15.2%	−0.50 [−4.57, 3.57]
Jin 2019	78.34	20.97	76	78.81	16.55	168	12.8%	−0.47 [−5.81, 4.87]
Chung 2012	81.70	10.80	20	79.80	12.60	20	9.7%	1.90 [−5.37, 9.17]
Mandal 2020	62.40	13.20	100	67.90	22.50	100	13.3%	−5.50 [−10.61, −0.39]
Sarkar 2025	68.84	11.78	44	72.52	11.02	44	13.9%	−3.68 [−8.45, 1.09]
Kambella 2025	60.80	12.40	192	66.70	8.10	192	18.8%	−5.90 [−8.00, −3.80]
**Total (95% CI)**			**566**			**658**	**100.0%**	**−1.81 [−4.93, 1.31]**

Heterogeneity: $Tau^2 = 12.300$; $Chi^2 = 23.49$, df = 6 ($P = 0.0006$); $I^2 = 74\%$
Test for overall effect: Z = −1.14 (P = 0.2563)

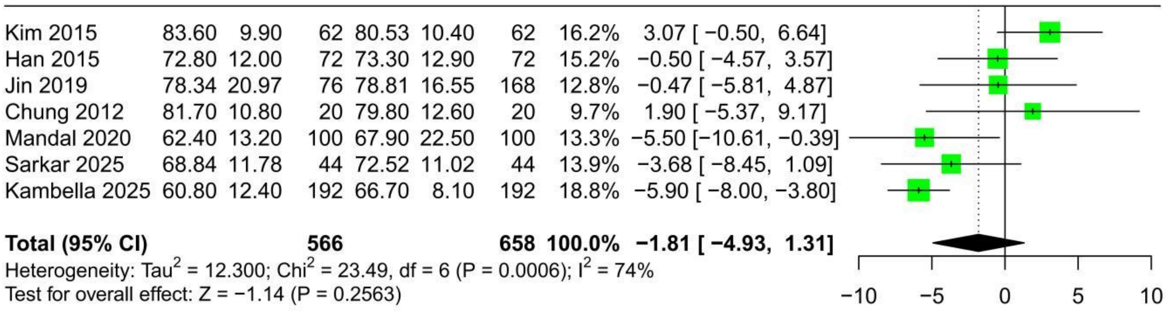

Figure S4. Continue.

D

i. Average RNFL

Study	Experimental Mean	Experimental SD	Experimental Total	Control Mean	Control SD	Control Total	Weight	Mean Difference IV, Random, 95% CI
Han 2015	94.00	8.10	72	95.60	10.40	72	20.4%	−1.60 [−4.64, 1.44]
Jin 2019	105.65	15.38	40	101.53	13.89	168	18.6%	4.12 [−1.09, 9.33]
Mandal 2020	89.90	13.80	100	100.80	16.10	100	19.6%	−10.90 [−15.06, −6.74]
Mane 2022	101.10	10.20	94	100.90	8.60	126	20.8%	0.20 [−2.35, 2.75]
Kambella 2025	87.40	18.60	192	99.90	7.30	192	20.6%	−12.50 [−15.33, −9.67]
**Total (95% CI)**			**498**			**658**	**100.0%**	**−4.23 [−10.32, 1.86]**

Heterogeneity: $Tau^2 = 44.768$; $Chi^2 = 66.93$, df = 4 ($P < 0.0001$); $I^2 = 94\%$
Test for overall effect: Z = −1.36 (P = 0.1735)

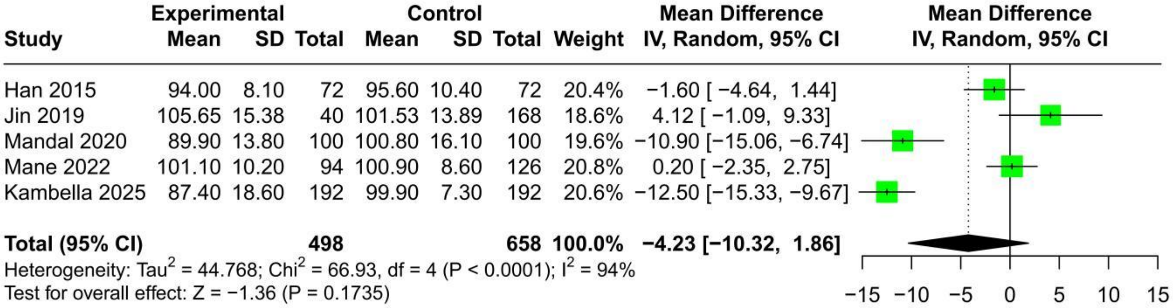

ii. Superior RNFL

Study	Experimental Mean	Experimental SD	Experimental Total	Control Mean	Control SD	Control Total	Weight	Mean Difference IV, Random, 95% CI
Han 2015	118.50	15.70	72	119.80	20.40	72	19.9%	−1.30 [−7.25, 4.65]
Jin 2019	131.34	20.38	40	126.56	16.85	168	19.5%	4.78 [−2.03, 11.59]
Mandal 2020	113.50	22.90	100	129.80	24.90	100	19.5%	−16.30 [−22.93, −9.67]
Mane 2022	144.00	15.10	94	134.00	17.60	126	20.5%	10.00 [5.67, 14.33]
Kambella 2025	111.30	27.20	192	122.70	11.10	192	20.6%	−11.40 [−15.56, −7.24]
**Total (95% CI)**			**498**			**658**	**100.0%**	**−2.81 [−12.89, 7.27]**

Heterogeneity: $Tau^2 = 123.821$; $Chi^2 = 70.78$, df = 4 ($P < 0.0001$); $I^2 = 94\%$
Test for overall effect: Z = −0.55 (P = 0.5849)

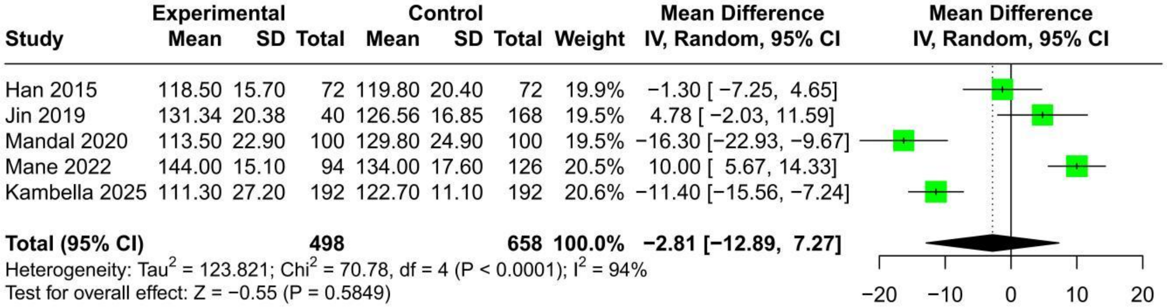

iii. Inferior RNFL

Study	Experimental Mean	Experimental SD	Experimental Total	Control Mean	Control SD	Control Total	Weight	Mean Difference IV, Random, 95% CI
Han 2015	123.00	16.50	72	123.20	17.60	72	20.2%	−0.20 [−5.77, 5.37]
Jin 2019	135.53	23.12	40	129.55	21.59	168	17.9%	5.98 [−1.89, 13.85]
Mandal 2020	115.90	21.50	100	126.10	24.60	100	19.4%	−10.20 [−16.60, −3.80]
Mane 2022	134.80	19.00	94	138.30	16.90	126	20.9%	−3.50 [−8.34, 1.34]
Kambella 2025	105.30	25.30	192	119.60	11.20	192	21.6%	−14.30 [−18.21, −10.39]
**Total (95% CI)**			**498**			**658**	**100.0%**	**−4.77 [−11.73, 2.19]**

Heterogeneity: $Tau^2 = 54.311$; $Chi^2 = 32.48$, df = 4 ($P < 0.0001$); $I^2 = 88\%$
Test for overall effect: Z = −1.34 (P = 0.1790)

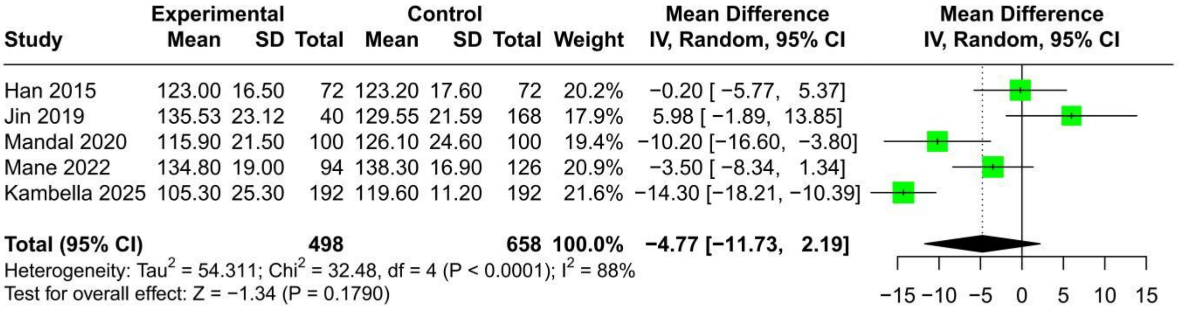

iiii. Nasal RNFL

Study	Experimental Mean	Experimental SD	Experimental Total	Control Mean	Control SD	Control Total	Weight	Mean Difference IV, Random, 95% CI
Han 2015	63.50	7.10	72	65.60	8.00	72	21.7%	−2.10 [−4.57, 0.37]
Jin 2019	74.29	16.69	40	73.02	15.34	168	19.1%	1.27 [−4.40, 6.94]
Mandal 2020	71.10	13.60	100	79.50	27.10	100	18.8%	−8.40 [−14.34, −2.46]
Mane 2022	98.90	21.00	94	86.30	26.00	126	18.5%	12.60 [6.38, 18.82]
Kambella 2025	73.10	13.90	192	82.60	8.70	192	21.8%	−9.50 [−11.82, −7.18]
**Total (95% CI)**			**498**			**658**	**100.0%**	**−1.53 [−7.91, 4.84]**

Heterogeneity: $Tau^2 = 47.118$; $Chi^2 = 56.18$, df = 4 ($P < 0.0001$); $I^2 = 93\%$
Test for overall effect: Z = −0.47 (P = 0.6374)

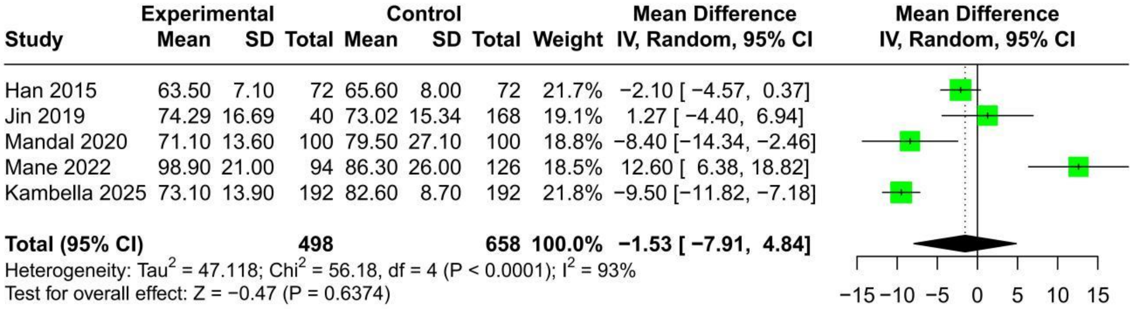

iiiii. Temporal RNFL

Study	Experimental Mean	Experimental SD	Experimental Total	Control Mean	Control SD	Control Total	Weight	Mean Difference IV, Random, 95% CI
Han 2015	71.30	10.60	72	73.30	12.90	72	20.7%	−2.00 [−5.86, 1.86]
Jin 2019	82.76	19.43	40	78.81	16.55	168	16.3%	3.95 [−2.57, 10.47]
Mandal 2020	59.60	15.10	100	67.90	22.50	100	18.3%	−8.30 [−13.61, −2.99]
Mane 2022	71.80	12.00	94	71.80	11.80	126	21.7%	0.00 [−3.18, 3.18]
Kambella 2025	58.20	13.60	192	66.70	8.10	192	22.9%	−8.50 [−10.74, −6.26]
**Total (95% CI)**			**498**			**658**	**100.0%**	**−3.24 [−7.86, 1.38]**

Heterogeneity: $Tau^2 = 22.974$; $Chi^2 = 29.84$, df = 4 ($P < 0.0001$); $I^2 = 87\%$
Test for overall effect: Z = −1.37 (P = 0.1697)

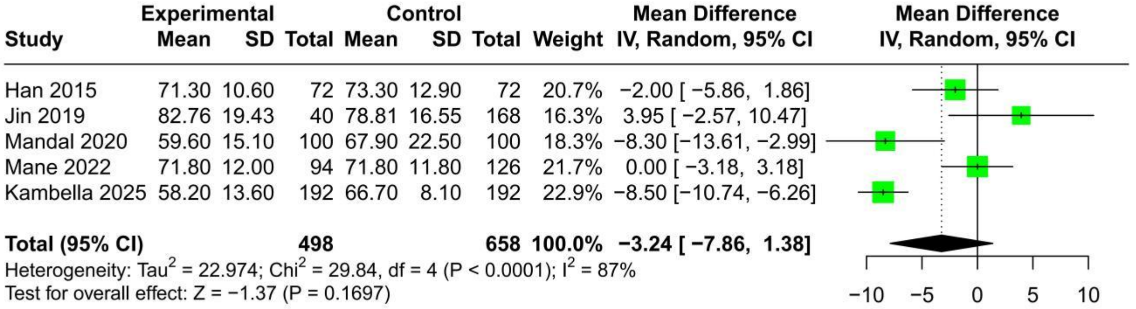

Figure S4. RNFL thicknesses after the same period of the administration of ethambutol. Meta-analysis focusing on RNFL thicknesses in average (i) and in the superior (ii), inferior (iii), nasal (iiii), and temporal (iiiii) subsectors, which were measured 2 (A), 3 (B), 4 (C), and 6 (D) months after the start of the ethambutol administration. The sizes of green squares, black crosses, and vertical lines indicate the weights, mean differences, and 95% CIs in included studies, respectively. The dashed lines indicate the standardized mean differences obtained after meta-analysis. The sample sizes refer to the numbers of eyes. RNFL = retinal nerve fiber layer, SD = standard deviation, IV = inverse variance.
